# The BRCA1 coiled-coil domain is dispensable for suppression of tandem duplications and tolerance of FANCM loss

**DOI:** 10.1038/s44318-026-00913-x

**Published:** 2026-09-18

**Authors:** Namrata M Nilavar, Alberto Marin-Gonzalez, Francesca Menghi, Daniel Nguyen, Nicholas A Willis, Ellen Wientjens, Bing Xia, Jos Jonkers, Edison T Liu, Ralph Scully

**Affiliations:** 1 Department of Medicine, Division of Hematology-Oncology and Cancer Research Institute, Beth Israel Deaconess Medical Center and Harvard Medical School, Boston, MA USA; 2 Howard Hughes Medical Institute and Program in Cellular and Molecular Medicine, Boston Children’s Hospital and Department of Pediatrics, Harvard Medical School, Boston, MA USA; 3 The Jackson Laboratory for Genomic Medicine, Farmington, CT USA; 4 Division of Molecular Pathology, Netherlands Cancer Institute, Amsterdam, The Netherlands; 5 Oncode Institute, Utrecht, The Netherlands; 6 Department of Radiation Oncology, Rutgers Cancer Institute, New Brunswick, NJ USA; 7 The Jackson Laboratory, Bar Harbor, ME USA; 8 Present Address: Department of Cancer Biology, Perelman School of Medicine, University of Pennsylvania, Philadelphia, PA, USA; 9 Present Address: Tasca Therapeutics, Cambridge, MA USA

**Keywords:** Cancer, DNA Replication, Recombination & Repair

## Abstract

*BRCA1*-linked cancers contain abundant ~10 kb ‘Group 1’ tandem duplications (TDs). Group 1 TDs form at a Tus/*Ter* replication-fork barrier in DNA-end resection-defective mouse embryonic stem (mES) cells lacking *Brca1* exon 11. To elucidate how BRCA1 suppresses Group 1 TDs, we analyzed *Brca1* coiled-coil (CC)-domain mutants—separation-of-function alleles impaired for homologous recombination (HR) through loss of PALB2-binding and RAD51-loading functions but competent for DNA-end resection. Notably, *Brca1* CC mutants retain the ability to suppress Group 1 TDs in the Tus/*Ter* system and in a mouse model of *Brca1*-linked mammary tumorigenesis. These data suggest that *Brca1* CC domain-mutant cancers follow a path of tumorigenesis distinct from that of other *Brca1*-linked cancers. FANCM is a TD co-suppressor, loss of which is synthetic lethal/sick in *Brca1* exon 11-deleted cells. In contrast, *Fancm* deletion unexpectedly improves the growth of *Brca1* CC mutant and *Brca2* mutant mES cells. Thus, Group 1 tandem duplication formation and *Fancm* synthetic lethality are linked phenotypes, potentially related to defective BRCA1-mediated DNA end resection but genetically separable from BRCA1/PALB2-mediated RAD51 loading.

## Synopsis

*BRCA1* mutants accumulate abundant ~10 kb tandem duplications (TDs) and are synthetic lethal with *FANCM* loss. Despite their homologous recombination defect, *Brca1* coiled-coil domain mutants are competent for TD suppression and tolerate *Fancm* deletion.

- *Brca1* coiled-coil (CC)-domain mutants are defective for homologous recombination (HR) at stalled replication forks and in double-strand break (DSB) repair.
- *Brca1* CC-domain mutants are competent for DNA-end resection during DSB repair, suppress ~10 kb (‘Group 1’) TDs at a Tus/Ter replication-fork barrier and during tumorigenesis, and tolerate *Fancm* loss.
- Group 1 TD formation and *Fancm* synthetic lethality appear to be linked phenotypes, potentially related to defective BRCA1-mediated DNA end resection but genetically separable from overall HR function.
- The data suggest that *Brca1* CC-domain mutant cancers follow a path of tumorigenesis distinct from that of other *Brca1*-linked cancers.

## Abstract (web summary)

*Brca1* CC-domain mutant cancers appear to follow a path of tumorigenesis distinct from that of other *Brca1*-linked cancers.

## Graphical abstract

**Figure:**
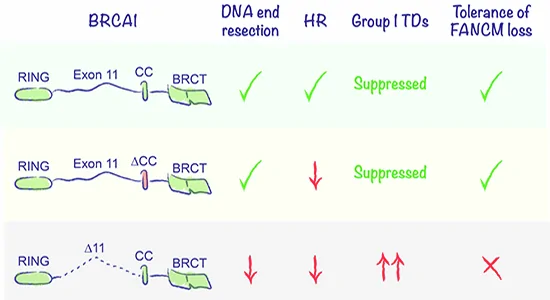

## Introduction

The hereditary breast/ovarian cancer (HBOC) predisposition genes *BRCA1* and *BRCA2* regulate homologous recombination (HR), a DNA repair pathway involved in the error-free repair of double-strand breaks (DSBs), protection of replication forks, and the prevention of genomic instability (Chen et al, 2018; Scully et al, 2019). Several features of *BRCA1*- and *BRCA2*-linked cancers are shared, reflecting their roles as key HR genes. These features include elevated levels of genomic instability, a specific mutational signature (SBS3), and sensitivity to inhibitors of poly(ADP-ribose) polymerase (PARP) (Lord and Ashworth, 2017; Nik**-**Zainal et al, 2016). However, the genomes of *BRCA1*-linked breast and ovarian cancers uniquely reveal abundant ~10 kb (“Group 1”) non-homologous tandem duplications (TDs), distributed widely across the genome (Menghi et al, 2016; Nik**-**Zainal et al, 2016; Willis et al, 2017). “Non-homologous” here refers to the absence of sequence homology between the two TD breakpoints, which define the edges of the duplicated segment and are apposed to form the non-homologous TD breakpoint junction (Scully et al, 2025). *BRCA1*-linked Group 1 TDs differ from the more common types of cancer-related TDs, which are typically >100 kb in size. Strong (~ 95%) mutual concordance between *BRCA1* loss and Group 1 ‘TD phenotype’ (TDP) in human breast and ovarian cancer suggests a causal relationship between *BRCA1* loss and Group 1 TD formation (Willis et al, 2017). Indeed, conditional inactivation of *Brca1* in the mouse mammary epithelium recapitulates the Group 1 TD phenotype (Menghi et al, 2018). Loss of *BRCA2* is not associated with Group 1 TDP in either human or mouse tumorigenesis, nor is *Brca2* required for the formation of the Group 1 TD in the mouse mammary tumor model. These observations raise several questions: what mechanisms normally suppress Group 1 TDs, and what functions specific to BRCA1 mediate Group 1 TD suppression?

BRCA1 and BRCA2 operate at distinct steps of the HR pathway (Chen et al, 2018; Scully et al, 2019; Zhao et al, 2019). The BRCA1-BARD1 heterodimer promotes short-range DNA end resection by MRE11/RAD50/NBS1 through interactions with the scaffolding protein, CtIP (Cejka and Symington, 2021; Cruz**-**Garcia et al, 2014; Yu et al, 1998). BRCA1-BARD1 also activates long-range resection mediated by EXO1 or by the Bloom’s (BLM) or Werner’s (WRN) syndrome helicases in association with DNA2 (Ceppi et al, 2024; Salunkhe et al, 2024). These resection activities convert the blunt or near-blunt ends of a DSB to single-stranded (ss)DNA with long 3’ tails, which are then rapidly bound by the ssDNA binding protein complex RPA. BRCA2 displaces RPA from ssDNA and loads RAD51 in its place, forming the RAD51 nucleoprotein filament, which then executes the search for a homologous template (Bell et al, 2023; Jensen et al, 2010; Shahid et al, 2014; Thorslund et al, 2010). In addition to its role in DNA end resection, BRCA1-BARD1 interacts with the product of another HBOC gene, *PALB2*, facilitating BRCA2 recruitment and RAD51 loading (Anantha et al, 2017; Dray et al, 2010; Sy et al, 2009; Xia et al, 2006; Zhang et al, 2009). BRCA1 also acts at later stages of HR, facilitating strand exchange with a homologous template by the RAD51 nucleoprotein filament (Zhao et al, 2017). BRCA1 binds PALB2 directly *via* its coiled coil (CC) domain, mutations in which disrupt the interaction (Sy et al, 2009; Zhang et al, 2009). Consequently, BRCA1 CC domain mutants are defective for HR but competent for DNA end resection. Human germline *BRCA1* CC missense alleles are exceptionally rare, and these alleles are classified as variants of uncertain significance (VUS). However, a coiled coil missense mutation in the mouse *Brca1* CC domain, p.L1363P, was found to promote mouse mammary tumorigenesis, suggesting that its human counterpart p.L1407P, a patient-derived variant, is pathogenic (Pulver et al, 2021). Consistent with the separation-of-function properties of BRCA1 CC domain mutants, mice that are compound heterozygotes of *Brca1* ∆11 and *Brca1* ∆CC display interallelic complementation in mouse development (Nacson et al, 2020). BRCA1’s function in DNA end resection may explain its role in single-strand annealing (SSA)—a BRCA2/RAD51-independent DSB repair mechanism whereby two neighboring homologous repeats, either side of a DSB, can be collapsed to one copy by annealing of the complementary sequences exposed by DNA end resection (Chen et al, 2018; Scully et al, 2019).

To study mechanisms of stalled fork repair in mammalian cells, we previously adapted the *Escherichia coli* Tus/*Ter* replication fork barrier (RFB) to provoke bidirectional site-specific replication fork stalling on a mammalian chromosome (Willis et al, 2014). By targeting an HR reporter containing an array of six 23 bp *Ter* sequences to a defined chromosomal locus, we were able to quantify HR and other repair outcomes triggered by fork stalling at the Tus/*Ter* RFB. We found that conservative HR by short tract gene conversion (STGC) at Tus/*Ter* is mediated by the canonical BRCA1-BRCA2-RAD51 pathway, whereas replicative long tract gene conversion (LTGC) at Tus/*Ter* is independent of BRCA1 and BRCA2 (Willis et al, 2014). In addition, non-homologous Group 1 TDs form at Tus/*Ter* in *Brca1* mutant mouse embryonic stem (mES) cells, but not in *Brca2* mutants (Willis et al, 2017). This pattern of genetic dependency for TD formation suggests that the Tus/*Ter* system recapitulates the *BRCA1*-linked Group 1 TD mechanism observed in cancer.

Analysis of Tus/*Ter*-induced Group 1 TD formation in mES cells identified BRCA1, BARD1, and CtIP as major TD suppressors in wild-type (WT) cells (Willis et al, 2017). The CtIP connection suggested a possible role for DNA end resection in TD suppression. Notably, Tus/*Ter*-induced TDs in DNA end resection-defective *Brca1* mutants lacking the large central in-frame exon 11 (*Brca1*^∆11^) are elevated ~15-fold by loss of either of two stalled fork motor proteins, FANCM or BLM, whereas loss of FANCM or BLM has minimal impact on TD formation in cells expressing WT *Brca1* (Panday et al, 2021; Willis et al, 2017). FANCM and BLM act in concert as TD suppressors, and loss of *Fancm* is synthetic lethal/synthetic sick with *Brca1*^∆11^ mutation (Panday et al, 2021). Indeed, specific inactivation of FANCM ATPase function is sufficient to disable *Brca1*^∆11/–^ cell growth. Whole-genome CRISPR/Cas9 screens in primary human and mouse cells also identified specific synthetic lethality following combined loss of *BRCA1* and *FANCM*, but not in cells lacking *BRCA2* and *FANCM* (Adam et al, 2021; preprint: Dossin et al, 2026). These findings, if verified in cancer models, identify FANCM as a potential target for therapy in *BRCA1*-linked cancer.

We proposed that TDs form by a replication restart/replication bypass mechanism, in which FANCM/BLM suppresses fork restart at the Tus/*Ter* RFB, while BRCA1 promotes SSA-mediated collapse of a nascent TD back to single copy (Scully et al, 2025; Scully et al, 2019). Such a mechanism could explain how the combined inactivation of FANCM/BLM and BRCA1-mediated DNA end resection disables different steps of the TD suppression mechanism and thereby synergizes to induce very high levels of TDs and, potentially, other genomic instability outcomes (Panday et al, 2021; Setton et al, 2023). However, the explicit relationships between BRCA1-mediated DNA end resection, Group 1 TD formation, and *FANCM* synthetic lethality remain to be rigorously tested. Here, we study these relationships by analyzing the stalled fork repair functions of *Brca1* CC domain mutants—separation-of-function mutants that disrupt HR without affecting BRCA1-mediated DNA end resection. Our findings suggest that Group 1 TD formation and *Fancm* synthetic lethality are linked phenotypes associated with defective BRCA1-mediated DNA end resection but are genetically separable from BRCA1/PALB2-mediated RAD51 loading functions.

## Results

### A haploid system for genetic analysis of *Brca1* in stalled fork repair

**Figure EV1:**
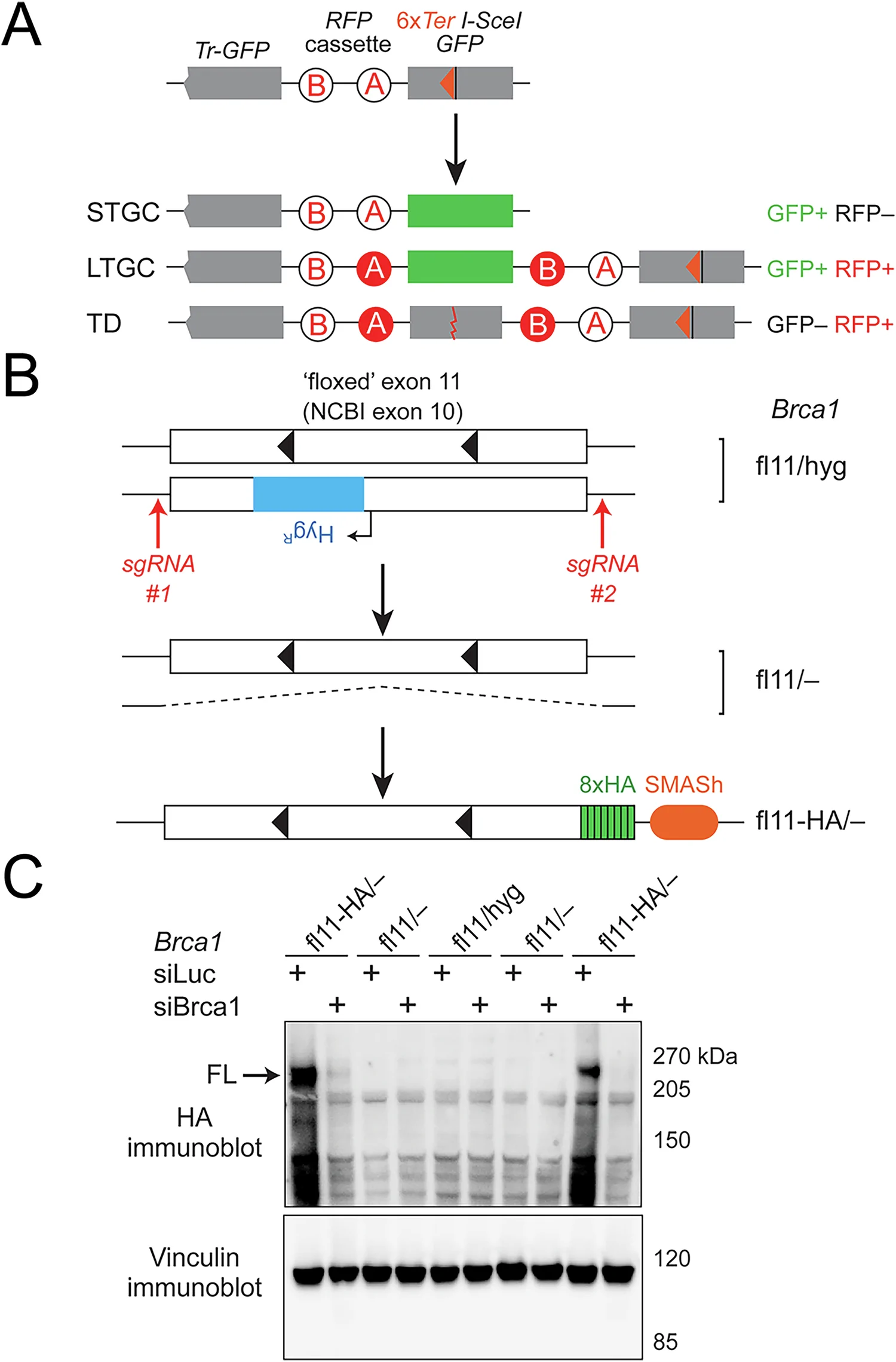
A haploid system for genetic analysis of *Brca1* in stalled fork repair. (**A**) Schematic of *Ter*-HR reporter for quantitation of Tus/*Ter*-induced and DSB-induced HR. *Tr-GFP*: 5’ truncated *GFP* allele acts as a donor during HR. *RFP* cassette elements A and B: artificial exons encoding WT *RFP*, placed in unproductive orientation. Promoters and polyadenylation signals not shown. STGC short tract gene conversion, LTGC long tract gene conversion, TD Group 1 tandem duplication. Each repair product is associated with fluorescent products, as shown. Red line in TD product: non-homologous TD breakpoint junction. (**B**) Outline of method for generating haploid mES cells expressing *Brca1*-HA. Black triangles: *LoxP* sites flanking *Brca1* exon 11 (NCBI exon 10). The deleted *Brca1* allele is not shown in the cartoon of *Brca1*^fl11-HA/–^ allele. (**C**) Identification of HA-tagged gene product in *Brca1*^fl11-HA/–^ cells. Upper panel: anti-HA immunoblotting of whole cell extracts. Note the abolition of the BRCA1-HA signal by *Brca1* siRNA but not by control *Luciferase* siRNA (siLuc). FL full-length *Brca1* gene product. Lower panel: Vinculin loading control.

To support a genetic analysis of *Brca1* in stalled fork repair, we generated a system that enables targeted mutagenesis of *Brca1* as a haploid genetic element in mES cells. This approach guarantees that subsequent CRISPR/Cas9-mediated mutagenesis of *Brca1* cannot induce unplanned changes in a second *Brca1* allele that might produce unpredictable alterations in *Brca1* function (Egli et al, 2018; Panday et al, 2021). We first determined whether *Brca1*^+/–^ mES cells display an overt haploinsufficiency phenotype in DSB repair or stalled fork repair. We targeted a previously described *Ter*-HR reporter (Willis et al, 2014) to the *Rosa26* locus of *Brca1*^fl5-13/+^ cells, in which *Brca1* exons 5–13 are flanked by *loxP* sites (Fig. EV1A; Appendix Fig. S1A) (Liu et al, 2007). The *Brca1*^∆5-13^ allele is functionally null. We then generated Cre-treated derivatives in which *Brca1* is either haploid (*Brca1*^∆5-13/+^) or diploid (*Brca1*^fl5-13/+^) (Appendix Fig. S1B). In assays of DSB-induced HR or Tus/*Ter*-induced HR, *Brca1*^fl5-13/+^ and *Brca1*^∆5-13/+^ HR reporter cells were indistinguishable (Appendix Fig. S1C,D). Thus, *Brca1* is not overtly haploinsufficient for DSB-induced or stalled fork HR in mES cells. This conclusion is restricted to the cells, DNA repair functions, and genotypes studied here.

We next engineered haploid derivatives of *Ter*-HR reporter mES cells that carry one conditional allele of *Brca1*, *Brca1*^fl11^, in which the large central in-frame exon 11, corresponding to NCBI exon 10 (RefSeq NM_009764), can be conditionally deleted using Cre-mediated recombination (Xu et al, 1999). The Cre-deleted allele, *Brca1*^∆11^, is a viable hypomorph that is defective for DNA end resection (Bunting et al, 2010). The second allele, *Brca1*^hyg^, contains a hygromycin resistance gene that disrupts *Brca1* gene expression (Fig. EV1B). To delete the *Brca1*^hyg^ allele, we used two sgRNAs to target Cas9 to the extreme 5’ and 3’ ends of the *Brca1* open reading frame (ORF) and screened for clones in which one full allele had been deleted (Fig. EV1B). All derived clones were *Brca1*^fl11/–^, likely reflecting the inviability of *Brca1*^hyg/–^ cells. We selected a *Brca1*^fl11/–^ clone that contained no indels in the retained *Brca1*^fl11^ allele at the 5’ and 3’ sgRNA target sites. We next used CRISPR/Cas9-assisted gene targeting to introduce an in-frame linker peptide, followed by an 8x influenza hemagglutinin (HA) epitope tag sequence to the 3’ end of the *Brca1* ORF, together with a SMASh degron (Chung et al, 2015) and a neomycin expression cassette fused to a self-cleaving peptide T2A, to generate *Brca1*^fl11-HA/–^ cells (Fig. EV1B). The SMASh degron is also self-cleaving so that, in the absence of the degron-activating drug asuprenavir, the C-terminus of BRCA1 is fused to only the linker peptide and the 8xHA epitope tag. The full-length ~220 kDa gene product, BRCA1-8xHA, was detectable by western blotting and was abolished by siRNA-mediated depletion of *Brca1* mRNA (Fig. EV1C). Anti-HA immunoblotting also revealed lower molecular weight *Brca1* products, reflecting products of alternative splicing or proteolytic cleavage of full-length BRCA1. We tested the HR functions of parental *Brca1*^fl11/hyg^, *Brca1*^fl11/–^, and *Brca1*^fl11-HA/–^ cells and found them to be equivalent (Appendix Fig. S1E).

### Generation of *Brca1* coiled coil domain mutants

**Figure 1:**
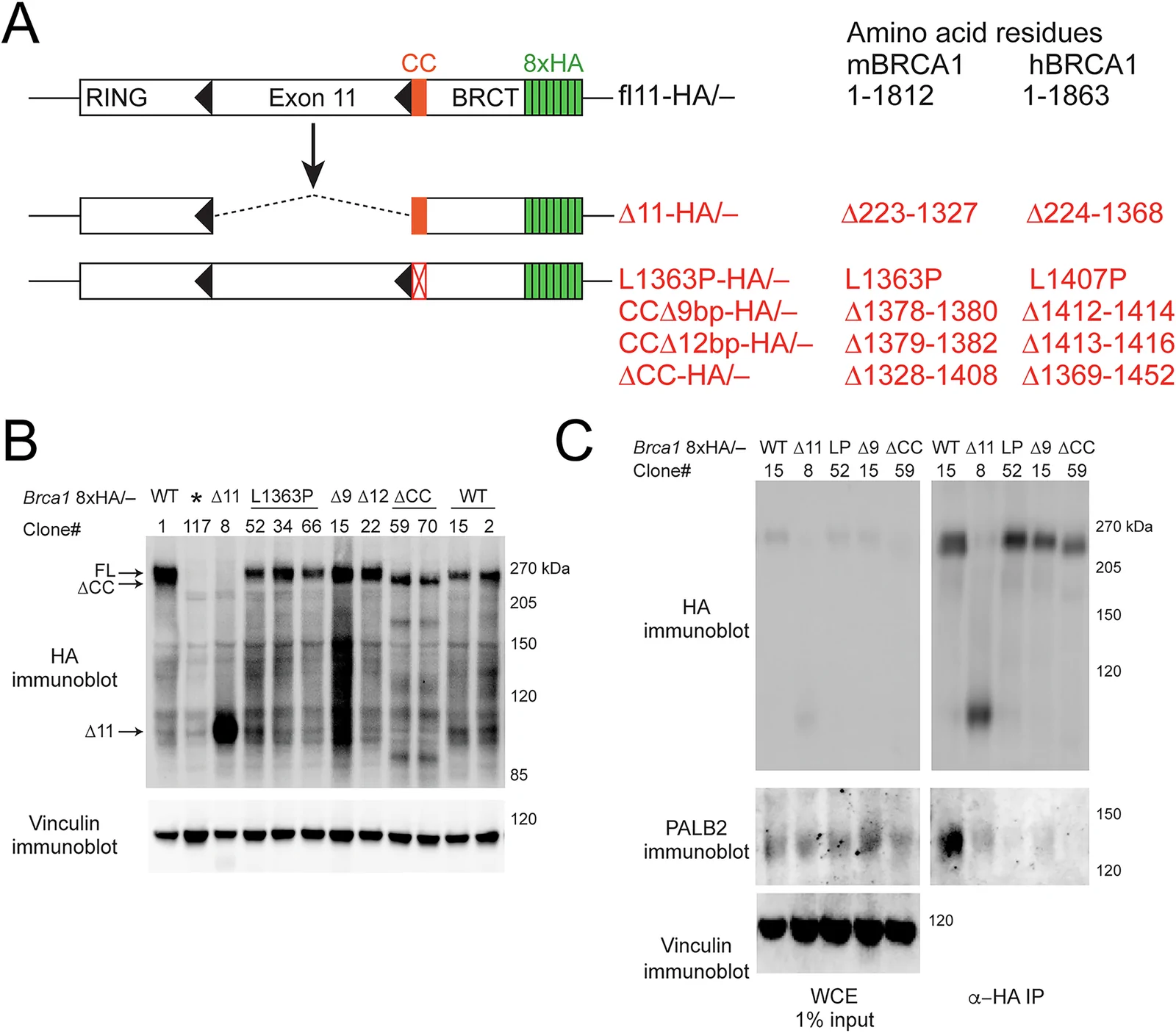
Generation of *Brca1* coiled coil domain mutants. (**A**) Schematic of *Brca1* mutant alleles generated by Cre-mediated recombination (*Brca1*^∆11-HA/–^) or by CRISPR/Cas9-mediated gene editing. *Brca1* allele names are shown, together with abbreviations used in this and subsequent figures and corresponding amino acid residues in mouse and human BRCA1. (**B**) Identification of HA-tagged gene product in *Brca1*^HA/–^ cells with mutations as shown. *Parental *Brca1*^fl11/–^ clone, lacking HA-tag. Upper panel: anti-HA immunoblotting of whole cell extracts. FL: arrow shows full-length BRCA1. ∆CC: arrow shows *Brca1*^∆CC-HA/–^ gene product. Lower panel: vinculin loading control. (**C**) Detection of PALB2 association with products of different *Brca1*^HA/–^ alleles. Left panels: whole cell extracts. Right panels: anti-HA immunoprecipitates. Immunoblots were probed with the antibodies shown. Lower panel: Vinculin loading control in whole cell extracts. Source data are available online for this figure.

**Figure EV2:**
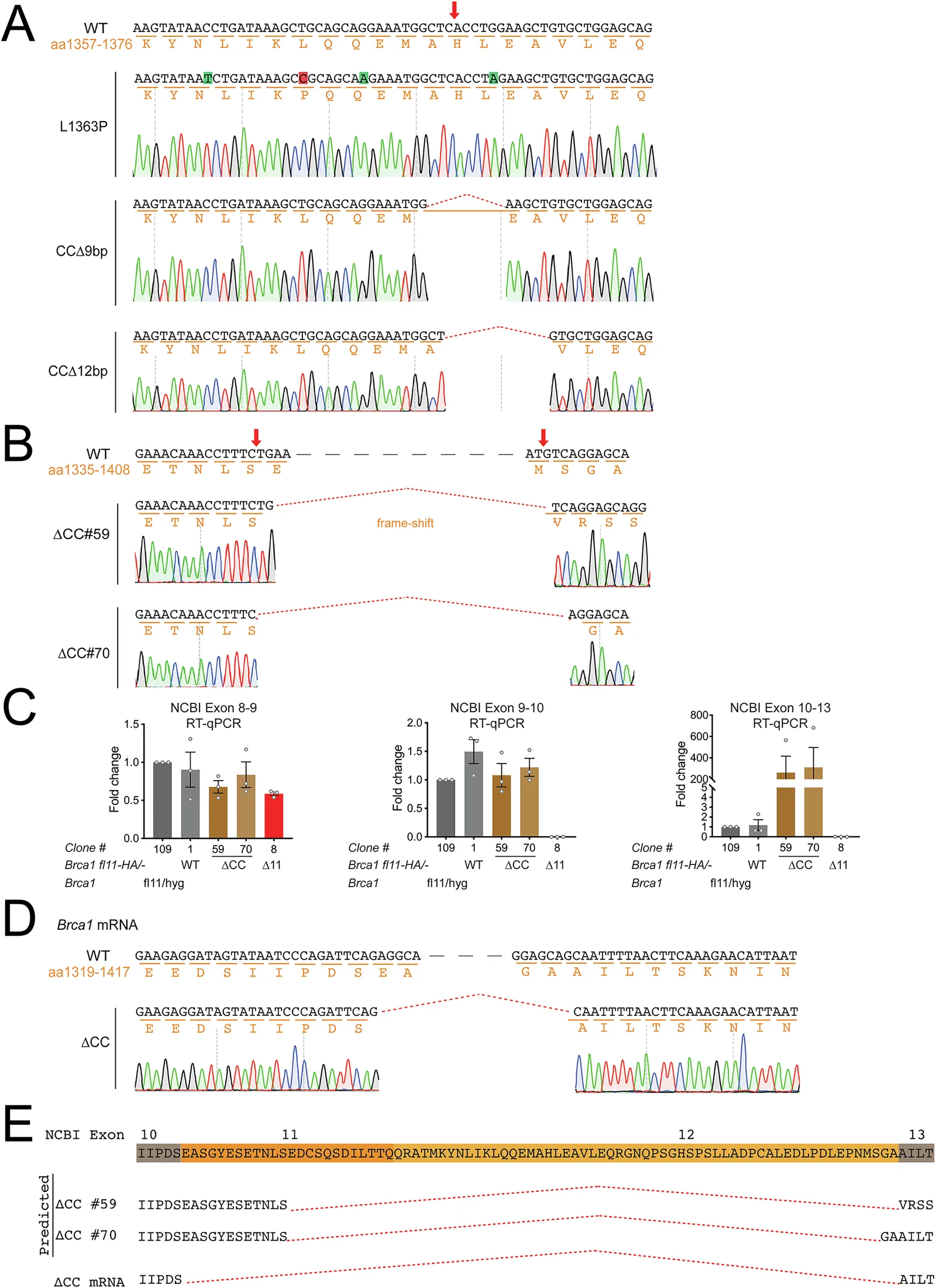
Sequence analysis of *Brca1* CC mutant clones and exon skipping in *Brca1*^∆CC-HA/–^ cells. *Brca1* allele abbreviations as in Fig. 1A. Nucleotide sequence is shown in black and amino acids in orange. (**A**) Nucleotide sequence of mouse *Brca1* NCBI exon 12 encoding amino acids 1357-1376 (equivalent residues in human BRCA1: 1401-1420). The red arrow shows the predicted site of Cas9-induced DSB for the generation of CC mutants. The red dotted line denotes deletion of nucleotides. Sequences of *Brca1* p.L1363P, *Brca1* CC∆9 bp, and *Brca1* CC∆12 bp alleles are shown. L to P mutation in *Brca1* p.L1363P mutants is highlighted in red. Engineered silent mutations in potential sgRNA PAM sites are highlighted in green. (**B**) *Brca1* NCBI exon 11 and 12 nucleotide sequence encoding amino acids 1335 to 1408. ∆CC clone #59 has a frame-shift mutation, whereas clone #70 is in-frame. (**C**) RT-qPCR results showing amplification across three different exon-exon junctions (normalized to parental *Brca1*^fl11/hyg^ clone #109). Note the absence of NCBI exons 10–11 amplification in ∆11 mutant clones and over-representation of NCBI exon 10–13 RT-qPCR product in ∆CC clones, reflecting exon skipping. Data show mean and s.e.m. of three independent biological repeats (*n* = 3). (**D**) Sequence of WT *Brca1* cDNA and *Brca1* ∆CC cDNA encompassing NCBI exons 10–13. The black dashed line represents the continuation of the sequence. The red dotted line represents the deletion of nucleotides. ∆CC clones #59 and #70 revealed identical cDNA sequences, consistent with skipping of NCBI exons 11 and 12 in the mRNA and cDNA. (**E**) BRCA1 amino acid sequence region encoded by the 3’ end of *Brca1* NCBI exon 10 to the 5’ end of NCBI exon 13. Amino acids encoded by NCBI exons 10 and 13 are highlighted in gray. Amino acids encoded by NCBI exons 11 and 12 are highlighted in orange and yellow, respectively. Predicted amino acid sequence based on sequencing of *Brca1* gDNA is shown for each ∆CC clone. Actual amino acid sequence based on *Brca1* ∆CC cDNA sequencing shows in-frame deletion resulting from skipping of NCBI exons 11–12.

To engineer *Brca1* CC domain mutants, we used CRISPR/Cas9-assisted gene targeting to introduce the missense mutation *Brca1* p.L1363P into *Brca1*^fl11-HA/–^ cells (See Methods). This allele is equivalent to the human *BRCA1* p.L1407P missense variant, which shows impaired PALB2 binding (Sy et al, 2009). Because it is a rare allele in the human population, *BRCA1* p.L1407P is classified in ClinVar as a variant of uncertain significance (VUS). However, *Brca1* p.L1363P promotes tumorigenesis in a mouse model of mammary carcinoma (Pulver et al, 2021). We identified several independent *Brca1*^L1363P-HA/–^ clones that express the gene product at levels equivalent to WT BRCA1, as well as two non-targeted clones in which small, in-frame deletions of 9 or 12 bp had been introduced at the sgRNA target site within the CC-coding region (Figs. 1A,B and  EV2A). These deletion alleles were termed *Brca1*^CC∆9bp-HA/–^ (abbreviated to ‘∆9 bp’ in some figures) and *Brca1*^CC∆12bp-HA/–^, respectively.

We also used CRISPR/Cas9 dual incisions to generate larger deletions within the *Brca1* CC-encoding region (*Brca1*^∆CC-HA/–^), spanning the two exons (NCBI exons 11 and 12) that encode this domain. We retrieved two clones, one of which (#70) contained a predicted in-frame deletion and one (#59) a frame-shifting deletion (Fig. EV2B). Despite these genetic differences, the two alleles encoded HA-tagged *Brca1* gene products of equal size that were slightly smaller than the WT BRCA1 protein (Fig. 1B). Analysis of *Brca1* mRNA by RT-qPCR across specific exon-exon junctions and by direct sequencing of *Brca1* cDNA revealed that NCBI exons 11 and 12 were skipped in both ∆CC clones, resulting in an in-frame deletion of 81 amino acids, effectively deleting the entire coiled coil domain (Fig. EV2C–E). This exon-skipping explains why ∆CC allele #59 does not truncate the protein, and why the two different ∆CC alleles encode similar gene products. We presume that the emergence of the exon-skipping adaptation is achieved through selection against cells that fail to skip NCBI exons 11 and 12, since the exon-skipped mRNA is virtually undetectable in WT cells (Fig. EV2C). It is not clear how reconfigurations of the splicing apparatus enable the observed exon skipping to occur.

We used Cre-mediated recombination to delete exon 11 in *Brca1*^fl11-HA/–^ cells (see “Methods”) and identified the *Brca1*^∆11-HA/–^ gene product by western blotting (Fig. 1A,B). We used immunostaining and immunofluorescence microscopy to assess the subcellular localization of each *Brca1* gene product (Appendix Fig. S2). Consistent with previous reports, BRCA1∆11-HA and all other BRCA1 proteins localized to characteristic nuclear foci (Huber et al, 2001; Scully et al, 1996). The anti-HA signal of WT and CC mutant proteins co-localized with foci detected by an antibody raised against a polypeptide encoded by *Brca1* exon 11, whereas this exon 11-specific BRCA1 signal was absent in *Brca1*^∆11-HA/–^ cells (Appendix Fig. S2). The anti-HA signal was absent in *Brca1*^fl11/–^ cells, which lack the HA epitope tag. The mean numbers of HA-positive foci per cell were similar across all *Brca1*^HA/–^ genotypes (Appendix Fig. S3A).

We next assessed PALB2 binding by BRCA1 in each genetic background, using anti-HA immunoprecipitation (IP) followed by immunoblotting for endogenous PALB2 (Fig. 1C). Despite equivalent levels of PALB2 in cell extracts, IPs from BRCA1 CC mutants revealed a severely attenuated PALB2 signal, with BRCA1∆CC-HA revealing the most severe PALB2 binding defect. Interestingly, BRCA1∆11-HA also revealed reduced binding to PALB2 in comparison to WT BRCA1-HA, as was noted previously (Nacson et al, 2020). These results confirm that the *Brca1* CC mutants generated here are defective for PALB2 interaction and, hence, lack the contribution to RAD51 loading that this interaction normally contributes to the HR process.

### Defective HR and intact DNA end resection in *Brca1* coiled coil mutants

**Figure 2:**
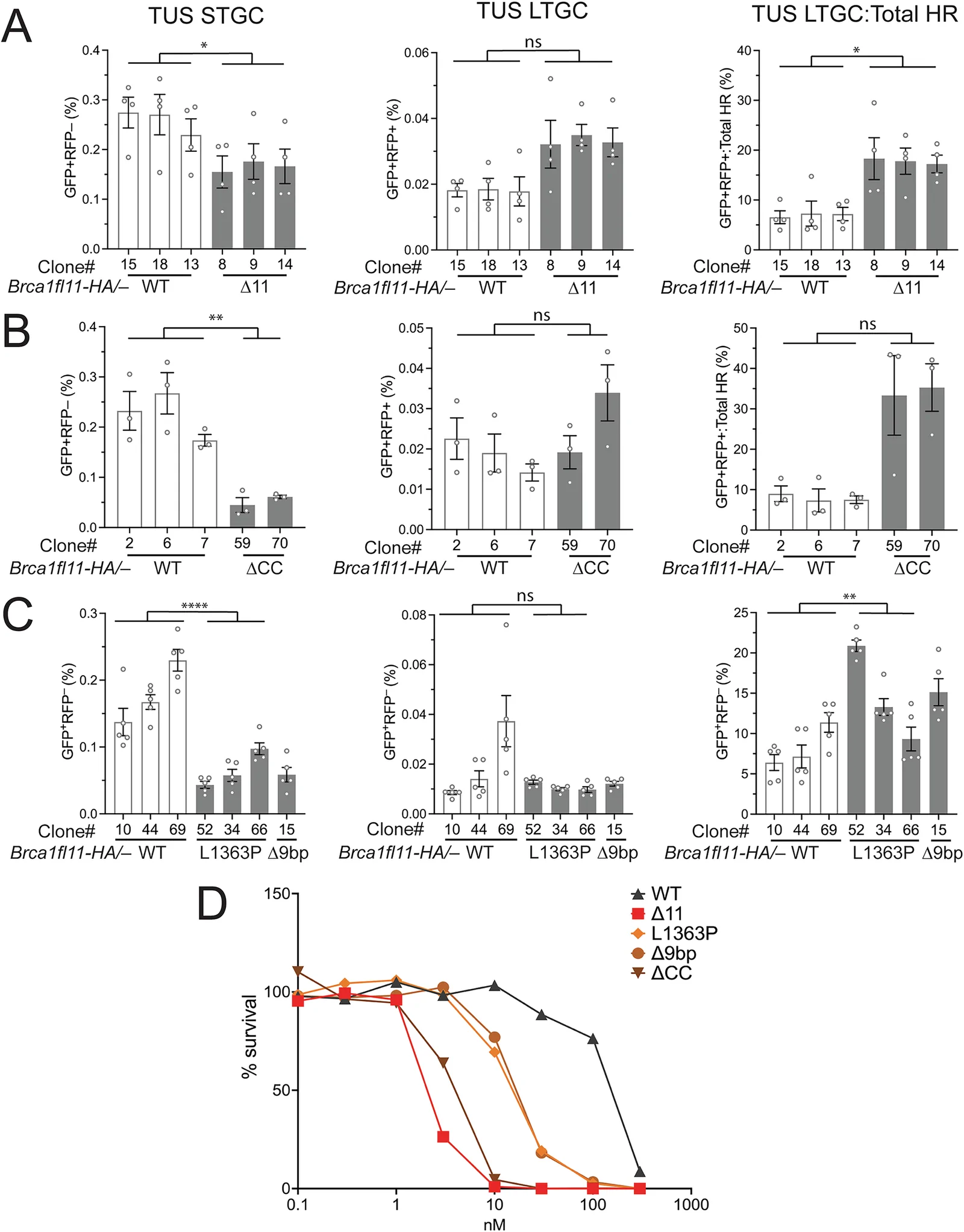
Tus/*Ter*-induced HR and PARPi sensitivity of *Brca1* coiled coil mutants. HR was quantified in independent *Brca1* mutant (gray) or isogenic *Brca1* WT (white) clones as described in “Methods”. *Brca1* allele abbreviations as in Fig. 1A. Data show mean and s.e.m. of n independent experiments. (**A**) *Brca1*^∆11-HA/–^ (*n* = 4). (*P* values: STGC 0.0469; LTGC 0.0702; LTGC:Total HR 0.0120). (**B**) *Brca1*^∆CC-HA/–^ cells (*n* = 3). (*P* values: STGC 0.0080; LTGC 0.2764; LTGC:Total HR 0.0935). (**C**) *Brca1*^L1363P-HA/–^ and *Brca1*^CC∆9bp-HA/–^ (*n* = 5). (*P* values: STGC < 0.0001; LTGC 0.0551, LTGC:Total HR 0.0023). Statistical analysis: RM-one way ANOVA with Geisser–Greenhouse correction: ns: not significant, *P < 0.05, **P < 0.005, ***P < 0.0005, ****P < 0.0001. (**D**) Colony formation of different *Brca1*^HA/–^ genotypes in response to concentrations of Olaparib as shown (see “Methods”). Data show the mean of five independent biological replicates normalized to colony numbers in untreated samples of the same genotype (*n* = 5). Source data are available online for this figure.

**Figure EV3:**
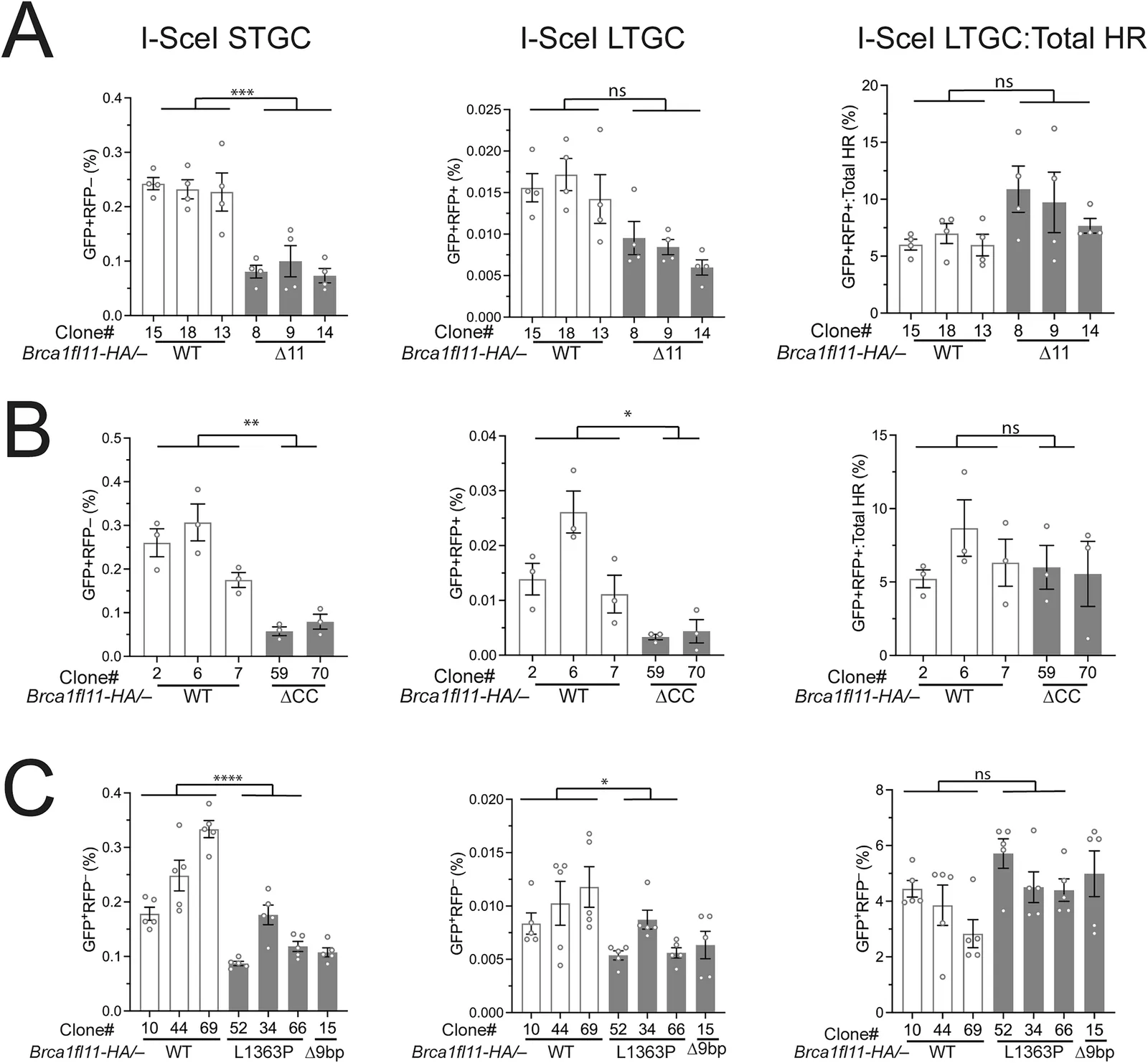
DSB-induced HR defects in *Brca1* mutants. (**A**, **C**) DSB-induced HR was quantified in *Brca1* mutants *vs*. isogenic *Brca1*^fl11-HA/–^ clones. *Brca1* allele abbreviations as in Fig. 1A. Data show mean and s.e.m. of *n* independent experiments. (**A**) *Brca1*^∆11-HA/–^ cells (*n* = 4). (*P* values: STGC 0.0004; LTGC 0.0635; LTGC:Total HR 0.1513). (**B**) *Brca1*^∆CC-HA/–^ cells (*n* = 3) (*P* values: STGC 0.0081; LTGC 0.0378; LTGC:Total HR 0.5448). (**C**) *Brca1*^L1363P-HA/–^ and *Brca1*^CC∆9bp-HA/–^ cells (*n* = 5). (*P* values: STGC < 0.0001; LTGC 0.0352; LTGC:Total HR 0.0802). Statistical analysis: RM-one way ANOVA with Geisser–Greenhouse correction: ns: not significant.

*Brca1* CC mutants are known to be defective for DSB-induced HR, but their function in stalled fork HR has not yet been studied. We showed previously that *Brca1*^∆11/hyg^ cells exhibit reduced STGC in response to a Tus/*Ter* RFB, although less markedly than in response to a replication-independent DSB (Willis et al, 2014). Tus/*Ter*-induced LTGC is independent of *Brca1* and *Brca2*. To study stalled fork HR in *Brca1* CC mutant cells, we quantified Tus/*Ter*-induced and I-SceI-induced HR in *Brca1*^HA/–^ mutant clones *vs*. isogenic WT controls, which had been exposed to the same CRISPR/Cas9 or Cre treatments but had retained WT *Brca1*. Mean values of Tus/*Ter*-induced STGC (averaged across the clones shown) were 64% of WT in *Brca1*^∆11-HA/–^ cells, 23% in *Brca1*^∆CC-HA/–^ cells, and 37% in *Brca1*^L1363P-HA/–^ cells (Fig. 2A–C). Equivalent mean values of I-SceI-induced STGC were 36% of WT in *Brca1*^∆11-HA/–^ cells, 27% in *Brca1*^∆CC-HA/–^ cells, and 50% in *Brca1*^L1363P-HA/–^ cells (Fig. EV3A–C). STGC defects in the *Brca1*^CC∆9bp-HA/–^ clone resembled those of *Brca1*^L1363P-HA/–^ clones. There was no consistent impact of *Brca1* CC domain mutation on Tus/*Ter*-induced LTGC (Fig. 2B,C). These results confirm our previous finding that defects in Tus/*Ter*-induced STGC in *Brca1* ∆11 cells and *Brca1* BRCT mutant cells are less pronounced than DSB-induced STGC (Willis et al, 2014). Interestingly, *Brca1* CC mutants behave differently, since they reveal approximately equivalent STGC defects in response to the two stimuli. Our findings suggest that the RAD51 loading function of BRCA1 (defective in *Brca1* ∆CC mutants) is equally important for DSB-induced and stalled fork HR, while the DNA end resection function of BRCA1 (defective in both *Brca1* exon 11 and BRCT mutants) plays a more important role in DSB-induced HR than in stalled fork HR.

To further characterize the HR functions of the different *Brca1* mutant clones, we assessed sensitivity to the PARP inhibitor Olaparib by colony formation assay (Fig. 2D). IC50 values were ~150 nM for WT cells, 2 nM for *Brca1*^∆11-HA/–^ cells, 3.5 nM for *Brca1*^∆CC-HA/–^ cells, and ~15 nM for both *Brca1*^L1363P-HA/–^ and *Brca1*^CC∆9bp-HA/–^ clones. Notably, *Brca1* ∆11 mutants were among the most sensitive to Olaparib and the most impaired in I-SceI-induced STGC, but revealed the mildest defect in Tus/*Ter*-induced STGC. This discordance suggests that an individual *Brca1* mutant’s PARPi sensitivity correlates better with its function in DSB-induced HR than in stalled fork HR.

**Figure 3:**
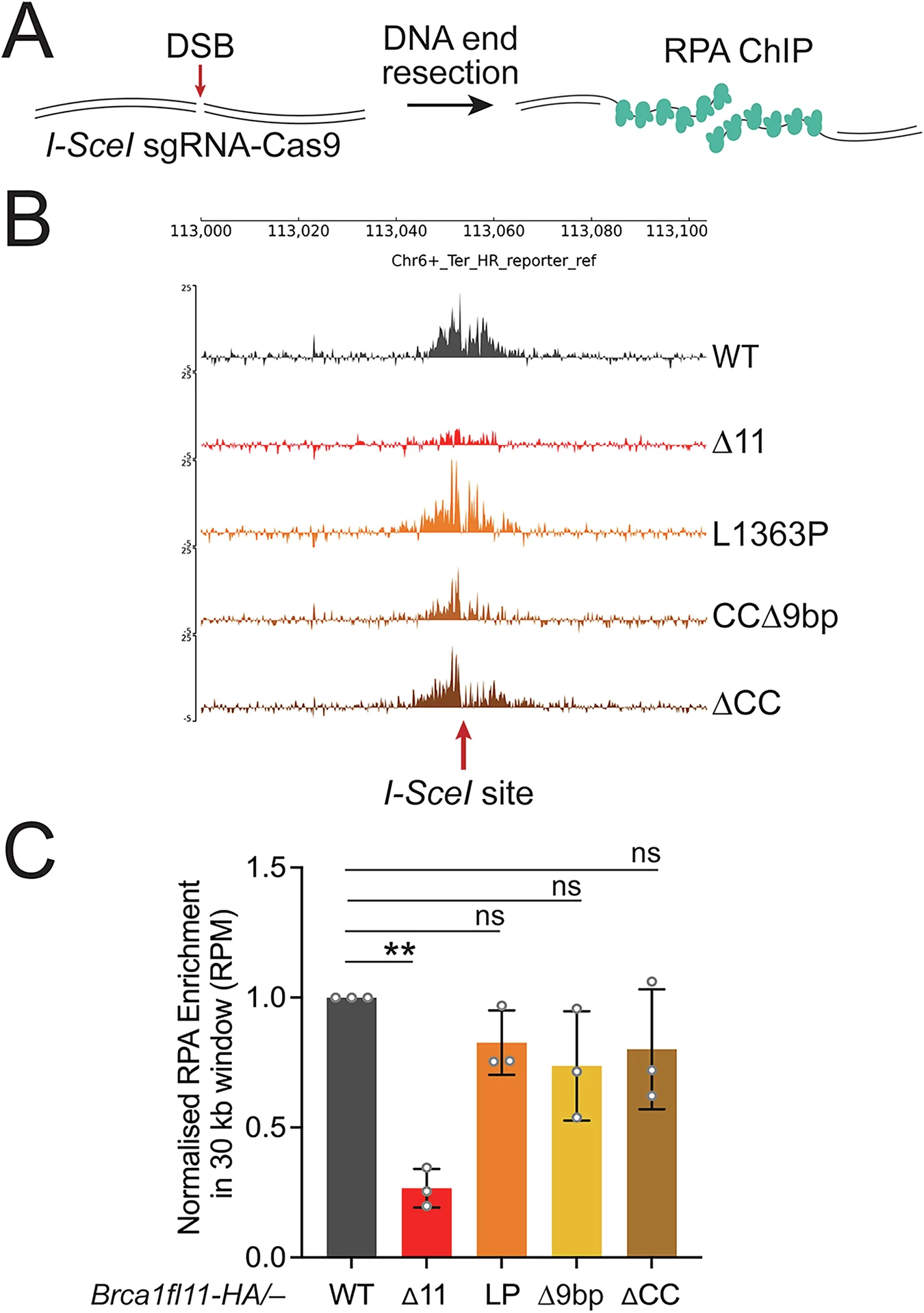
*Brca1* coiled coil mutants are competent for DNA end resection. (**A**) Schematic of RPA accumulation on ssDNA in response to DNA end resection of a Cas9-induced DSB, targeted to the *I-SceI* site within the HR reporter at *Rosa26*. Intensity of RPA ChIP-seq signal reflects the efficiency of DNA end resection. (**B**) Cas9 DSB-induced RPA ChIP-seq signals in one representative experiment of 3 independent biological replicates using *Brca1* genotypes shown. *Brca1* allele abbreviations as in Fig. 1A. See “Methods” for details. (**C**) Quantitation of RPA enrichment averaged over 30 kb window flanking *I-SceI* site from three independent experiments (*n* = 3; data show mean and S.D.). Signals were normalized to WT for each of the replicates. Statistical analysis: unpaired *t* test with Welch’s correction. ns not significant. (*P* values WT vs ∆11 0.0034; WT vs LP 0.1359; WT vs ∆9 bp 0.1626; WT vs ∆CC 0.2738). Source data are available online for this figure.

To determine the DNA end resection phenotypes of *Brca1* exon 11-deleted and CC domain mutants, we quantified RPA accumulation at a defined chromosomal DSB site using chromatin-immunoprecipitation-sequencing (ChIP-seq; see “Methods”) (Fig. 3A). Three hours after electroporation of CRISPR/Cas9 ribonucleoprotein (RNP) complexes targeting the *I-SceI* site within the *Ter*-HR reporter, we observed strong RPA enrichment in regions flanking the DSB site in *Brca1*^fl11-HA/–^ cells (Fig. 3B). In contrast, the RPA ChIP-seq signal was greatly diminished in *Brca1*^∆11-HA/–^ cells, consistent with the expected defect in DNA end resection. *Brca1* CC domain mutants *Brca1*^L1363P-HA/–^, *Brca1*^CC∆9bp-HA/–^ and *Brca1*^∆CC-HA/–^ each revealed levels of RPA accumulation similar to those of *Brca1*^fl11-HA/–^ cells (Fig. 3B,C). Thus, each of the *Brca1* CC domain mutants studied here supports WT levels of DNA end resection.

### *Brca1* CC domain mutants are competent for suppression of Tus/*Ter*-induced TDs

**Figure 4:**
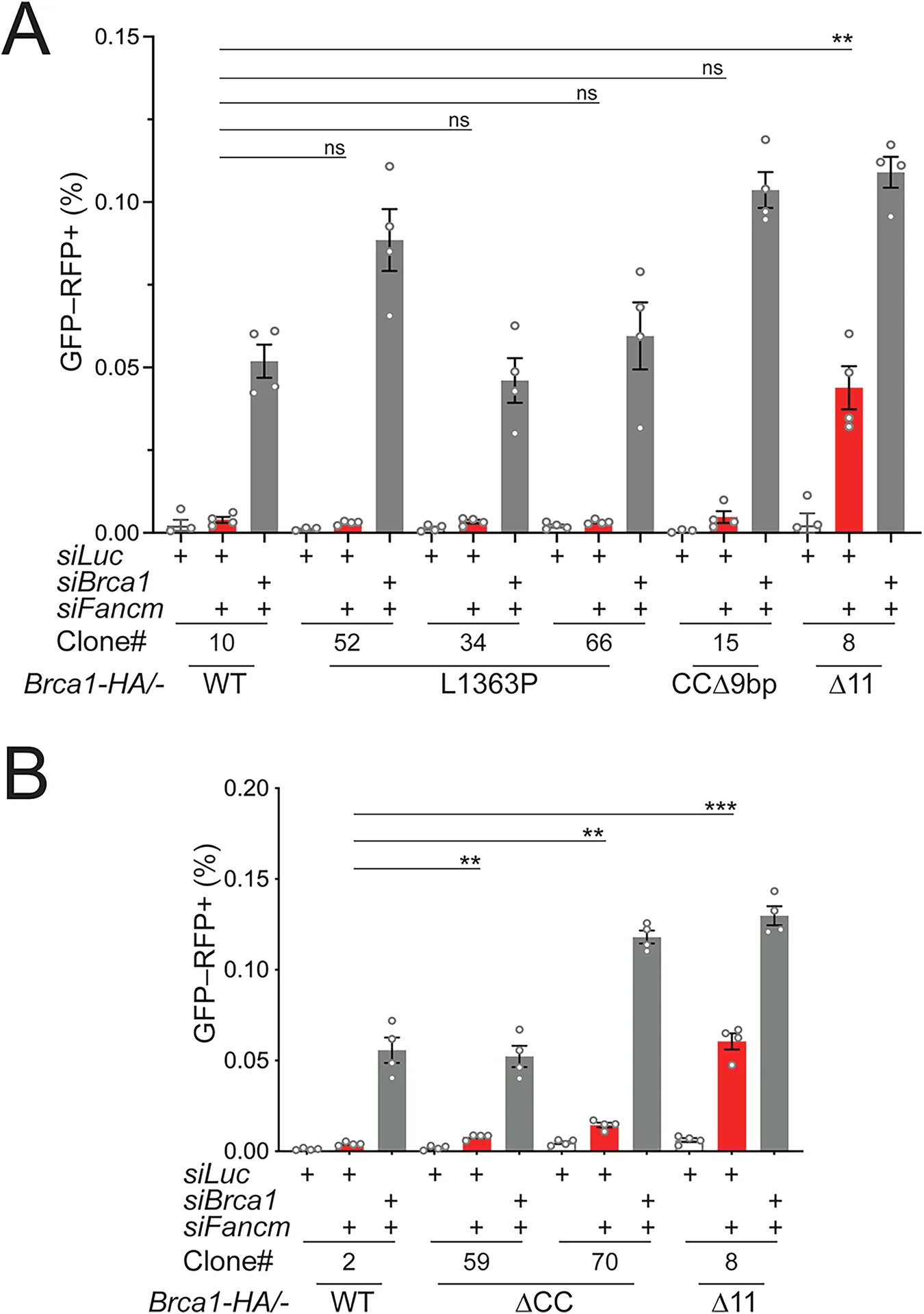
*Brca1* coiled coil mutants suppress Tus/*Ter*-induced tandem duplications. Tus/*Ter*-induced GFP^–^RFP^+^ repair products in *Brca1* WT and mutant genotypes, in the presence of siRNAs against *Fancm* and *Brca1*. *Brca1* allele abbreviations as in Fig. 1A. See “Methods” for details. siRNA against luciferase (si*Luc*): non-specific siRNA negative control. Gray columns: combined si*Fancm* and si*Brca1* induces TDs in all cell lines (positive control). Red columns: si*Fancm* and si*Luc* test TD suppression in the *Brca1* genotypes shown. (**A**) *Brca1*^L1363P-HA/–^ and *Brca1*^CC∆9bp-HA/–^ mutants are compared with isogenic WT *Brca1*^fl11-HA/–^ cells and with *Brca1*^∆11-HA/–^ cells (*n* = 4; data shows mean and s.e.m. of 4 independent biological repeats). Note induction of TDs in *Brca1*^∆11-HA/–^ cells but not in CC mutants in samples treated with *siFancm* alone. (**B**) *Brca1*^∆CC-HA/–^ mutants are compared with isogenic WT cells and *Brca1*^∆11-HA/–^ cells (*n* = 4; data show mean and s.e.m. of four independent biological repeats). Statistical analysis: Unpaired *t* test with Welch’s correction. ns not significant, **P < 0.005, ***P < 0.0005. Source data are available online for this figure.

We showed previously that FANCM depletion strongly enhances Tus/*Ter*-induced TD formation in *Brca1*^∆11/hyg^ cells (Panday et al, 2021; Willis et al, 2017). To test whether *Brca1* CC domain mutants are competent for TD suppression, we quantified Tus/*Ter*-induced TD frequencies in cultures of *Brca1*^fl11-HA/–^, *Brca1*^L1363P-HA/–^, *Brca1*^CC∆9bp-HA/–^, *Brca1*^∆CC-HA/–^, and *Brca1*^∆11-HA/–^ cells that had been depleted of FANCM using siRNA. Controls in this experiment included non-targeting luciferase siRNA (siLuc, negative control) and combined siRNA directed to *Brca1* and *Fancm* (positive control). The former provides the baseline level of Tus/*Ter*-induced TDs in each clone; the latter tests whether each clone can, in principle, form TDs when BRCA1 and FANCM are co-depleted. Indeed, each cell line revealed induction of Tus/*Ter*-induced GFP^–^RFP^+^ products (i.e., TDs) following siRNA-mediated co-depletion of BRCA1 and FANCM (Fig. 4; Appendix Fig. S3B,C). When depleted of FANCM alone, *Brca1*^fl11-HA/–^ cells revealed minimal TD induction, whereas *Brca1*^∆11-HA/–^ cells revealed high levels of Tus/*Ter*-induced TDs, as expected. Strikingly, unlike in *Brca1*^∆11-HA/–^ cells, depletion of FANCM alone failed to stimulate Tus/*Ter*-induced TDs in *Brca1*^L1363P-HA/–^ or *Brca1*^CC∆9bp-HA/–^ cells, with TD frequencies equivalent to those observed in WT *Brca1*^fl11-HA/–^ cells (Fig. 4A; Appendix Fig. S3C). *Brca1*^∆CC-HA/–^ cells revealed a modest increase in Tus/*Ter*-induced TDs, but significantly less than in *Brca1*^∆11-HA/–^ cells (Fig. 4B). Thus, *Brca1* CC domain mutants retain the ability to suppress Group 1 TD formation at a Tus/*Ter* RFB. We showed previously that I-SceI-induced GFP^–^RFP^+^ products are not non-homologous TDs, but represent a rare, aberrant outcome of sister chromatid recombination (Elango et al, 2025). *Fancm* depletion in the context of I-SceI-induced repair failed to generate a significant increase in GFP^–^RFP^+^ products in any of the *Brca1* mutant clones studied (Appendix Fig. S3D–F). In summary, the data show that the BRCA1 coiled coil domain and, hence, BRCA1/PALB2-mediated RAD51 loading is dispensable for Tus/*Ter*-induced Group 1 TD suppression. Since *Brca1* CC domain mutants are competent for DNA end resection, this result establishes a correlation (albeit a limited one, based on limited numbers of *Brca1* alleles tested) between BRCA1-mediated DNA end resection competence and Group 1 TD suppression at a Tus/*Ter* RFB.

### *Brca1* p.L1363P mouse mammary tumors do not display a Group 1 TD phenotype

Our findings that *Brca1* CC domain mutants are competent for suppression of Tus/*Ter*-induced TDs raised the question of whether cancers driven by *Brca1* CC mutation will accumulate Group 1 TDs genome-wide during tumorigenesis and whether the *Brca1* CC mutant cancer genome will register as Group 1 TDP. While the Tus/*Ter* system is an indication of TD formation at one specific euchromatic site, analysis of TDs in the *Brca1* CC mutant cancer genome would enable us to test how well the Tus/*Ter* system predicts Group 1 TD formation in cancer. We were unable to obtain tissue samples from human *BRCA1* CC mutant cancers, reflecting their rarity in the human population. We therefore analyzed a well-established mouse model of mammary tumorigenesis, in which *K14-Cre* or *WAP-Cre* expression in the mammary epithelium drives biallelic deletion of *Trp53* by converting *Trp53*^fl2-10/fl2-10^ to null (*Trp53*^∆2-10/∆2-10^) together with deletion of conditional alleles of *Brca1* (*Brca1*^fl5-13/fl5-13^) or *Brca2* (*Brca2*^fl11/fl11^) (Jonkers et al, 2001; Liu et al, 2007; Menghi et al, 2018). Pulver et al developed this model further, using the *Brca1* p.L1363P allele as a cancer driver (Pulver et al, 2021). This is the only tractable mouse model for studying the impact of *Brca1* CC mutation on mammary tumorigenesis. We analyzed TDs in the genomes of mammary tumors arising from *Cre*-expressing *Trp53*^fl2-10/fl2-10^ mice in three different *Brca* genotypes: first, *Brca1*^fl5-13/fl5-13^, in which Cre converts both *Trp53* and *Brca1* to null (*Brca1*^∆5-13/∆5-13^;*Trp53*^∆2-10/∆2-10^, hereafter termed ‘B1P’); second, *Brca2*^fl11/fl11^, in which Cre converts *Trp53* and *Brca2* to null (*Brca2*^∆11/∆11^;*Trp53*^∆2-10/∆2-10^, hereafter ‘B2P’); third, *Brca1*^fl5-13/L1363P^, in which Cre converts *Trp53* and the *Brca1*^fl5-13^ allele to null, resulting in functional expression of *Brca1*^L1363P^ alone (*Brca1*^∆5-13/L1363P^*;Trp53*^∆2-10/∆2-10^, hereafter ‘Br1L1363P’). We used whole-genome sequencing (WGS) to analyze 5 independent B1P and B2P tumors and 10 Br1L1363P tumors, using WGS of spleen tissue from each mouse to define the reference genome. For some of the analyses reported here, we included samples from our previous analysis of Cre-driven *Brca* mutant mouse mammary tumors, including controls in which *Trp53* was the only conditional allele (Menghi et al, 2018).

To confirm loss of the floxed regions *via* Cre-mediated recombination in each tumor genome, we first manually inspected the Cre-targeted regions using the Integrative Genomic Viewer (IGV), for evidence of (i) a decrease in coverage affecting the Cre-targeted segments of the Δ alleles of *Trp53*, *Brca1* or *Brca2* (ii) a deletion rearrangement supporting the recombination of targeted regions, and/or (iii) evidence of the p.L1363P mutation (examples in Appendix Fig. 4A).

Br1L1363P cancers contain a single copy of the *Brca1*^∆5-13^ allele in their genomes, and generally exhibit high stromal infiltration, as previously described (Pulver et al, 2021). Elevated stromal content can dilute tumor cell-derived genotype signals, making it challenging to determine the true extent of Cre-mediated recombination of floxed alleles in tumor cells (i.e., partial *vs*. complete). To address this potential confounder, we compared the ratio between the Δ allele frequency (DeltaFr) of the *Trp53* gene *vs*. that of the *Brca1* gene in both the Br1L1363P and B1P cancer genomes. A DeltaFr = 1 corresponds to 100% decrease in coverage across the Δ region compared to its flanking regions (i.e., no stromal contamination and complete recombination); whereas a DeltaFr = 0 corresponds to equal coverage between flanking and Δ regions (i.e., elevated stromal contamination and/or ineffective recombination). Finally, we computed the ratio between the DeltaFr relative to the *Trp53* and *Brca1* loci, working under the expectation that a complete recombination would result in *Trp53-DeltaFr:Brca1-DeltaFr* ratios of ~1 for the B1P models and ~2 for the Br1L1363P models (example in Appendix Fig. 4B; see also Methods). Indeed, this ratio was ~1 in B1P, but ~2 in Br1L1363P tumors, reflecting the loss of one *Brca1* allele and two *Trp53* alleles, with retention of the *Brca1* p.L1363P allele (Appendix Fig. 4C). In one Br1L1363P tumor (T7), loss of *Brca1*^fl5-13^ was accompanied by a much larger deletion that included the flanking sequences, making calculation of this ratio unreliable. This larger deletion resulted in the loss of the entire *Brca1*^fl5-13^ allele, as reflected by the p.L1363P mutation having a variant allele frequency of ~1 in this sample (not shown). In summary, this analysis showed that each tumor analyzed had undergone the planned cancer-promoting *Trp53*, *Brca1*, and/or *Brca2* deletions during tumorigenesis.

**Figure 5:**
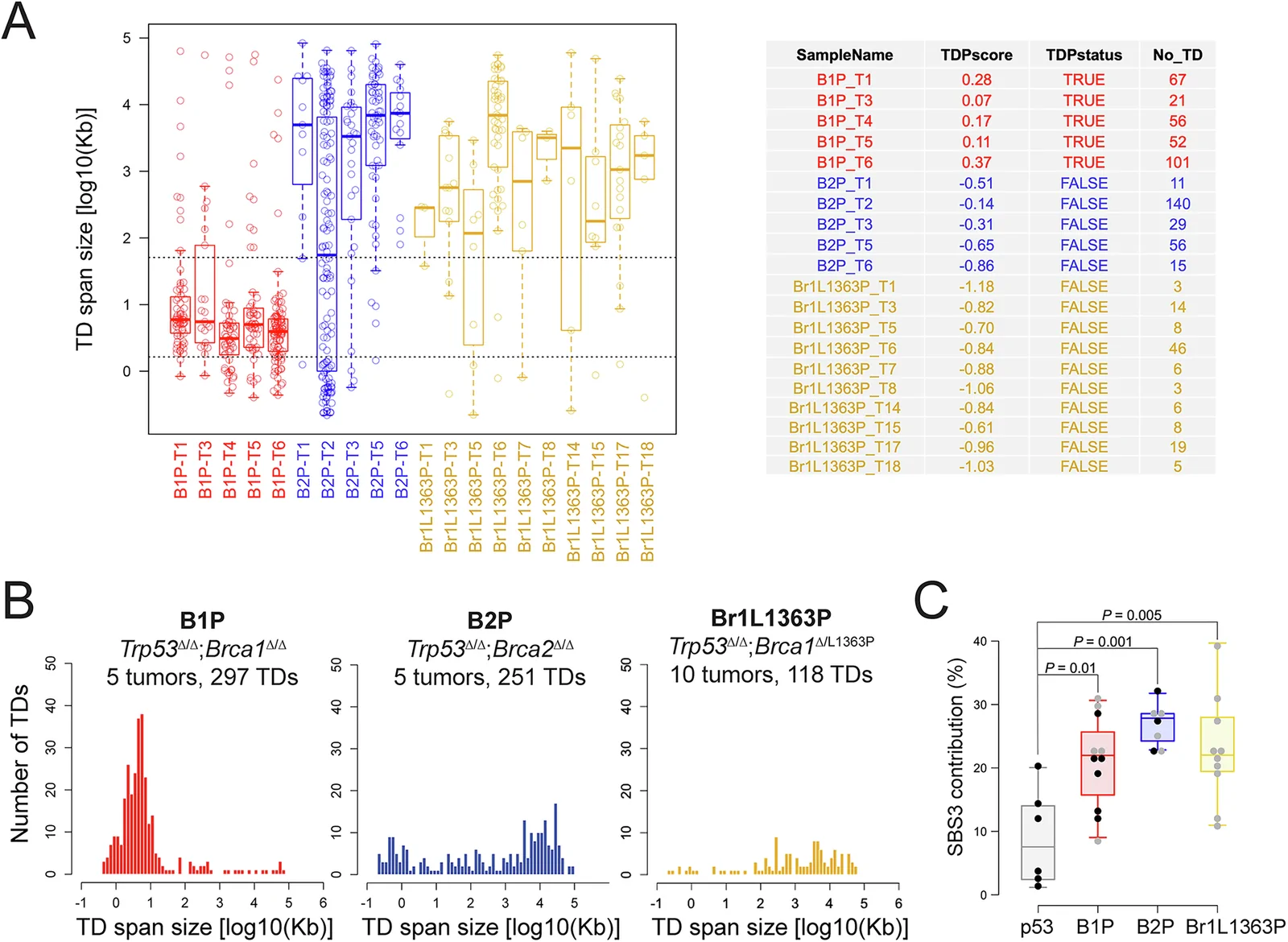
Analysis of tandem duplications in *Brca* p.L1363P mouse mammary tumors. (**A**) Left panel: Distribution of TD span sizes in mouse mammary tumor genomes of different *Brca* genotypes. B1P (*n* = 5): *Brca1*^∆5-13/∆5-13^;*Trp53*^∆2-10/∆2-10^. B2P (*n* = 5): *Brca2*^∆11/∆11^;*Trp53*^∆2-10/∆2-10^. Br1L1363P (*n* = 10): *Brca1*^∆5-13/L1363P^;*Trp53*^∆2-10/∆2-10^. The dotted horizontal lines denote the TD span size range for Group 1 TDs. Note abundant Group 1 TDs in B1P tumor genomes but not in B2P or Br1L1363P tumor genomes. The center line represents the median (50th percentile), the lower and upper bounds of the box represent the first (25th percentile) and third (75th percentile) quartiles, respectively, and the whiskers extend to the minimum and maximum values within 1.5 times the interquartile range from the first and third quartiles; values beyond the whiskers are plotted individually as outliers. Right panel: TDP scores for each tumor genome. 5/5 B1P tumors score as Group 1 TDP but no B2P or Br1L1363P tumors score as TDP. (**B**) TD span size distribution in pooled tumors of each *Brca* genotype. Mann–Whitney *U* test of TD size distributions: B1P vs B2P: *P* = 1.4E-22. B1P vs Br1L1363P: *P* = 5.7E-32. B2P vs Br1L1363P: not significant (ns). (**C**) Quantitation of SBS3 mutation signature in the genotypes shown. These data include both current data (gray data points) and historical data from a previous study (black data points) that includes negative control *Trp53*^∆2-10/∆2-10^ tumors (P53) in which *Brca* alleles are wild-type. Statistical comparisons by Student’s *t* test (two-tailed); *P* values as shown. B1P *vs*. B2P: ns. B1P *vs*. Br1L1363P: ns. B2P *vs*. Br1L1363P: ns. The median (50th percentile) is represented by the center line, while the first (25th percentile) and third (75th percentile) are represented by the lower and upper bounds of the box, respectively. Whiskers represent the extent of minima and maxima values within 1.5 times the interquartile range from the first and third quartiles; individual outliers are plotted beyond the whiskers. Quantitation of the SBS3 mutation signature in the genotypes shown. This data includes both current data (gray data points, *n* = 19) and historical data from a previous study (black data points, *n* = 15) that includes negative control Trp53∆2-10/∆2-10 tumors (P53, *n* = 6) in which *Brca* alleles are wild-type.

We assessed the TD configuration of each tumor genome on a whole-genome scale. For the definition of the TDP score formula, see (Menghi et al, 2016). We noted abundant Group 1 TDs in 5/5 B1P tumor genomes in sufficient numbers to be scored as TDP Group 1, in agreement with Menghi et al (Menghi et al, 2018) (Fig. 5A,B). In contrast, 10/10 Br1L1363P and 5/5 B2P tumor genomes revealed no enrichment of Group 1 TDs, failed to score as TDP, and revealed statistically similar distributions of TD span sizes (Fig. 5A,B). One B2P tumor sample, B2P-T2, showed an exceptional rearrangement pattern, with hyperabundance of TDs, mostly of span size ≤1 kb or >100 kb (Fig. 5A). However, the B2P-T2 tumor failed to score as TDP by the algorithm of Menghi et al (Menghi et al, 2016), because the regions of intense instability were not dispersed genome-wide, as is characteristic of TDP cancer genomes. Furthermore, the B2P-T2 genome revealed single-nucleotide variant frequencies ~100-fold higher than all other samples (Appendix Fig. S4D), suggesting a complex origin of this excessive genomic instability. The 5 B2P tumors and 10 Br1L1363P tumors revealed statistically similar TD spectra mostly devoid of the *Brca1*-deficiency-linked Group 1 TDs. Most TDs in B2P and Br1L1363P tumor genomes were >100 kb, as is typical of the generic cancer genome (Li et al, 2020; Scully et al, 2025). Comparison to historical *Trp53*^∆2-10/∆2-10^ controls showed that tumor genomes from all groups in which a *Brca* gene had been targeted revealed statistically significant elevations in the abundance of SBS3, a well-characterized single-base substitution mutational signature widely recognized as a hallmark of HR deficiency (Fig. 5C) (Nik**-**Zainal et al, 2016). The findings that *Brca1* p.L1363P drives accelerated tumorigenesis in the mouse (Pulver et al, 2021) and that Br1L1363P tumor genomes show the distinctive SBS3 mutation signature strongly suggest that *Brca1* p.L1363P is a pathogenic variant.

Taken together, despite being defective for HR, *Brca1* p.L1363P remains competent for suppression of genome-wide Group 1 TD formation during tumorigenesis. The Tus/*Ter* system accurately predicted this separation-of-function phenotype of *Brca1* p.L1363P-driven tumors.

### *Brca1* CC domain mutants tolerate *Fancm* deletion

DNA end resection-defective *Brca1*^∆11/hyg^ mES cells reveal abundant TDs when depleted of FANCM and are synthetic lethal/synthetic sick on a *Fancm* null background (Panday et al, 2021). To better understand the relationships between Group 1 TD formation, DNA end resection, and *Fancm* synthetic lethality, we asked whether *Brca1* CC domain mutants tolerate *Fancm* deletion. To this end, we used CRISPR/Cas9-mediated dual DSB incisions to delete the DEAH (ATP binding) motif of the FANCM ATPase domain and to introduce an 85 bp frame-shift within *Fancm* exon 2, generating a functionally null allele of *Fancm* (Panday et al, 2021). See “Methods” for details. We generated clones of *Fancm*^+/–^ and *Fancm*^–/–^ mES cells on different *Brca* genetic backgrounds: *Brca1*^fl11-HA/–^, *Brca1*^L1363P-HA/–^, *Brca1*^CC∆9bp-HA/–^, *Brca1*^∆CC-HA/–^, *Brca1*^∆11-HA/–^, and *Brca2*^∆27/∆27^ (Morimatsu et al, 1998) (Appendix Fig. S5A). Each *Fancm*^–/–^;*Brca1*^∆11-HA/–^ clone was extremely slow-growing, indicating a synthetic sick phenotype. We quantified Tus/*Ter*-induced TDs in the *Fancm*^–/–^;*Brca* mutants derived above. *Fancm*^–/–^;*Brca1*^∆11-HA/–^ clones revealed robust TD induction. In contrast, *Fancm*^–/–^;*Brca1*^L1363P-HA/–^ and *Fancm*^–/–^;*Brca2*^∆27/∆27^ clones revealed frequencies of Tus/*Ter*-induced TDs similar to or lower than those seen in *Fancm*^–/–^;*Brca1*^WT-HA/–^ cells (Appendix Fig. S5B).

**Figure 6:**
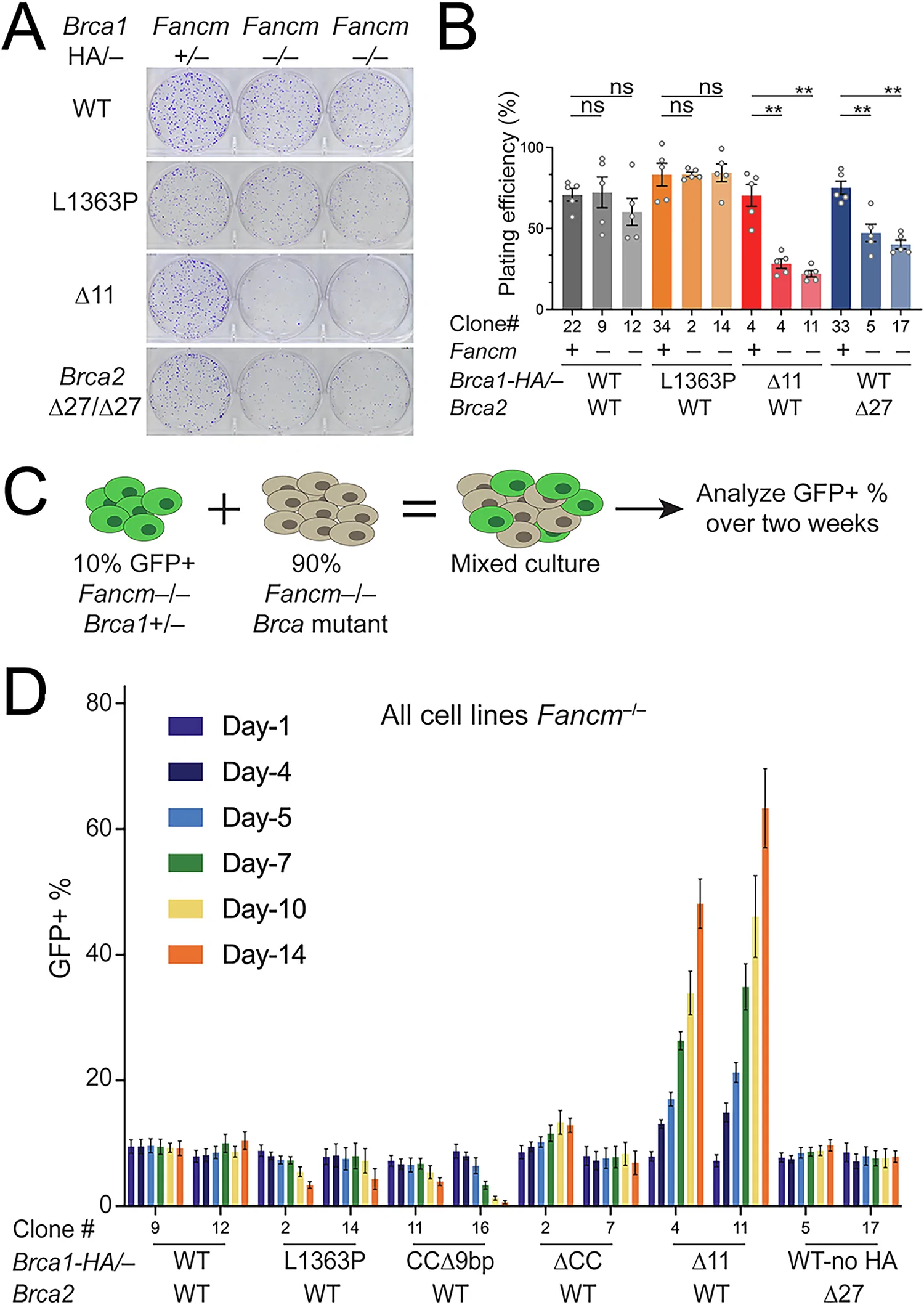
*Fancm* deletion is tolerated by *Brca1* p.L1363P cells. (**A**) Representative images of colony formation in the genotypes shown. *Brca1* allele abbreviations as in Fig. 1A. Note severe loss of colony-forming ability of two independent *Fancm*^–/–^;*Brca1*^∆11-HA/–^ clones. (**B**) Quantitation of colony formation assays in five independent biological replicates (*n* = 5; data shows mean and s.e.m. of five independent biological repeats). Unpaired *t* test with Welch’s correction. ns: not significant, **P < 0.005. (**C**) Schematic outline of competitive growth assay. (**D**) Competitive growth assays of the *Fancm*^–/–^;*Brca* genotypes shown. Bars show mean and s.e.m. of GFP^+^ % at the timepoints shown in four independent experiments (*n *= 4). Note progressive enrichment of GFP^+^-marked *Fancm*^–/–^;*Brca1*^fl11-HA/–^ cells when mixed with *Fancm*^–/–^;*Brca1*^∆11-HA/–^ cells. Source data are available online for this figure.

We quantified the growth properties of selected *Fancm*^–/–^;*Brca* mutants using two independent assays: plating efficiency/colony formation assays and competitive growth assays. We noted a dramatic loss of plating efficiency in two independent clones of *Fancm*^–/–^;*Brca1*^∆11-HA/–^ cells, in comparison to *Fancm*^+/–^;*Brca1*^∆11-HA/–^ isogenic controls (Fig. 6A,B). In contrast, two independent clones of *Fancm*^–/–^;*Brca1*^L1363P-HA/–^ cells revealed no loss of plating efficiency in comparison to isogenic *Fancm*^+/–^;*Brca1*^L1363P-HA/–^ cells. There was a modest reduction in plating efficiency in two independent clones of *Fancm*^–/–^;*Brca2*^∆27/∆27^ cells. To perform competitive growth assays, we mixed ~10% GFP^+^ *Fancm*^–/–^;*Brca1*^fl11-HA/–^ cells (WT controls) with ~90% GFP^–^ *Fancm*^–/–^cells of the test *Brca* genotypes. We then cultivated the mixed populations over a 2-week period, harvesting aliquots at defined timepoints for FACS quantitation of the GFP^+^ fraction (Fig. 6C,D). As expected, the *Brca1* WT (GFP^–^) *Fancm*^–/–^;*Brca1*^fl11-HA/–^ sample produced GFP fractions that were stable over the 2-week period. In contrast, *Fancm*^–/–^;*Brca1*^∆11-HA/–^ cultures revealed progressive increases in the GFP fraction over time, indicative of a growth defect in the GFP^–^ *Fancm*^–/–^;*Brca1*^∆11-HA/–^ cells in comparison to the GFP^+^ *Fancm*^–/–^;*Brca1*^fl11-HA/–^ co-cultured cells. We noted no competitive growth deficit in the *Fancm*^–/–^;*Brca2*^∆27/∆27^ cells. Two independent *Fancm*^–/–^;*Brca1*^L1363P-HA/–^ clones and two independent *Fancm*^–/–^;*Brca1*^CC∆9bp-HA/–^ clones revealed a modest *reduction* in the GFP fraction during the 2-week co-culture period, suggestive of a modest growth advantage over the GFP^+^ *Fancm*^–/–^;*Brca1*^fl11-HA/–^ co-cultured cells.

**Figure EV4:**
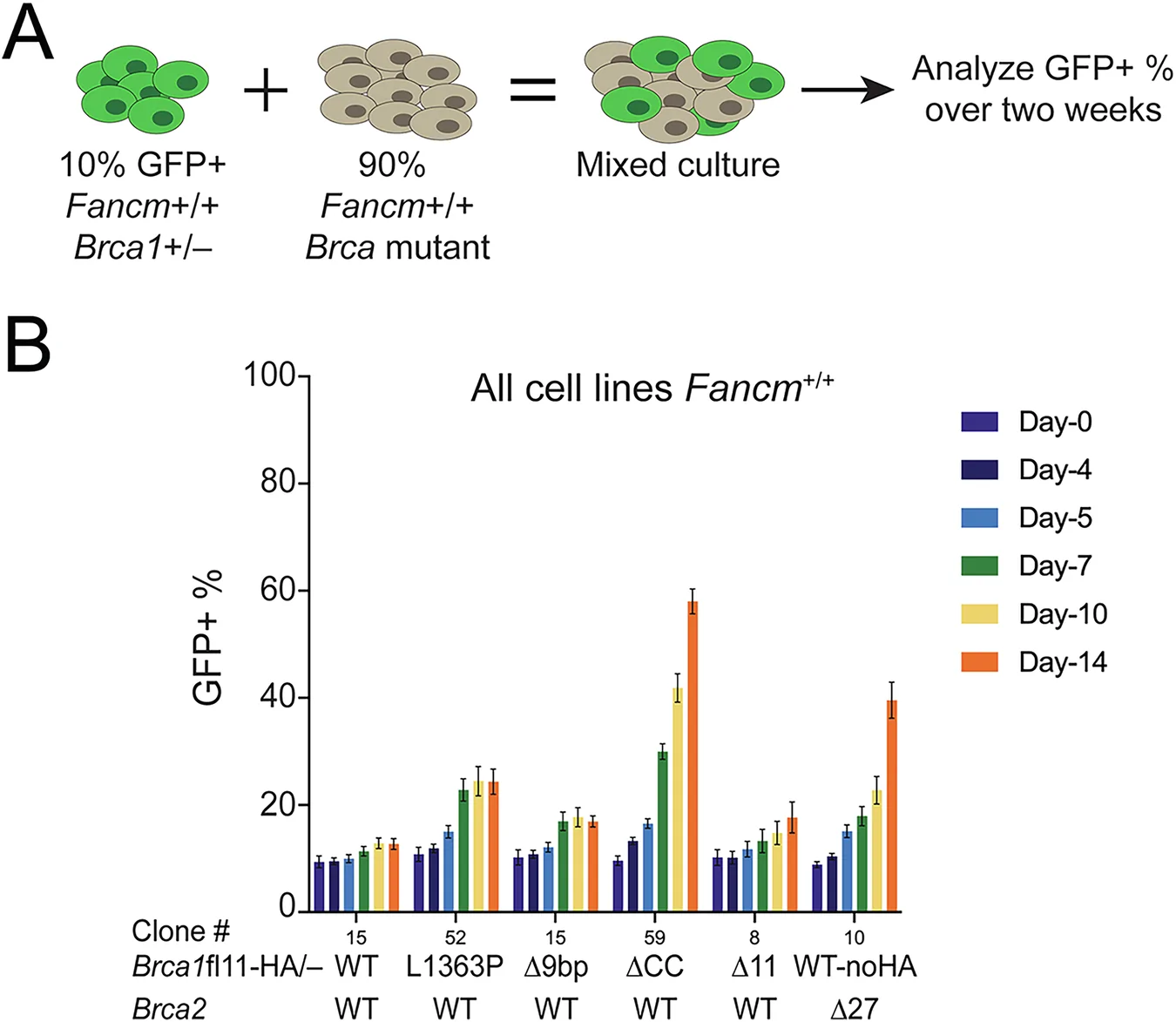
Controls for Fig. 6: competitive growth of *Brca* mutant cell lines on *Fancm* WT background. (**A**) Schematic outline of competitive growth assay; all cells are *Fancm*^+/+^. (**B**) Competitive growth assays of different *Fancm*^+/+^;*Brca* genotypes. Bars show mean and s.e.m. of GFP^+^ % at the timepoints shown across 6 independent experiments (*n* = 6). Note degree of enrichment of GFP^+^-marked *Fancm*^+/+^;*Brca1*^fl11-HA/–^ cells when mixed with different *Fancm*^+/+^;*Brca* mutant cells. Comparison with Fig. 6 shows that deletion of *Fancm* alters competitive growth properties, disadvantaging *Brca1*^∆11-HA/–^ cells but improving the relative growth of *Brca1* CC mutants and *Brca2*^∆27/∆27^ cells.

To determine the extent to which *Fancm* deletion had caused the observed changes in growth, we repeated the competitive growth assays on parental (i.e., *Fancm* WT) clones of the above-noted *Brca* genotypes, using GFP^+^ *Fancm*^*+/+*^;*Brca1*^fl11-HA/–^ cells as the co-cultured competitors (Fig. EV4). In this setting, *Brca1*^∆CC-HA/–^ and *Brca2*^∆27/∆27^ cells revealed the most severe growth deficit, whereas *Brca1*^∆11-HA/–^, *Brca1*^L1363P-HA/–^, and *Brca1*^CC∆9bp-HA/–^ cells revealed only a modest growth deficit in comparison to the GFP^+^ *Brca1*^fl11-HA/–^ co-cultured cells. These results show that *Fancm* deletion had conferred a severe growth defect on *Brca1* ∆11 mutants, as reflected in both colony formation and competitive growth assays. In contrast, *Brca1* CC domain mutants and *Brca2*^∆27/∆27^ cells acquire modestly improved competitive growth characteristics in the absence of *Fancm*. These divergent effects of *Fancm* deletion match those of a recent CRISPR screen in mES cells (preprint: Dossin et al, 2026). Our data, although limited to a small number of *Brca1* alleles, suggest that synthetic lethality/sickness following *Fancm* loss in *Brca1* mutants is correlated with defective DNA end resection function but not with overall HR capacity (Table 1). Further testing of this hypothesis will require analysis of additional *Brca1* mutant alleles.

**Table 1:** Summary of relationships between *Brca* genotype, DNA end resection function, DSB-induced HR, Group 1 TD suppression, and *Fancm* synthetic lethality.

*Brca1*	*Brca2*	DNA end resection	DSB-induced HR	Group 1 TD suppression	Impact of *Fancm* deletion
WT	WT	Competent	WT	Competent	Viable
∆11	WT	Defective	Impaired	Defective	Synthetic sick/lethal
CC mutant	WT	Competent	Impaired	Competent	Viable
WT	∆27	Competent	Impaired	Competent	Viable

## Discussion

Here, we compare two classes of *Brca1* mutant alleles in stalled-fork HR and in Group 1 TD formation. BRCA1 CC mutant proteins are defective for interaction with PALB2, leading to a defect in RAD51-mediated HR (Anantha et al, 2017; Dray et al, 2010; Sy et al, 2009; Xia et al, 2006; Zhang et al, 2009). However, unlike the majority of cancer-associated *BRCA1* mutants, BRCA1 CC mutants remain competent for DNA end resection because they retain the key BRCA1 domains (RING, tandem BRCT, and exon 11-encoded region) required for this function (Ceppi et al, 2024; Salunkhe et al, 2024). *Brca1* ∆11 and *Brca1* CC mutants are complementary loss-of-function alleles, as shown by interallelic complementation in mouse development (Nacson et al, 2020). A likely explanation of this phenomenon is that *Brca1* CC mutants provide DNA end resection capability, while *Brca1* ∆11 mutants facilitate PALB2 recruitment for HR. In the mES cell system studied here, we also observed reduced PALB2 binding by endogenous BRCA1 ∆11 in comparison to WT BRCA1. This defect was more severe than that noted previously in human cancer cells expressing exogenous *BRCA1* alleles (Nacson et al, 2020). Thus, although *Brca1* ∆11 mutants are DNA end resection-defective, we cannot exclude the possibility that coordination between BRCA1 ∆11 and PALB2-BRCA2-RAD51 is also somewhat impaired. The minimal PALB2-binding region of mouse BRCA1 comprises residues 1350-1377 (PDB: 7K3S). Conceivably, the full interface between BRCA1 and PALB2 might extend to *Brca1* exon 11-encoded regions neighboring the CC domain.

The effects of *Brca1* exon 11 deletion and CC mutation on stalled fork HR, DSB-induced HR, and PARPi sensitivity were qualitatively similar but revealed interesting quantitative differences. *Brca1* ∆CC mutant cells, in which the entire CC domain is deleted, revealed greater sensitivity to the PARP inhibitor Olaparib and more severe STGC defects than *Brca1* L1363P or ∆9 bp cells, in which the BRCA1 coiled coil domain is disrupted but not fully deleted. This difference may reflect a more complete loss of PALB2 binding by BRCA1 ∆CC than by BRCA1 L1363P or BRCA1 ∆9 bp. Interestingly, stalled fork (Tus/*Ter*-induced) STGC failed to accurately predict PARPi sensitivity. This is most strikingly seen in *Brca1* ∆11 cells, which were among the most PARPi-sensitive but revealed the mildest defects in Tus/*Ter*-induced STGC of all *Brca1* mutants studied here. Similarly, we previously noted that *Brca1* BRCT domain mutant mES cells reveal no defect in Tus/*Ter*-induced STGC, despite being PARPi sensitive and defective for DSB-induced STGC (Chandramouly et al, 2013; Farmer et al, 2005; Willis et al, 2014). This discrepancy suggests that the DNA end resection function of BRCA1 is less critical for Tus/*Ter*-induced STGC than it is for I-SceI-induced STGC or for PARPi resistance. Similar effects were noted in recent studies of nickase-induced repair, where *Brca1* was found to be dispensable for DNA end resection at nickase-broken forks, and *Brca1* ∆11 mutants revealed wild-type levels of nickase-induced STGC (Elango et al, 2025; Pavani et al, 2024; Scully et al, 2024; Zong et al, 2026). Taken together, current evidence suggests that DNA end resection at stalled or broken forks is less tightly regulated than at a replication-independent DSB. The avid engagement of DNA end resection at stalled or broken forks may also explain why classical nonhomologous end joining (cNHEJ) does not compete with HR in nickase-induced broken fork repair or in stalled fork repair (Elango et al, 2025; Scully et al, 2019; Vriend et al, 2016; Willis et al, 2018).

**Figure EV5:**
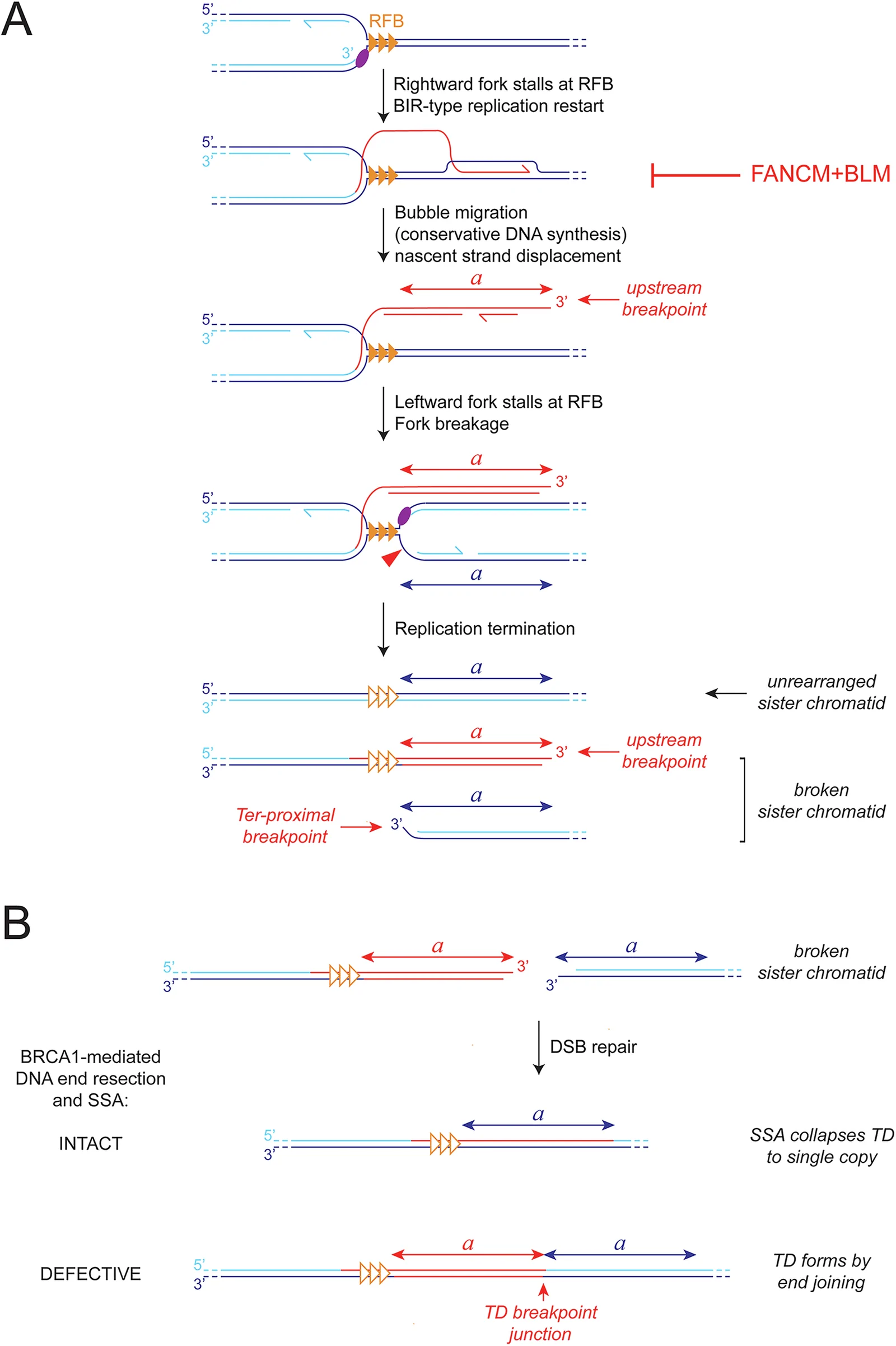
Proposed mechanisms of action of FANCM and BRCA1 in suppression of Tus/*Ter*-induced Group 1 TDs. (**A**) Interaction of converging replication forks with Tus/*Ter* replication fork barrier (RFB) sets up the Group 1 TD intermediate. The Tus/*Ter*-induced TD breakpoint junction is formed from one breakpoint in close proximity to the *Ter* array (the ‘*Ter*-proximal’ breakpoint) and one ‘upstream’ breakpoint several kilobases from the *Ter* array (Willis et al, 2017). We infer that for one fork (the rightward fork shown here), replication extends beyond the Tus/*Ter* RFB by an aberrant replication restart mechanism, while the second fork (here, the leftward fork) stalls and is processed at the Tus/*Ter* RFB site. We propose that the rightward fork stalls at the RFB and is restarted as a migrating bubble (i.e., BIR-type conservative DNA synthesis; red). This process could be *RAD51*-dependent or -independent (Panday et al, 2021; Scully et al, 2021). FANCM, in partnership with BLM, suppresses this form of replication restart by disassembling migrating bubble BIR intermediates. Eventual termination of BIR generates a displaced nascent strand that spans the potentially duplicated segment (*a*). The 3’ end of the displaced nascent strand forms the potential upstream breakpoint of the TD. The leftward fork stalls at the RFB and either undergoes fork breakage as shown or fork reversal (not shown). The resulting solitary DNA end forms the potential *Ter*-proximal breakpoint. Completion of DNA replication through the RFB generates one intact sister chromatid and one broken sister chromatid that contains two copies of the TD segment *a*, flanking the break site. (**B**) BRCA1 acts on the broken sister chromatid to suppress Group 1 TD formation. If BRCA1-mediated DNA end resection and SSA functions are intact, the two copies of the duplicated segment *a* flanking the break site are collapsed to a single copy by BRCA1-mediated SSA. If BRCA1-mediated SSA is disabled, end joining opportunistically repairs the break, generating a Group 1 TD. The breakpoint junction is formed by end joining between the upstream breakpoint and the *Ter*-proximal breakpoint.

Tandem duplications are common structural variants in the cancer genome, but the mechanisms that form them remain poorly understood (Li et al, 2020; Scully et al, 2025). The ability to recapitulate *BRCA1*-linked Group 1 TD formation in the Tus/*Ter* system provides an opportunity to decipher mechanisms underlying this class of TDs. Our previous work led us to propose that Group 1 TDs form at a Tus/*Ter* RFB in cells lacking BRCA1 by a replication restart/bypass mechanism, resulting in re-replication of the TD segment, with completion of the TD by means of an end joining step (Scully et al, 2025; Scully et al, 2019; Willis et al, 2017) (Fig. EV5). Our initial findings implicating the DNA end resection mediator CtIP as a Group 1 TD suppressor suggested that DNA end resection might play a role in Group 1 TD suppression (Willis et al, 2017). In the current study, we approached this hypothesis by studying Tus/*Ter*-induced TD formation in different *Brca1* allelic backgrounds, in order to relate BRCA1-mediated Group 1 TD suppression to the known functions of BRCA1 in DSB repair. We find that, in contrast to *Brca1* ∆11 mutants, *Brca1* CC mutants are competent for suppression of Tus/*Ter*-induced TDs. This result supports the hypothesis that BRCA1-mediated DNA end resection, but not BRCA1/PALB2-mediated RAD51 loading, is required for Group 1 TD suppression. We tested the predictive power of the Tus/*Ter* TD assay in a mouse model of *Brca*-linked mammary tumorigenesis (Pulver et al, 2021). Consistent with the predictions of the Tus/*Ter* system, we find that *Brca1* p.L1363P mutant-driven mammary tumors, although defective for HR, do not display a Group 1 TD phenotype. In the same experiment, confirming our previous work, *Brca1* null mammary tumors displayed a robust Group 1 TD phenotype (Menghi et al, 2018). In this regard, *Brca1* p.L1363P mutant cancers more closely resemble *Brca2* mutant cancers, which do not register as TDP (Menghi et al, 2018) (and this study). Thus, the Tus/*Ter* system accurately predicted the absence of a Group 1 TD phenotype in *Brca1* p.L1363P-driven cancers.

The synergistic effects of *Brca1* exon 11 deletion and FANCM depletion on Tus/*Ter*-induced TD formation led us to study the genetic relationship between these two genes (Panday et al, 2021; Willis et al, 2017). We found that *Brca1* ∆11 mES cells are synthetic lethal/sick on a *Fancm* null or FANCM ATP hydrolysis-defective genetic background. If this relationship were to hold for *BRCA1*-linked human cancers, small-molecule inhibitors of FANCM ATPase function might be effective as therapeutics in *BRCA1*-linked cancer. These findings raised the possibility that Group 1 TD formation and *Fancm* synthetic lethality might be mechanistically linked. Here, we tested this hypothesis by comparing the impact of *Fancm* deletion on the growth properties of *Brca1* ∆11 mutants, *Brca1* L1363P CC mutants, and *Brca2* ∆27 mutant cells. By use of two different assays—colony plating efficiency and competitive growth assays—we confirm the synthetic sick phenotype of *Brca1* ∆11 *Fancm* null cells. In contrast, *Fancm* deletion in *Brca1* L1363P CC mutants was well-tolerated, with no defect in colony growth in comparison to *Brca1* WT *Fancm* null cells. Indeed, in competitive growth assays, *Brca1* L1363P *Fancm* null cells displayed a modest growth advantage over *Fancm* null cells that harbor WT *Brca1*. We found that *Fancm* deletion modestly suppressed colony formation but normalized the relative growth of *Brca2* ∆27 cells in comparison to *Fancm* null cells with no *Brca* mutation. Similar differential impacts of *Fancm* loss on different *Brca1* allelic backgrounds and on *Brca2* defective cells have been reported recently (preprint: Dossin et al, 2026). *Brca2* ∆27 mutants disrupt a C-terminal RAD51 binding domain and are defective for classical HR, fork protection and gap suppression, but are competent for suppression of Tus/*Ter*-induced TDs (Lim et al, 2024; Longo et al, 2025; Willis et al, 2017) (Appendix Fig. S5B). It will be interesting to study the impact of *Fancm* deletion in other *Brca2* allelic backgrounds.

**Figure 7:**
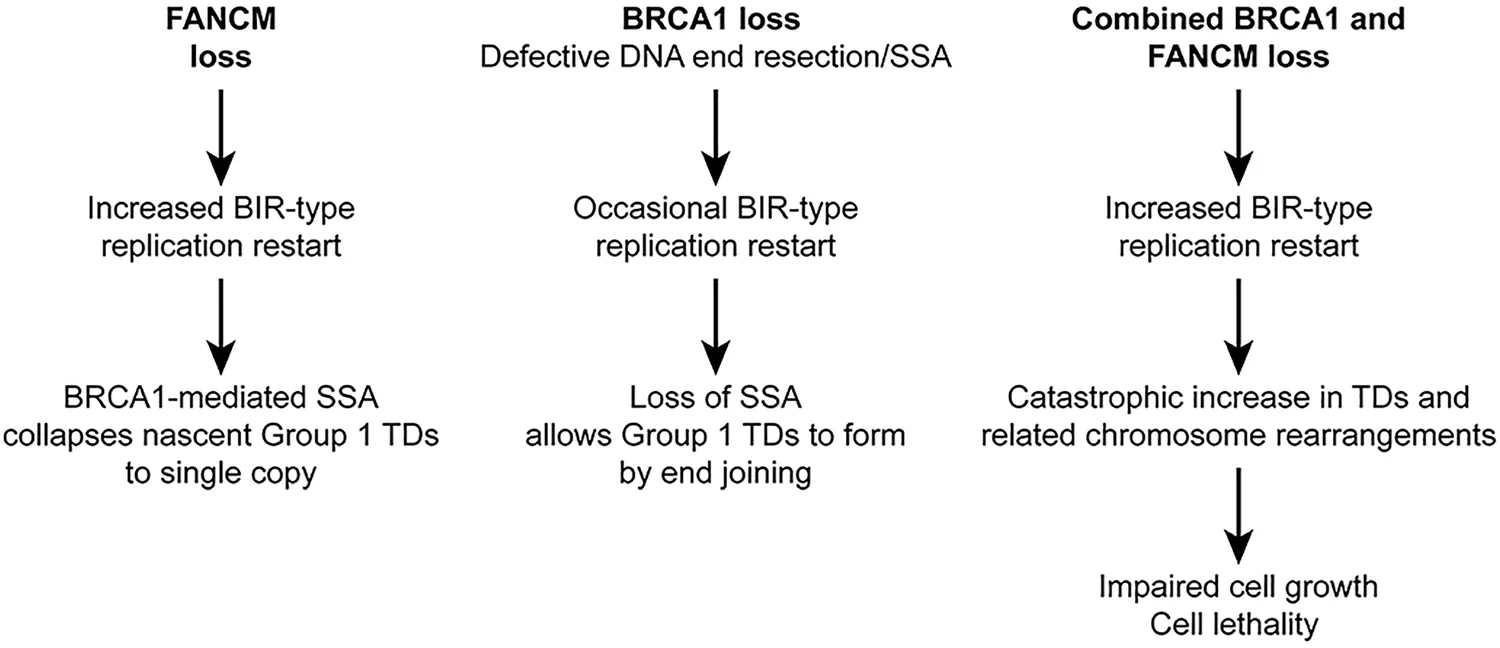
Hypothesis linking defective BRCA1-mediated DNA end resection and SSA to *Fancm* synthetic lethality. *Left*: FANCM loss alone permits BIR-type replication restart at sites of stalled replication. BRCA1-mediated SSA collapses most nascent Group TDs back to a single copy. *Center*: Loss of BRCA1-mediated DNSA end resection abrogates SSA and permits occasional BIR-restarted replication forks to resolve as Group 1 TDs. *Right*: combined loss of BRCA1 and FANCM increases BIR-type restart at stalled forks and combines with SSA defect to massively increase the probability of Group 1 TD formation at sites of replication stalling. The increases in Group 1 TDs and related chromosome rearrangements impair cell fitness, resulting in synthetic sickness/lethality in the double mutant background.

If Group 1 TD formation and *Fancm* synthetic lethality are different consequences of defective BRCA1-mediated DNA end resection, how might these processes be linked? The data suggest that FANCM and BRCA1 act as independent barriers to Group 1 TD formation. We proposed that FANCM (in association with BLM (Panday et al, 2021; Willis et al, 2017)) suppresses break-induced replication (BIR)-type replication restart at stalled forks; BRCA1, *via* its SSA function, provides a ‘failsafe’ mechanism for collapsing back to single copy any nascent Group 1 TDs that escape the action of FANCM/BLM (Fig. EV5A). SSA is dependent on BRCA1-mediated DNA end resection but not on RAD51 loading—it is increased in *Brca2* mutant and *Brca1* CC mutant cells (Anantha et al, 2017; Tutt et al, 2001). Although traditionally considered an error-prone DSB repair pathway, in the context of a nascent Group 1 TD, SSA would perform a conservative (genome-stabilizing) role by preventing TD formation (Fig. EV5B). When both BRCA1 and FANCM are inactivated, two important, independent checks on Group 1 TD formation are lost, unleashing intolerable levels of genomic instability at sites of fork stalling (Fig. 7). One manifestation of this heightened genomic instability is an enhanced Group 1 TD phenotype; the other is the loss of cell fitness, i.e., synthetic lethality.

### Limitations of the study

This study is limited to mouse ES cells and mouse mammary tumors. Two other studies—one in somatic human cells and one in mES cells—have also identified a synthetic lethal relationship between *BRCA1* and *FANCM* mutations (Adam et al, 2021; preprint: Dossin et al, 2026). However, it is not yet clear to what extent the synthetic lethal relationship between *BRCA1* and *FANCM* translates to the cancer setting. The development of therapeutics that specifically target FANCM would facilitate such studies and might ultimately prove effective in the treatment of *BRCA1*-linked cancers. We have limited this study to mutations affecting *Brca1* exon 11 and the CC domain. It will be instructive to study additional domain mutants of BRCA1 and its associated proteins to clarify whether tolerance of *FANCM* loss is genetically separable from BRCA1-mediated DNA end resection (Tsukada et al, 2024). As regards the mechanism of Group 1 TD formation, the link between BRCA1-mediated DNA end resection and TD suppression suggested by our work is correlative; it is possible that some other as yet unidentified function of BRCA1, which maps to the same domains as those required for DNA end resection, is the actual mediator of Group 1 TD suppression. The hypothesis that SSA specifically suppresses Group 1 TD formation is similarly unproven. The mechanisms discussed here apply only to Group 1 TDs, which have much smaller span sizes than most cancer-related TDs. Larger Group 2 or Group 3 TDs, which dominate the TD spectrum of non-*BRCA1*-linked cancers, likely arise by fundamentally different mechanisms, as reflected in their distinct genetic dependencies (Menghi et al, 2018; Menghi et al, 2016; Scully et al, 2025).

The naturally occurring triggers of Group 1 TD formation in the cancer genome remain unknown. Although the Tus/*Ter* fork stalling system has thus far made accurate predictions regarding TD formation in *BRCA1*-linked cancer, additional types of lesions likely contribute to Group 1 TD formation in cancer. Huang et al recently showed that Group 1 TDs can form at a site-specific nick, implying that replication fork breakage can also trigger Group 1 TD formation (Huang et al, 2025). This finding raises the question: which form of replication stress—fork stalling or fork breakage—accounts for the major fraction of Group 1 TDs in a developing *BRCA1*-linked cancer? It will be instructive to compare the genetic regulation of Tus/*Ter*-induced *vs*. nickase-induced Group 1 TD formation and to relate these findings to the genetics of *BRCA1*-linked cancer-associated Group 1 TDs.

## Methods

**Table:** Reagents and tools table

Reagent/resource	Reference or source	Identifier or catalog number
**Experimental models**		
6x *Ter-*HR reporter containing TC1 mouse Embryonic Stem cells (mES)	Willis et al, 2014	
6x *Ter-*HR reporter TC1 (mES) *Brca1* mutant clones	This study	
FVB mice	Pulver et al, 2021	*Brca1*^*L1363P/F*^*;Trp53*^*F/F*^ mice
**Recombinant DNA**		
pcDNA3β-myc NLS-Tus	Willis et al, 2014	
pcDNA3β -myc NLS-I-SceI	Willis et al, 2014	
**Antibodies**		
Anti-HA (for immunoblot)	Abcam	ab9110
Anti-HA (for immunofluorescence)	Abcam	ab18181
Anti-vinculin	Cell Signalling Technologies	#4650
Anti-mouse BRCA1 exon 11	Scully lab	
Anti-RPA (for ChIP)	Abcam	ab10359
Anti-mouse PALB2	Xia lab	
Anti-mouse Alexa Fluor 647	Jackson ImmunoResearch	#115-605-003
Anti-rabbit Alexa Fluor 488	Jackson ImmunoResearch	#111-545-003
**Oligonucleotides and other sequence-based reagents**		
sgRNA oligomer sequences with PAM		
Brca1 Exon 2 sgRNA	cttctgttgacttaccagatcgg	
Brca1 Exon 23 sgRNA	gtctatccagctaccggtgtcgg	
Brca1 CC sgRNA 1	tctgcgagcagtcttcagaaagg	
Brca1 CC sgRNA 4	gatctggaaccaaacatgtcagg	
Brca1 CC sgRNA PE3	gcagcaggaaatggctcacctgg	
Fancm del85 sgRNA5'	ggttcttttcctgacaccgcagg	
Fancm del85 sgRNA3'	gatgaagctcataaggcacttgg	
**Primers**		
*Brca1* hemizygous clone generation		
Deletion junction PCR	Brca1 Exon 2 FWD Primer 2	tgggggtgacttaccttcag
	Brca1 Exon 23 REV Primer 1	acaaatcaggaccactagct
5’ region PCR	Brca1 Exon 2 FWD Primer 2	tgggggtgacttaccttcag
	Brca1 Exon 2 REV Primer 4	atcccacactggacttgcta
3’ region PCR	Brca1 Exon 23 FWD Primer 6	gggcttttaccacgctcact
	Brca1 Exon 23 REV Primer 1	acaaatcaggaccactagct
Deletion of Floxed *Brca1* exon 11	FLOX BRCA1 (P13.63 FWD)	CTGGGTAGTTTGTAAGCATCC
	OUT-FLOX Brca1 (P14.02 REV)	CTGCGAGCAGTCTTCAGAAAG
HA-tagging *Brca1*		
5’ region PCR	B1 tag F3	ggatctctgccagcttaagtacc
	B1 tag R1	CGTAAGGGTAcccACTAGCG
3’ region PCR	B1 tag F5	gtgtctctcactcggaaggac
	B1 tag R5	ggaattagaaggctctccttgacac
*Brca1* ∆CC		
Deletion junction PCR	B1-CC F6	ccagtgtcccaaatgtagcc
	B1-CC R6	tgcactgagcaaagatcacac
5’ region PCR	B1-CC F6	ccagtgtcccaaatgtagcc
	B1-CC R4	gtaggatttttagatctggctggg
3’ region PCR		
	B1-CC F7	cagctcccatgctaaaattaattgc
	B1-CC R6	tgcactgagcaaagatcacac
*Brca1* L1363P		
	B1-pt primer Fw1	tatctggaagagctcgaggg
	L63P_Rev2	tgagccatttcctgctgcag
*Fancm* exon 2		
	Fancm_Fwd_1	ctacctcaagctccagagtcctgg
	Fancm_Rev_1	agttcccatcactgagacttattcc
**Chemicals, enzymes, and other reagents**		
Spy NLS Cas9	New England Biolabs	M0646T
anti-HA beads	Pierce/Invitrogen	88836
P3 solution with supplement	Lonza	V4XP-3032
Alt-R™ CRISPR-Cas9 crRNA	Biosynthesis, IDT	
Alt-R™ CRISPR-Cas9 tracrRNA	IDT	1072533
Alt-R™ S.p. Cas9 Nuclease V3	IDT	1081059
Lipofectamine 2000	Invitrogen	11668019
NEB Ultra II DNA Library Prep Kit	NEB	E7645
KAPA Hyper Prep Kit	KAPA Biosystems/Roche	KK8502
**Software**		
ImageJ	https://imagej.net/software/fiji/#publication	
Keyence BZ-X800 Analyzer	Keyence Corporation, Osaka, Japan	
FlowJo	Becton Dickinson & Company (BD)	FlowJo 10.10.0
bcl2fastq	https://support.illumina.com/sequencing/sequencing_software/bcl2fastq-conversion-software.html	RRID:SCR_015058
samtools	https://github.com/samtools/samtools	RRID:SCR_002105
deeptools	https://github.com/deeptools/deepTools	
PyGenomeTracks	https://github.com/deeptools/pyGenomeTracks	
Burrows-Wheeler Aligner	Li and Durbin, 2009	
**Other**		
CytoFlex	Beckman Coulter	
Celigo imaging cytometer	Revvity	
NextSeq 1000	Illumina	
R230 ultrasonicator	Covaris	
Novaseq X Plus Seq	Illumina	

### Molecular biology, siRNA, and sgRNA oligos

6x*Ter* HR reporters were engineered as previously described Using conventional cloning methods(Willis et al, 2014; Willis et al, 2017). Plasmids used for transfection were prepared using endotoxin-free maxiprep (QIAGEN, 12362) kit and have been described previously (Willis et al, 2014). *Brca1* exon 12 (L1363P) targeting construct was generated using large double-stranded DNA gBlocks (Integrated DNA Technologies). Oligomers and primers used for genotyping, RT-qPCR, and sgRNA synthesis were purchased from Thermo-Fisher Scientific and GeneWiz (Azenta). siRNA SMARTpools were purchased from Horizon Scientific/Dharmacon. Cas9-sgRNA target sites in the mouse genome sequence were identified using the Heidelberg CCtop tool: https://cctop.cos.uni-heidelberg.de. sgRNA in vitro synthesis was performed using the Engen sgRNA Synthesis Kit (New England Biolabs, E3322S) and purified using the Clean and Concentrate Kit (Zymo Research, R1017). sgRNA was electrophoresed on a denaturing gel (Novex 10% TBE-Urea; ThermoFisher Scientific, EC6875BOX), to assess quality prior to transfection.

### Cell culture and cell lines

mES cells are thawed onto plates coated with mouse embryonic fibroblast (MEF) feeders, maintained in ES medium (DMEM with 15% serum, 20 mM HEPES pH 7.6, 0.1 mM bME, 500 U/mL rLIF, 1× MEM NEAA, 2 mM L-glutamine, 100 U/mL penicillin, 100 μg/mL streptomycin, 0.001 M sodium pyruvate) on gelatinized plates, and regularly tested for mycoplasma infection by Myco Alert assay (Lonza, LT07-318). *Brca1*^fl5-13/+^ cells were targeted with a 6x*Ter*-HR reporter as a single copy to the *Rosa26* locus using standard methods described previously (Willis et al, 2014). All cell lines for analysis of *Brca1* separation-of-function mutant alleles were derived from a *Brca1*^fl11/hyg^ founder cell line carrying one *Brca1* allele in which most of the conventionally termed exon 11 (NCBI exon 10) is replaced by a hygromycin resistance cassette, and one functionally wild-type conditional *Brca1* allele in which exon 11 is flanked by *LoxP* elements (Xu et al, 1999). The founder cell line contains a single copy of the 6x*Ter*-HR reporter, targeted as a single copy to the *Rosa26* locus (Willis et al, 2014). All cell lines were regularly tested for mycoplasma contamination and were found to be mycoplasma-free in all tests.

### Generation of *Brca1* mutant cell lines

To delete floxed alleles of *Brca1*, cells were treated with adeno-Cre for 24 h and then plated on 60-mm dishes with MEF feeder layer without any selection. Clones were picked after 4–5 days and expanded for another 5 days. Genomic DNA from clones was prepared using Qiagen Puregene buffers and protocol. The clones were then assessed to check for recombined *LoxP* sites using primers specific for the deleted or undeleted conditional allele (see “Reagents table”). To generate *Brca1* hemizygous cell lines, sgRNA against NCBI exon 2 and 23 (see “Reagents table”) were designed and synthesized as described above. Cas9-sgRNA RNP was pre-assembled in vitro in OptiMEM by mixing 3 µM Spy NLS Cas9 (New England Biolabs, M0646T) and 3 µM purified sgRNAs. Cells were transfected using Lipofectamine 2000 mixed with RNP complexes. 48 h after transfection, cells were plated onto 60 mm dishes containing feeder MEFs without selection. Individual clones were picked for expansion between 4 and 5 days and then expanded on MEFs for another 5 days. *Brca1* hemizygous clones were identified by PCR and confirmed by sequencing using primers spanning the deleted *Brca1* allele (see “Reagents table”).

For in-frame *Brca1* HA-tagging, we generated a targeting construct that positions 8xHA tag sequences in-frame with the 3’ end of the *Brca1* open reading frame, followed by a SMASh degron and a neomycin resistance gene downstream of self-cleaving peptide T2A sequences. Cas9 RNP complexes targeting *Brca1* NCBI Exon 23 (see “Reagents table”) were co-transfected with a linearized targeting construct using Lipofectamine 2000. 48 h after transfection, cells were plated onto 60-mm dishes coated with a feeder layer of MEFs that express the neomycin resistance gene. Twenty-four hours after seeding, 4 µM G418-containing medium was added to the plates. Five to seven days later, G418-resistant colonies were isolated and expanded. Colonies were confirmed using PCR and Sanger sequencing using primers to detect successful integration of the HA-SMASh elements (see “Reagents table”). The clones were also assessed for full-length *Brca1* gene product expression using immunoblotting against HA, as described in the “Results” section.

Domain mutations in the BRCA1 coiled coil were also generated using CRISPR/Cas9 gene editing. To generate ∆CC clones, sgRNAs targeting NCBI exon 11 and 12 (see “Reagents table”) were designed, synthesized in vitro, and transfected as RNP complexes using Lipofectamine 2000. Clones were generated as mentioned above, were grown without selection, and were screened by PCR followed by sequencing using primers for ∆CC Brca1 (see “Reagents table”).

*Brca1* p.L1363P point mutant clones were generated by transfecting a template containing specific mutations in a region of *Brca1* NCBI exon 12. For the p.L1363P mutation, *ctg* was mutated to *ccg*, along with silent mutations in the PAM site of the sgRNA used (see “Reagents table”). Primers *Brca1* L63P (see “Reagents table”) were used to identify clones through PCR and sequencing.

### Generation of mouse Fancm hemizygous and null clones

Characterized *Brca1*^HA/–^ (WT, L1363P and ∆11) and *Brca2*^∆27/∆27^ (deleted for exon 27) mutant clones were subjected to CRISPR/Cas9 gene editing to generate *Fancm* + */–* and *Fancm–/–* clones. sgRNAs targeting exon 2 (see “Reagents table”) of the *Fancm* gene were designed to generate an 85 bp frame-shift deletion in the coding region of *Fancm*. We used CRISPR/Cas9-mediated dual DSB incisions to delete the DEAH (ATP binding) motif of the FANCM ATPase domain and to introduce an 85 bp frame-shift within *Fancm* exon 2, generating clones of *Fancm*^+/–^ mES cells on different *Brca* genetic backgrounds: *Brca1*^fl11-HA/–^, *Brca1*^L1363P-HA/–^, *Brca1*^∆11-HA/–^, and *Brca2*^∆27/∆27^. We then repeated the CRISPR/Cas9 transfection to delete the residual WT *Fancm* allele. This two-step method was necessary because the efficiency of biallelic *Fancm* deletion in *Fancm*^+/+^ cells was low. We retrieved viable *Fancm*^+/–^ or *Fancm*^–/–^ derivatives on all *Brca* genetic backgrounds. Clones were grown without selection, isolated, and expanded as described above. *Fancm* mutant clones and isogenic controls that retained WT *Fancm* were identified through PCR (see “Reagents table”) followed by Sanger sequencing the region of interest after TA cloning.

### Recombination assays

In total, 1.6 × 10^5^ mES cells were transfected in suspension with 0.5 μg empty vector or pcDNA3β-myc NLS-Tus, or pcDNA3β -myc NLS-I-SceI using Lipofectamine 2000 (Invitrogen) (Puget et al, 2005; Willis et al, 2014). Transfection efficiency was measured in parallel by transfecting 0.05 μg wild-type GFP expression vector and 0.45 μg control vector. For experiments with siRNAs, 0.3 µg of plasmid vectors were transfected along with 10 pmol siRNA for each of the target gene or along with 10 pmol of luciferase siRNA. 72 h after transfection, 3 × 10^5^–6 × 10^5^ total events were scored for each sample, and GFP + RFP–, GFP + RFP+, and GFP–RFP+ frequencies were analyzed through flow cytometry using a Beckman Coulter CytoFlex, in duplicates. Reported repair frequencies were corrected for background events and for transfection efficiency (50–85%).

### Colony formation assays

To study the effect of PARPi on different *Brca1* mutants, approximately 800 cells were seeded onto six-well tissue culture plates after coating them with 0.1% gelatin. 24 h after seeding the cells, medium was replaced, and the plates were replenished with PARPi-containing medium. Six days after plating the cells, colonies were fixed using Carnoy’s fixative (75% methanol and 25% glacial acetic acid) and stained with crystal violet. The colonies were counted using a Celigo imaging cytometer (Revvity). For plating assays of *Fancm*^–/–^ clones, an equal number (400) of cells were plated onto six-well cell culture dishes and allowed to grow for 6 days, after which the colonies were processed as described earlier.

### Competitive growth assays

In total, 0.04 × 10^6^ GFP^+^ *Brca1*^fl-HA/–^*;Fancm*^*–/–*^ cells were mixed with 0.36 × 10^6^ GFP^–^ *Brca* mutant *Fancm*^*–/–*^ cells. Approximately 0.02 × 10^6^ cells were seeded onto a six-well plate for growth over 2 weeks, and the GFP^+^ fractions were quantified by flow cytometry on the days shown in Figs. 6 and EV4.

### Immunoprecipitation

In total, 500 µL of NETN300 buffer with protease inhibitors was added onto a 60–70% confluent 100 mm cell culture dish and incubated on ice for 30 min. The lysate was then scraped and collected in a microfuge tube and spun down at 14 K RPM in a refrigerated microcentrifuge at 4 °C for 20 min. The supernatant was collected for immunoprecipitation. Approximately 600 µg of protein was added to 25 µL of anti-HA beads (Pierce) and rotated on an end-to-end rotator for 16–20 h at 4 °C. The beads were then washed twice with lysis buffer, followed by one wash with 1xTBST (Tween20) and a final wash with ultrapure nuclease-free water. The eluate was obtained by incubating the beads with 60 µL of 1× non-denaturing SDS loading buffer at 95 °C for 5 min. Samples were electrophoresed on a SDS-TAE 3–8% gradient gel overnight, and the protein was transferred onto a nitrocellulose membrane for analysis by immunoblotting.

### Western blotting

RIPA buffer was added to the cell pellet and incubated on ice for 30 min, following which the lysate was sonicated and spun down at 14 K RPM in a refrigerated microcentrifuge at 4 °C for 20 min. Protein was estimated using the Thermo Rapid BCA Gold kit. Approximately 30 µg of protein was used for electrophoresis in a TAE 3–8% gradient gel for 16–20 h overnight at 50 V in a cooled chamber. Protein was transferred onto a nitrocellulose membrane using wet transfer in a transfer buffer containing 20% methanol. Following transfer, the membrane was blocked in 5% skimmed milk for anti-HA and anti-vinculin immunoblots, whereas 5% BSA was used for anti-PALB2 immunoblot. After incubating the membranes with the appropriate secondary antibody, Pico PLUS Chemiluminescent Substrate was used to visualize the immunoblot signals using a BioRad ChemiDoc MP.

### Immunofluorescence staining and microscopy

In all, 22 × 22 mm coverslips were disinfected using 70% ethanol and UV-C radiation in the biosafety hood. Coverslips were then coated with 20 µg/mL of fibronectin in 1× PBS and incubated in the biosafety hood at RT. After 30 min fibronectin was aspirated and the coverslips were dried for another 30 min. Coverslips were then rinsed with 1× PBS and ~1 × 10^6^ cells were plated for staining the next day. On the day of staining, coverslips were washed twice with 1× PBS, and the cells were fixed with fixative solution (1× PBS, 3% paraformaldehyde, 2% sucrose) for 10 min at RT. After rinsing the coverslips thrice with 1xPBS, the cells were permeabilized for 2 min using ice-cold TX solution (0.5% Triton-X-100, 20 mM HEPES pH 7.4, 50 mM NaCl, 3 mM MgCl_2_, 300 mM sucrose) while keeping the plates with coverslips on ice. It is critical to wash the coverslips five times with 1× PBS after permeabilization. In total, 1:500 dilution of anti-HA and anti-BRCA1”E” antibodies was added to the hybridization buffer (5% goat serum, 1× PBS, 0.5% NaN_3_), and ~100 µL was added onto each coverslip, followed by incubation at 37 °C for 20 min. Coverslips were washed twice with 1× PBS. In total, ~100 µL of 1:400 dilution of secondary antibodies in the hybridization buffer was added to the coverslips dropwise and followed with another incubation at 37 °C for 20 min. Coverslips were washed twice with 1× PBS and mounted onto slides using Prolong Gold containing DAPI (Invitrogen). Anti-HA Foci were counted using the “Find Maxima” function in ImageJ software. Background subtraction for all images was set at 50 pixels, and prominence for maxima was set at 10,000. The number of cells in the field was counted using DAPI staining, and the mean number of foci per nucleus is reported (Fig. S3A).

### Nucleofection of CRISPR-Cas9 RNP

Equal volumes of 100 μM crRNA (Biosynthesis, IDT) and 100 uM tracrRNA (IDT) were mixed and incubated on a 95 °C heat block for 3 min, then cooled on a benchtop for 5 min. Dialysis buffer (20 mM HEPES pH 7.5, 500 mM KCl, 20% glycerol), Cas9 (IDT, 10 μg/μL), and annealed crRNA/tracrRNA were mixed to form RNP and allowed to sit at room temperature for at least 20 min. P3 solution with supplement (Lonza) were freshly mixed and used for nucleofection of trypsinized cells. In total, 2 μL of 100 μM electroporation enhancer (IDT) was added to each nucleofection reaction. Approximately 12 × 10^6^ cells were used for each nucleofection.

### Chromatin-immunoprecipitation (ChIP)

Nucleofected cells were collected via cell scraping in plain DMEM. Cells were fixed with 1% formaldehyde for 10 min and gently agitated by rotation at room temperature, and then quenched with 750 µL of 2 M Glycine for 4 min with rotation at room temperature. Cells were spun at 1200 × *g* for 3 min at 4 °C, washed with PBS, spun, and flash frozen and stored at −80 °C. Protein A magnetic beads (50 μL per sample) were washed with 0.5% BSA three times, then incubated with RPA antibody (abcam 10359, 5 μL per sample). The antibody was allowed to bind to the magnetic beads at room temperature with rotation for 1 to 3 h. After incubation with the antibody, the beads were washed three times with 0.5% BSA in PBS. Cell pellets were removed from −80 °C and resuspended in lysis buffer (50 mM HEPES, 140 mM NaCl, 1 mM EDTA, 10% glycerol, 0.5% Igepal CA-630, 0.25% Triton-X; with pH adjusted to 7.5 using KOH and with 1× protease inhibitors immediately before use (ThermoFisher, 78429) for 10 min at 4 °C with rotation. The lysate was pelleted at 2000 × *g* for 3 min at 4 °C, then resuspended in nuclear lysis buffer (10 mM Tris-HCl pH 8, 200 mM NaCl, 1 M EDTA, and 0.5 mM EGTA; with pH adjusted to 8 using HCl and with 1x protease inhibitors added before use) and allowed to rotate for 5 min at 4 °C. The nuclear lysate was pelleted at 2000 × *g* for 3 min at 4 °C, then resuspended in sonication buffer (10 mM Tris-HCl pH 8, 100 mM NaCl, 1 mM EDTA, 0.5 mM EGTA, 0.1% Na-Deoxycholate, 0.5% N-lauroylsarcosine; with pH adjusted to 8.0 using HCl, and adding 1× protease inhibitor right before use) and sonicated using (Qsonica Q125)) with 30 s of pulse, 30 s off, 55% amplitude for 6 min at 4 °C. Chromatin was spun at 20,000 × *g* at 4 °C. The supernatant was added to a fresh 5-ml LoBind tube, and 1% final volume of TritonX-100 and antibody-bound beads were added. The final volume was increased to 3 mL using the sonication buffer. The mix was allowed to rotate for 8–20 h at 4 °C.

After 8–20 h of rotation, beads were collected and washed 6 times with RIPA solution (50 mM HEPES, 500 mM LiCl, 1 mM EDTA, 1% Igepal CA-630, 0.7% Na-Deoxycholate; pH adjusted to 7.5 using KOH), then once with TBS wash buffer (20 mM Tris-HCl pH 7.5, 150 mM NaCl) and resuspended in elution buffer (50 mM Tris-HCl pH 8, 10 mM EDTA, 1% SDS). Beads and elution buffer were incubated for at least 8 h with shaking at 65 °C for reverse crosslinking. RNaseA was added to the beads and incubated for 15 min at 37 °C, followed by Proteinase K at 55 °C for 1 h. Beads were separated from the eluate using magnetic separation, and the DNA was purified using MinElute column purification.

The ChIP libraries were prepared according to the NEB Ultra II DNA Library Prep Kit (NEB, #E7645). End-repair and A-tailing were performed, followed by adapter ligation (NEB 7600), and 13 cycles of PCR amplification. Samples were then pooled, and DNA was quantified using a Qubit (ThermoFisher) and sequenced on an Illumina NextSeq 1000 machine using paired-end 2×60 bp read length.

### ChIP-Seq data analysis

ChIP-Seq data were demultiplexed using the bcl2fastq software, and the resulting fastq files were aligned to a mouse genome (mm10) containing the HR reporter using bowtie2. Alignments were converted to binary, filtered by quality, and sorted using samtools. Enrichment bigwig files were obtained using deeptools by subtracting the signal from the undamaged sample (No Cas9) from the damaged one (window size of 50 bp). Final plots (shown in Fig. 3B) were generated using the PyGenomeTracks software. ChIP-Seq enrichments (Fig. 3C) were computed using a custom Python code that subtracts the number of normalized (RPM) reads in the damaged and undamaged samples in a window of 30 kb centered at the cut site. Analysis code is available on GitHub (https://github.com/AlbertoMarinG/BRCA1_SoF). Raw WGS data for ChIP-seq experiments are on the Sequence Read Archive (SRA: https://www.ncbi.nlm.nih.gov/sra), BioProject: PRJNA1378990; BioSamples: SAMN53860886. URL for access (no reviewer’s access code, since data has been publicly released): https://www.ncbi.nlm.nih.gov/bioproject/PRJNA1378990.

### Mouse mammary tumor models and WGS

Mouse mammary tumors were generated in *K14-Cre;Trp53*^fl2-10/fl2-10^;*Brca1*^fl5-13/fl5-13^ (B1P), *K14-Cre;Trp53*^fl2-10/fl2-10^*;Brca2*^fl11/fl11^ (B2P) and *K14-cre;Trp53*^fl2-10/fl2-10^*;Brca1*^L1363P/fl5-13^ (Br1L1363P) female mice as described previously (Jonkers et al, 2001; Liu et al, 2007; Menghi et al, 2018; Pulver et al, 2021). Genomic libraries were derived from 20 mouse tumor tissues and their matched mouse spleen tissues (normal controls), by shearing 200 ng of genomic DNA using the R230 ultrasonicator (Covaris), followed by size selection using Ampure XP Beads. DNA fragments were then treated with end-repair, A-tailing, and ligation of Illumina unique adapters (Illumina) using the KAPA Hyper Prep Kit (Illumina) (KAPA Biosystems/Roche), after which five cycles of PCR amplification were performed. Library quantification was performed using real-time qPCR (Thermo Fisher) before sequencing on a Novaseq X Plus Seq (Illumina) platform to generate 151-bp-long paired-end reads, aiming for 30X coverage. Sequencing reads were aligned to the mouse *GRCm39* reference genome using the Burrows-Wheeler Aligner (Li and Durbin, 2009).

For each cancer genome, we calculated the sequencing coverage across the *Trp53* and *Brca1* loci, extending each region by 50% of its length upstream and downstream, using non-overlapping 100-bp bins. Coverage values were then normalized across samples, using the *edgeR::calcNormFactors* function in R. A rolling average of the normalized coverage was computed for each sample, using windows of 25 and 50 consecutive 100-bp bins for *Trp53* and *Brca1*, respectively, as represented in Appendix Fig. S4B. We then averaged the values corresponding to the windows mapping to the delta region as well as the left and right flanking regions, for either gene, separately, and computed the delta allele frequency (DeltaFr) as 1− (coverage in the flanking windows/coverage in the delta windows).

Structural variant calls were generated using four different tools (Manta (Chen et al, 2016), Delly (Rausch et al, 2012), LUMPY (Layer et al, 2014), and SvABA (Wala et al, 2018), and high-confidence events were selected when called by at least two tools and by requiring split-read support. Matched normal DNA samples were used as controls to remove germline variants. A TDP score was computed for each tumor sample based on the number and chromosomal distribution of its somatic tandem duplications (TDs), and TDP Group 1 was assigned to genomes exhibiting a TD span distribution peak between 1.64 and 51 kb, as previously described (Menghi et al, 2018; Menghi et al, 2016).

Single-nucleotide variants were called by MuTect2 (preprint: Benjamin et al, 2019), Strelka2 (Kim et al, 2018), and Lancet (Narzisi et al, 2018). High-confidence calls were defined as those that are either called by at least two variant callers or called by one caller and seen in the Lancet validation calls, and that occur at a variant allele frequency of 0.07 or above. Putative germline variants were removed by only keeping mutations unique to each cancer genome. In addition to the 15 tumor genomes sequenced as part of this study, we also applied the same pipeline to reanalyze 15 previously published mouse breast cancer genomes from the following genotypes: *K14-cre;Trp53*^fl2-10/fl2-10^ (KP, *n* = 3), *WAP-Cre;Trp53*^fl2-10/fl2-10^ (WP, *n* = 3), *K14-Cre;Trp53*^fl2-10/fl2-10^*;Brca1*^fl5-13/fl5-13^ (KB1P, *n* = 3), *WAP-Cre;Trp53*^fl2-10/fl2-10^*;Brca1*^fl5-13/fl5-13^ (WB1P, *n* = 3), *K14-Cre;Trp53*^fl2-10/fl2-10^*;Brca2*^fl11/fl11^ (KB2P, *n* = 3) (Menghi et al, 2018; Menghi et al, 2016).

Single-base substitution (SBS) signatures were extracted with the *YAPSA* R package (Hübschmann et al, 2021) using the original 22 high-confidence COSMIC signatures described by Alexandrov et al as the mutational process matrix (Alexandrov et al, 2013).

WGS data relative to the new mouse breast cancers sequenced as part of this study (i.e., 20 pairs of spleen and tumor DNA samples) are available from the Sequence Read Archive database (www.ncbi.nlm.nih.gov/sra), SRA: PRJNA1393414. Previously published mouse cancer WGS datasets (Menghi et al, 2018) are also available from SRA: PRJNA430898.

## Supplementary information

Appendix

Peer Review File

Source data Fig. 1

Source data Fig. 2

Source data Fig. 3

Source data Fig. 4

Source data Fig. 6

Expanded View Figures

## Data Availability

ChIP-seq data: Analysis code is available on GitHub (https://github.com/AlbertoMarinG/BRCA1_SoF). Raw WGS data for ChIP-seq experiments are on the Sequence Read Archive (SRA: https://www.ncbi.nlm.nih.gov/sra), BioProject: PRJNA1378990; BioSamples: SAMN53860886. WGS data: WGS data relative to the new mouse mammary tumors sequenced as part of this study (i.e., 20 pairs of spleen and tumor DNA samples) are available from the Sequence Read Archive database (www.ncbi.nlm.nih.gov/sra), SRA: PRJNA1393414. The source data of this paper are collected in the following database record: biostudies:S-SCDT-10_1038-S44318-026-00913-x.
