# Supplementary material for: The BRCA1 coiled-coil domain is dispensable for suppression of tandem duplications and tolerance of FANCM loss
Source: EMBO J. 2026 Sep 18;45(20):7136–62. doi: 10.1038/s44318-026-00913-x (PMC13672311; doi:10.1038/s44318-026-00913-x)
Supplement: Supplementary file 1 [file 44318_2026_913_MOESM1_ESM.pdf]

Appendix for “The BRCA1 coiled coil domain is dispensable for tandem duplication suppression and tolerance of FANCM loss”

Appendix Figure S1	page 2
Appendix Figure S2	page 3
Appendix Figure S3	page 4
Appendix Figure S4	page 5
Appendix Figure S5	page 7
References	page 8

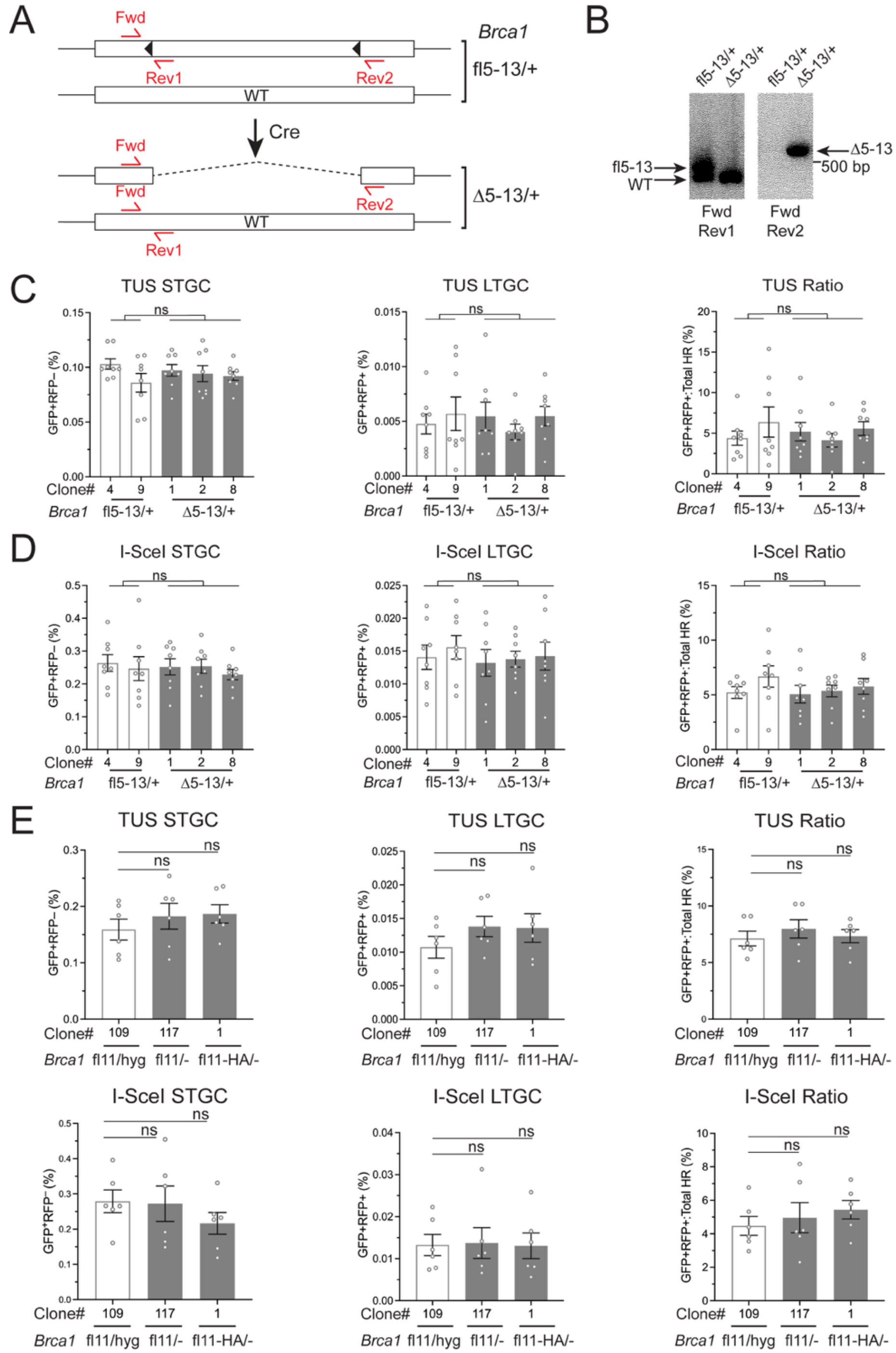

Appendix Figure S1. Quantitation of HR in *Brca1* hemizygous mES cells. **A**. Schematic of clones with one floxed *Brca1* allele, $Brca1^{fl5\text{-}13}$ and Cre-mediated deletion product $Brca1^{\Delta 5\text{-}13}$. Forward (Fwd) and reverse (Rev) primers used to detect deletion of the floxed region are shown. **B.** PCR genotyping of $Brca1^{fl5\text{-}13/+}$ and $Brca1^{\Delta 5\text{-}13/+}$ clones. **C-D.** HR repair product frequencies in three independent $Brca1^{\Delta 5\text{-}13/+}$ clones and two independent $Brca1^{fl5\text{-}13/+}$ isogenic clones. Data for Tus/*Ter*-induced (C) and I-SceI-induced (D) repair is shown. Data shows mean and s.e.m. of eight independent experiments (n=8). Statistical analysis: RM-one way ANOVA with Geisser-Greenhouse correction: ns: not significant. **E.** Tus/*Ter*-induced (upper panel) and I-SceI-induced (lower panel) HR frequencies in parental $Brca1^{fl11/hyg}$, $Brca1^{fl11/–}$ and $Brca1^{fl11\text{-}HA/–}$ are shown as mean and SEM of six independent experiments (n=6). Statistical analysis: Unpaired *t*-test with Welch's correction. ns: not significant.

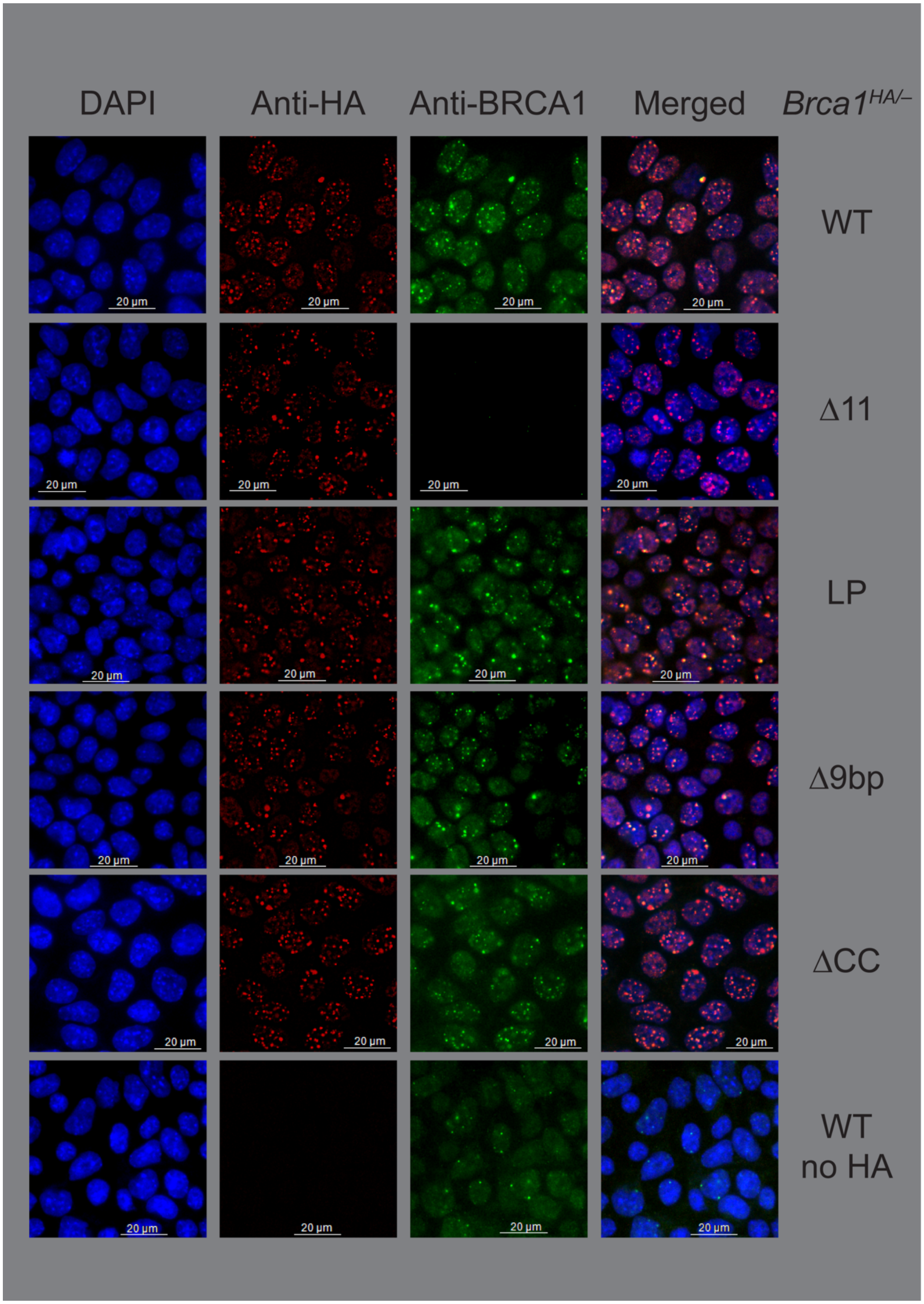

Appendix Figure S2. Nuclear localization of BRCA1-8xHA mutants. Immunofluorescence immunostaining of *Brca1*$^{HA/–}$ mES cells, acquired using 60X objective (see Methods). Alexa fluor 647 (red): anti-HA antibody signal. Alexa fluor 488 (green): signal of antibody raised against BRCA1 mouse Exon 11 (NCBI exon 10)-encoded polypeptide. DAPI: Nuclear counterstain. Note absence of red signal in *Brca1*$^{\Delta 11\text{-}HA/–}$ cells and absence of green signal in *Brca1*$^{fl11/hyg}$ ("WT no HA") cells.

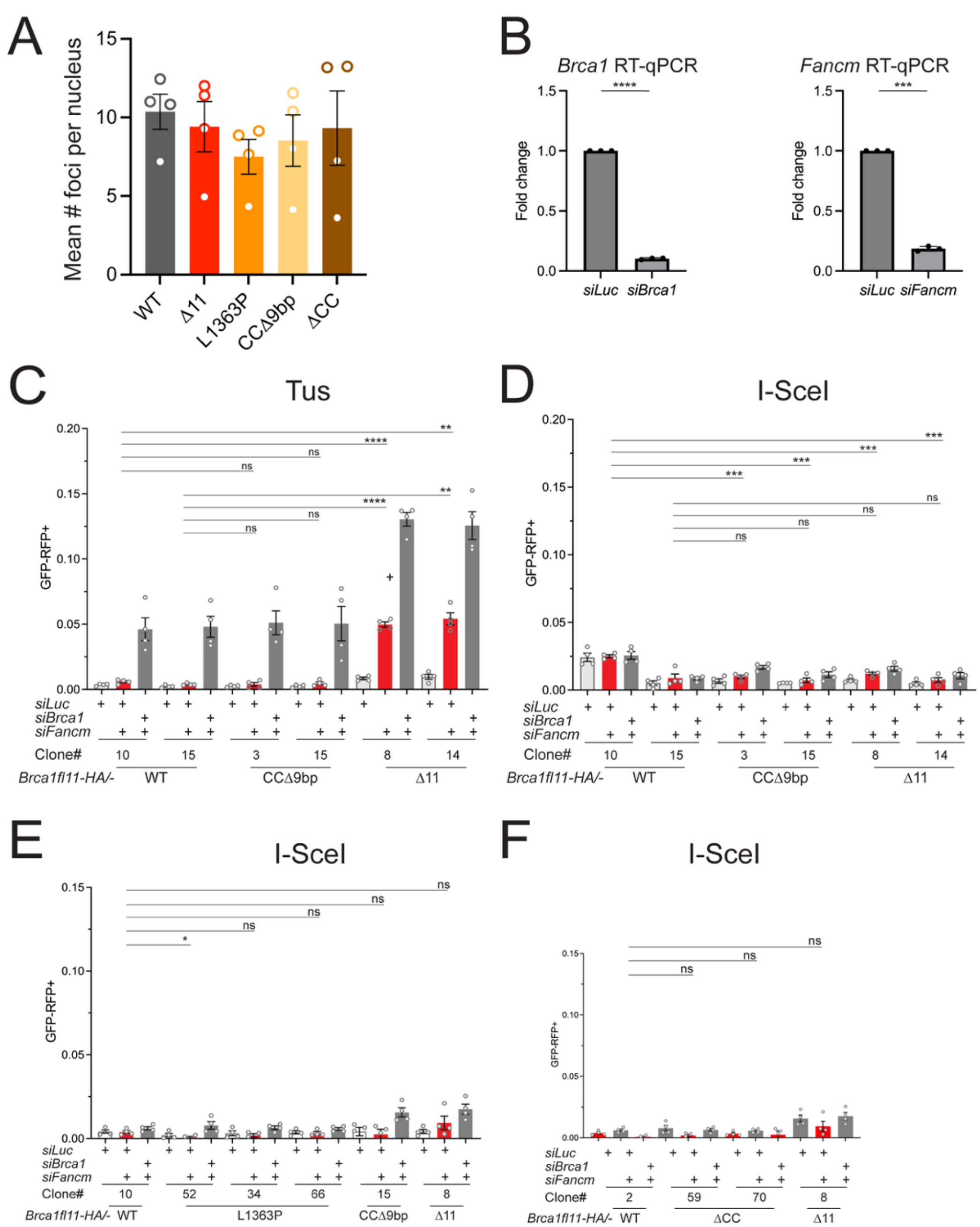

Appendix Figure S3. A. Quantitation of focus formation in cell lines shown in **Appendix Figure S2**. Foci scoring described in Methods. Data shows mean and s.e.m. of four independent experiments (n=4). Unpaired t-test with Welch's correction. ns: not significant. **B.** RT-qPCR analysis of *Brca1* and *Fancm* mRNA depletion following siRNA-mediated transfection in representative samples, related to **Figure 4** and in panels **C-F** of this Figure. Statistical analysis: Unpaired *t*-test with Welch's correction. *** $p < 0.0005$, **** $p < 0.0001$. **C-F.** Tus/*Ter*-induced (**C**) or I-SceI-induced (**D-F**) $GFP^{-}RFP^{+}$ repair products in *Brca1* WT and mutant genotypes shown, in the presence of siRNAs against *Fancm* and/or *Brca1*. *Brca1* allele abbreviations as in **Figure 1A**. See Methods for details. Data shows mean and s.e.m. of 4 independent experiments (n=4). siRNA against luciferase (si*Luc*): non-specific siRNA negative control. Gray columns: combined si*Fancm* and si*Brca1* induces TDs in all cell lines (positive control). Red columns: si*Fancm* and si*Luc* test TD suppression in the *Brca1* genotypes shown. Statistical analysis: Unpaired *t*-test with Welch's correction. ns: not significant, ** $p < 0.005$; *** $p < 0.0005$; **** $p<0.0001$.

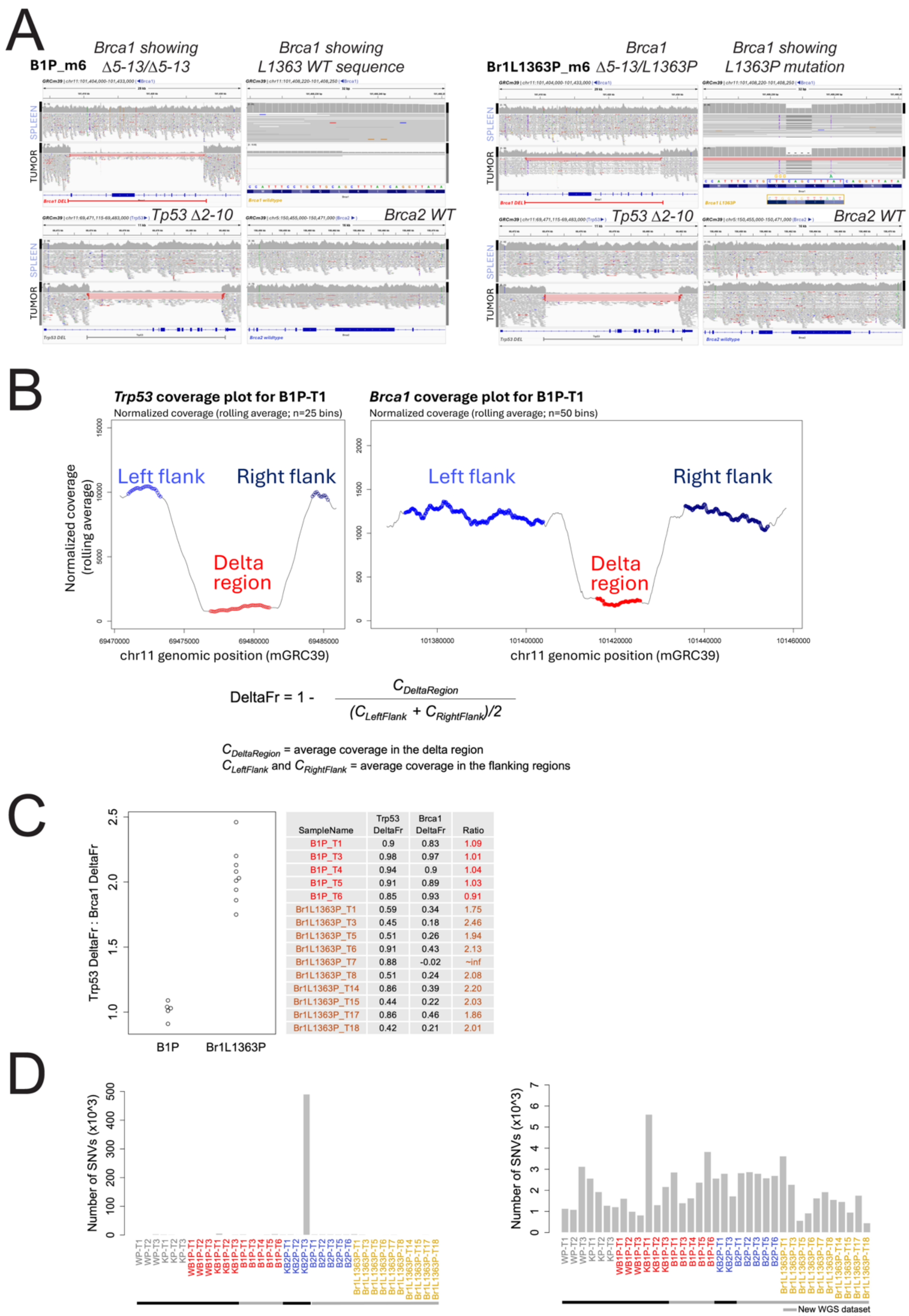

SampleName	Trp53 DeltaFr	Brca1 DeltaFr	Ratio
B1P_T1	0.9	0.83	1.09
B1P_T3	0.98	0.97	1.01
B1P_T4	0.94	0.9	1.04
B1P_T5	0.91	0.89	1.03
B1P_T6	0.85	0.93	0.91
Br1L1363P_T1	0.59	0.34	1.75
Br1L1363P_T3	0.45	0.18	2.46
Br1L1363P_T5	0.51	0.26	1.94
Br1L1363P_T6	0.91	0.43	2.13
Br1L1363P_T7	0.88	-0.02	~inf
Br1L1363P_T8	0.51	0.24	2.08
Br1L1363P_T14	0.86	0.39	2.20
Br1L1363P_T15	0.44	0.22	2.03
Br1L1363P_T17	0.86	0.46	1.86
Br1L1363P_T18	0.42	0.21	2.01

Appendix Figure S4. Analysis of conditional *Brca* mutant tumors in *K14-Cre;Trp53*$^{fl2\text{-}10/fl2\text{-}10}$ mice. **A**. Integrative Genomic Viewer (IGV) screenshots of WGS reads mapping to the targeted genomic regions in matched spleen and mammary tumor tissues from individual animals representative of *K14-Cre;Trp53*$^{fl2\text{-}10/fl2\text{-}10}$*;Brca1*$^{fl5\text{-}13/fl5\text{-}13}$ (B1P) and *K14-cre;Trp53*$^{fl2\text{-}10/fl2\text{-}10}$*;Brca1*$^{L1363P/fl5\text{-}13}$ (Br1L1363P) mouse models examined in this study. Two magnifications of Brca1 sequence are shown. The first (left panel) spans exons 4-13 to show Δ5-13 status of tumor tissue. The second (right panel) shows the

sequence at codon 1363, showing L1363P mutation in Br1L1363P tumor tissue. For the *Brca1* p.L1363P mutation, annotations have been added to clarify the nucleotide and amino-acid sequences corresponding to both the wild type and mutant *Brca1* alleles. For each screenshot, coverage and alignment tracks are highlighted by black and gray vertical bars, respectively. Bars and associated annotations below the gene structure track identify the exact genomic coordinates for the detected deletions. Note that since the *Brca1* gene is encoded on the negative strand, the reported nucleotide sequence corresponds to the reverse and complement of the actual coding sequence. **B**. Examples of coverage plots for *Trp53* and *Brca1* targeted regions, showing marked drop in coverage for the genomic segments corresponding to the Cre-induced deletions (Delta region in red) compared to the left and right flanking regions (light and dark blue, respectively). Degree of deletion is calculated as the 'DeltaFr' using the formula shown (see Methods). **C.** The *Trp53*-DeltaFr:*Brca1*-DeltaFr ratio was computed to confirm the genotypes of the Br1L1363P and B1P tumors. Computed as 1– the average ratio between the coverage of the flanking region and that of the delta region, DeltaFr here indicates the extent of the loss of coverage associated with the Cre-induced deletion of the target regions. A DeltaFr = 1 corresponds to a decrease in coverage down to 0; a DeltaFr = 0 corresponds to equal coverage between flanking and delta regions. As expected for tumors homozygous for the *Trp53*$^{\Delta 2\text{-}10}$ allele and heterozygous for the *Brca1*$^{\Delta 5\text{-}13}$ allele, Br1L1363P tumors have a *Trp53*-DeltaFr:*Brca1*-DeltaFr ratio of about 2. In contrast, in the B1P tumors, which are homozygous for both the *Trp53*$^{\Delta 2\text{-}10}$ and *Brca1*$^{\Delta 5\text{-}13}$ alleles, the *Trp53*-DeltaFr:*Brca1*-DeltaFr ratio = ~1. The only exception to this pattern is the Br1L1363P tumor 7, which contained an outsized deletion of *Brca1* that included the defined flanking regions and hence could not be plotted since the *Trp53*-DeltaFr:*Brca1*-DeltaFr ratio is infinite. **D.** Numbers of somatic single nucleotide variants (SNV) per tumor sample across the 15 mouse cancer genomes previously published (Menghi *et al*, 2018) and the new mouse cancer genomes sequenced as part of this study, including (left) or excluding (right) the B2P-T2 outlier sample.

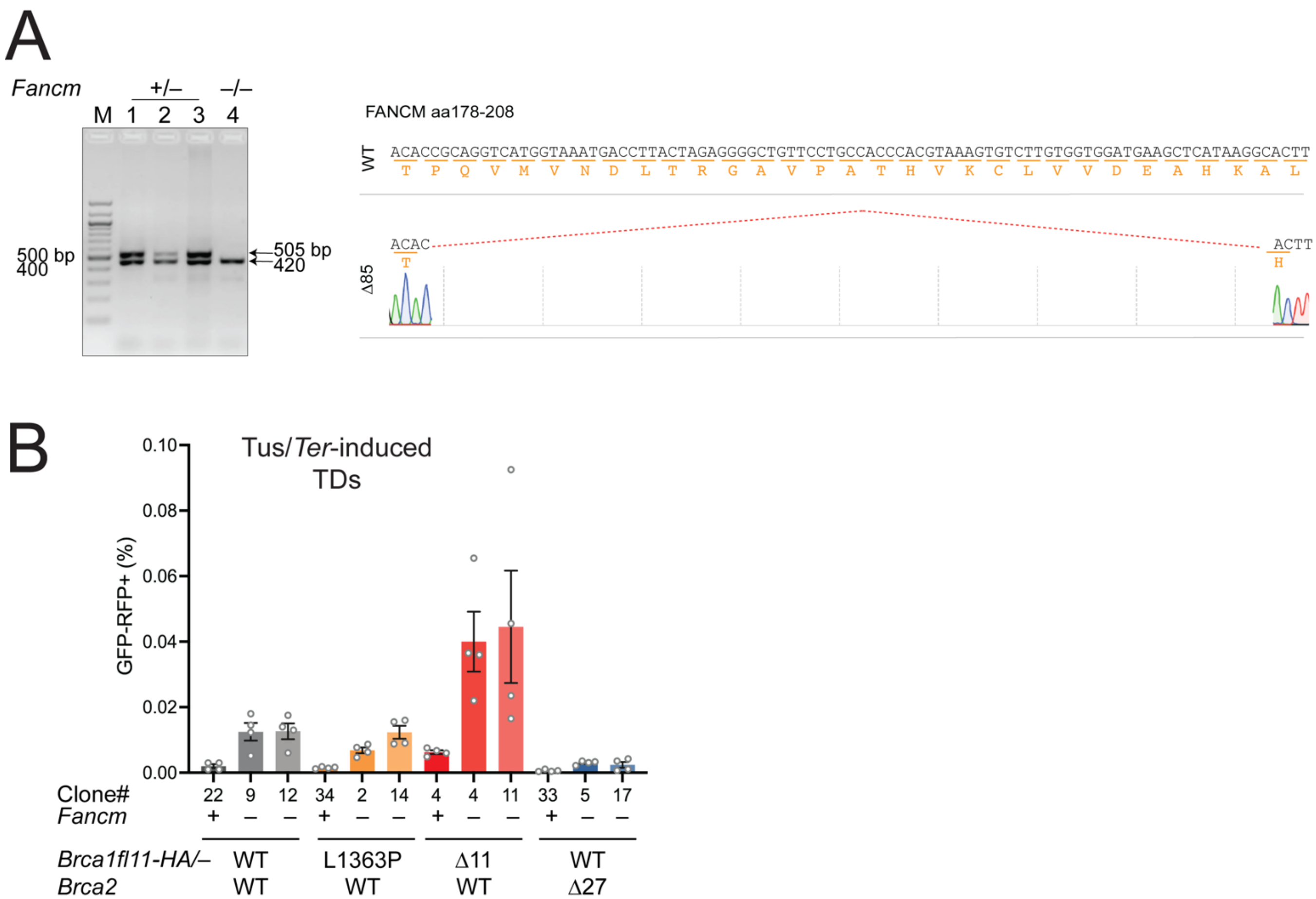

Appendix Figure S5. Tus/*Ter*-induced TD formation in *Fancm*$^{-/-}$ derivatives of *Brca* mutant cell lines. A. Genotyping of *Fancm*$^{+/-}$ and *Fancm*$^{-/-}$ clones. Left panel: PCR analysis of three *Fancm*$^{+/-}$ clones and one *Fancm*$^{-/-}$ clone. Right: sequence of *Fancm* Δ85 allele. This allele was shown previously to be functionally null (Panday *et al*, 2021). **B.** Tus/*Ter*-induced TD formation in different *Fancm*$^{-/-}$ *Brca* mutant clones. Note induction of TDs in *Fancm*$^{-/-}$ *Brca1*$^{\Delta 11\text{-HA}/-}$ clones and basal (WT) TD levels in other genotypes.

References

Menghi F, Barthel FP, Yadav V, Tang M, Ji B, Tang Z, Carter GW, Ruan Y, Scully R, Verhaak RGW *et al* (2018) The Tandem Duplicator Phenotype Is a Prevalent Genome-Wide Cancer Configuration Driven by Distinct Gene Mutations. *Cancer Cell* 34: 197–210 e195

Panday A, Willis NA, Elango R, Menghi F, Duffey EE, Liu ET, Scully R (2021) FANCM regulates repair pathway choice at stalled replication forks. *Mol Cell* 81: 2428–2444.e2426
