# Supplementary material for: The BRCA1 coiled-coil domain is dispensable for suppression of tandem duplications and tolerance of FANCM loss
Source: EMBO J. 2026 Sep 18;45(20):7136–62. doi: 10.1038/s44318-026-00913-x (PMC13672311; doi:10.1038/s44318-026-00913-x)
Supplement: Supplementary file 2 [file 44318_2026_913_MOESM2_ESM.pdf]

THE EMBO JOURNAL

The BRCA1 coiled-coil domain is dispensable for suppression of tandem duplications and tolerance of FANCM loss

Namrata Nilavar, Alberto Marin Gonzalez, Francesca Menghi, Daniel Nguyen, Nicholas Willis, Ellen Wientjens, Bing Xia, Jos Jonkers, Edison Liu, and Ralph Scully

Corresponding author(s): Ralph Scully (rscully@bidmc.harvard.edu)

Review Timeline:

Submission Date:	18th Feb 26
Editorial Decision:	27th Mar 26
Revision Received:	29th Jul 26
Editorial Decision:	25th Aug 26
Revision Received:	5th Sep 26
Accepted:	7th Sep 26

Editor: Hartmut Vodermaier

Transaction Report:

(Note: With the exception of the correction of typographical or spelling errors that could be a source of ambiguity, letters and reports are not edited. Depending on transfer agreements, referee reports obtained elsewhere may or may not be included in this compilation. Referee reports are anonymous unless the Referee chooses to sign their reports.)

1st Editorial Decision 27th Mar 2026

Dr. Ralph Scully
Beth Israel Deaconess Medical Cntr
Medicine, Division of Hematology
330 Brookline Avenue, CLS-438
Boston, MA 02215

27th Mar 2026

Re: EMBOJ-2026-123909-T
Defective BRCA1-mediated DNA end resection drives tandem duplication formation and FANCM synthetic lethality

Dear Ralph,

Thank you for submitting your manuscript on distinct BRCA1 mutants and the differential roles of specific BRCA1 functions for our consideration. We have now received feedback from three expert referees, copied below for your information. As you will see, the referees express some interest in your findings, but also raise a number of substantive concerns with the study in its current state. One key issue is that referee 3 finds it difficult to argue that the Δexon11 mutant is a straightforward separation-of-function mutant, given that its loss of an NLS also partly relocalizes it to the cytoplasm. In addition, all referees have various more specific points that would need to be satisfactorily addressed prior to publication.

After follow-up discussions with the referee panel, we decided that the study would warrant EMBO Journal publication despite referee 3's main concern, as long as the respective conclusions would be adequately modified to avoid over/mis-interpretation. I would therefore invite you to revise the work in response to the three reports, and resubmit a modified version via the link below. Please note that it is our policy to consider only a single round of major revision, making it important to comprehensively respond to each referee point at the time of resubmission; therefore, please do not hesitate to get back to me in case you would like to clarify/discuss any of the referees' points or plans for addressing them ahead of time. We would also be open to extending the revision deadline if that should be helpful. As always, our scooping protection will remain valid throughout the full revision period.

Detailed information on preparing, formatting and uploading a revised manuscript can be found below and in our Guide to Authors, and adhering to them as closely as possible shall greatly facilitate editorial processing upon resubmission. Thank you again for the opportunity to consider this work for The EMBO Journal, and I look forward to your revision in due time.

With kind regards,

Hartmut

Hartmut Vodermaier, PhD
Senior Editor, The EMBO Journal
h.vodermaier@embojournal.org

*** PLEASE NOTE: All revised manuscript are subject to initial checks for completeness and adherence to our formatting guidelines. Revisions may be returned to the authors and delayed in their editorial re-evaluation if they fail to comply to the following requirements. As a first step please read our guidelines for revised submissions:
https://link.springer.com/journal/44318/submission-guidelines#cms-Revised-submissions

1) Every manuscript requires a Data Availability section (even if only stating that no deposited datasets are included). Primary datasets or computer code produced in the current study have to be deposited in appropriate public repositories prior to resubmission, and reviewer access details provided in case that public access is not yet allowed.

2) Each figure legend must specify
- size of the scale bars that are mandatory for all micrograph panels
- the statistical test used to generate error bars and P-values
- the type error bars (e.g., S.E.M., S.D.)
- the number (n) and nature (biological or technical replicate) of independent experiments underlying each data point
- Figures may not include error bars for experiments with n<3; scatter plots showing individual data points should be used instead.

3) Revised manuscript text (including main tables, and figure legends for main and EV figures) has to be submitted as editable text file (e.g., .docx format). We encourage highlighting of changes (e.g., via text color) for the referees' reference.

4) Each main and each Expanded View (EV) figure should be uploaded as individual production-quality files (preferably in .eps, .tif, .jpg formats). For suggestions on figure preparation/layout, please refer to our Figure Preparation Guidelines: https://media.springernature.com/original/springer-cms/rest/v1/content/27825798/data/v1

5) Point-by-point response letters should include the original referee comments in full together with your detailed responses to them (and to specific editor requests if applicable), and also be uploaded as editable (e.g., .docx) text files.

6) Please complete our Author Checklist, and make sure that information entered into the checklist is also reflected in the manuscript; the checklist will be available to readers as part of the Review Process File.

7) All authors listed as (co-)corresponding need to deposit, in their respective author profiles in our submission system, a unique ORCiD identifier linked to their name. Please see our Guide to Authors for detailed instructions.

8) Please note that supplementary information at EMBO Press has been superseded by the 'Expanded View' for inclusion of additional figures, tables, movies or datasets; with up to five EV Figures being typeset and directly accessible in the HTML version of the article.

9) To facilitate reproducibilty and cross-laboratory adoption of methodologies, please structure the Materials & Methods section as outlined in our guide to authors, including a completed Reagents and Tools Table.

10) Digital image enhancement is acceptable practice, as long as it accurately represents the original data and conforms to community standards. If a figure has been subjected to significant electronic manipulation, this must be clearly noted in the figure legend and/or the 'Materials and Methods' section. The editors reserve the right to request original versions of figures and the original images that were used to assemble the figure. Finally, we generally encourage uploading of numerical as well as gel/blot image source data.

At EMBO Press we ask authors to provide source data for the main manuscript figures. You will receive a separate email with instructions for providing source data with your revised manuscript, including how to upload and organize the files.

In the interest of ensuring the conceptual advance provided by the work, we recommend submitting a revision within 3 months (25th Jun 2026). Please discuss the revision progress ahead of this time with the editor if you require more time to complete the revisions. Use the link below to submit your revision:

Link Not Available

Referee #1:

The authors investigate which function of BRCA1 is critical for suppressing tandem duplication (TD) formation and synthetic lethality associated with FANCM loss. They generated a series of BRCA1 separation-of-function mutants in mouse embryonic stem (mES) cells, allowing them to dissect the distinct roles of BRCA1. Their results suggest that the end resection function, rather than the homologous recombination (HR) function of BRCA1, is essential for suppressing TD formation and the lethality observed upon FANCM loss. This finding is informative to the field, although conceptual advance is somewhat limited by the group's previous work that showed BARD1 and CtIP are major suppressors of Tus-Ter-induced TD formation (PMID: 29168504). The manuscript could be better organized, and some data presentation could also be improved. Nonetheless, it may be suitable for publication in EMBO Journal if the following issues are addressed:

1. Figures 1 and 2, along with their corresponding text, primarily present technical validation and methodological details. These data would be more appropriate for the supplementary figures and Methods section.
2. The authors demonstrate defects in the interaction between BRCA1 mutants and PALB2 but do not discuss how this observation relates to the main conclusions of the study. This point should be clarified.
3. In Figures 3 and S4, the authors should explain how the BRCA1Δ11 mutant shows a stronger defect in I-SceI-induced STGC but not in Tus-Ter-induced STGC.
4. In Figure 4, it is unnecessary to show all individual replicates. Panels 4B and 4C could be combined into a single panel for clarity. In addition, a third biological replicate should be included.
5. In Figure 5, the GFP−RFP+ percentages differ among the siBRCA1 + siFANCM samples. If these differences are due to clonal variation, at least two independent clones per genotype should be analyzed.
6. In Figure 6C, defining one sample as an outlier based on only five samples is not statistically appropriate. Increasing the sample size would strengthen the analysis.
7. In Figure 7A, the FANCM knockout clones should be clearly labeled. Additionally, FANCMKO/BRCA1WT #2 appears similar

to FANCMKO/BRCA2Δ27/Δ27#2, yet the quantification in Figure 7B shows a large difference. This discrepancy should be explained.
8. In Figure S5A, labeling two regions identically as "Brca1" is confusing. These should be renamed, for example, as "Brca1 region #1" and "Brca1 region #2."
9. In Figure S5B, the figure does not clearly illustrate how the "DeltaFr" value is calculated. Additional explanation or schematic guidance is needed.
10. Line 296: This paragraph lacks a clear conclusion. The authors should explicitly state what the DeltaFr values indicate (e.g., efficient Cre-mediated recombination).
11. Line 299: The criteria for defining TDP Group 1 ({less than or equal to}10 kb) should be clearly described in the main text, even though briefly mentioned in the Introduction.
12. Lines 328-334: Technical details such as performing two rounds of transfection to obtain clean FANCM knockout clones should be moved to the Methods section.
13. In Figure 6A, at least one additional mutation in the CC domain should be analyzed to strengthen the conclusions.
14. Fig S7 points to a BRCA1-dependent SSA model to explain their observations, though this is not mechanistically tested. It would be valuable to refine this point by making sure other SSA mediators, e.g. RAD52, are dispensable, and that this is RAD51-independent. If RAD52 KO however does partially phenocopy BRCA1del11 for TD formation, this would also strengthen the SSA hypothesis (though the two do not necessarily have to overlap).
15. The final model in FigS7 is a helpful illustration but since there are multiple genotypic combinations testing multiple phenotypes across the main figures, adding a matrix summary (BRCA1 genotype vs. measured phenotype i.e. TD suppression, end-resection, DSB HR, vaibility) with a brief explanation of the effect under FANCM WT vs -/- may work better. This would provide a clearer conceptual understanding to the reader for the manuscript's conclusions and findings.

Referee #2:

The authors generated a haploid genetic system in mouse embryonic stem cells to analyze Brca1 function in homologous recombination (HR) and stalled replication fork repair. They show that BRCA1 coiled-coil (CC) domain mutants that disrupt PALB2 interaction are defective in HR but retain normal DNA end resection. Despite reduced HR and increased PARP inhibitor sensitivity, these mutants still suppress replication-fork-associated tandem duplications (TDs) and tolerate FANCM loss, unlike the resection-defective Brca1Δ11 mutant. Further, mouse tumors driven by Brca1 L1363P lack the BRCA1-deficiency TD signature. Overall, the study demonstrates that BRCA1-mediated TD suppression and FANCM tolerance depend on DNA end resection rather than PALB2-dependent HR, revealing a separation of BRCA1 functions in the maintenance of genome stability. The study provides a significant advance in understanding related BRCA1-dependent processes of DNA damage repair and replication fork maintenance. The work, which has been performed very well, is certainly of general interest. A few relatively minor concerns need to be addressed though.

Specific Comments

1. The study assumes that Brca1+/- mES cells are not haplo-insufficient based on the HR reporter assays which may not the case with human cells. Comment on this at least.

2. Please comment on why the del CC has increased BRCA1/HA foci. Please quantify the data in Fig. S3.

3. How would you explain sensitivity to Olaparib between del9 del cc and L1363P based on Fig 2C and BRCA1 foci formation?

4. The analysis includes five tumors per genotype. Is this sample size sufficient to confidently conclude that Brca1L1363P tumors lack Group 1 TDs?

5. The Ter-HR reporter is integrated at the Rosa26 locus. Could chromatin context at Rosa26 influence HR outcomes compared with endogenous loci? Please discuss this.

6. FANCM depletion is used to induce TDs. Do authors predict that other fork remodeling enzymes (SMARCAL1, ZRANB3, HLTF) would produce similar outcomes?

7. Are there enough data points in Fig. 4C to be confident in the statistics?

Referee #3:

The presence of 10 kb tandem duplications in the genome is a characteristic feature of BRCA1- but not BRCA2-deficient cancers. The authors have previously shown that FANCM deficiency also increases tandem duplications, and that loss of both

BRCA1 and FANCM is synthetic lethal. In this manuscript, the authors investigate whether the interaction of BRCA1 with PALB2 is required for suppression of tandem duplications, using a set of BRCA1 coiled-coil domain mutants that abolish PALB2 binding. They find that these mutants are deficient in homologous recombination (HR), but their expression does not cause tandem duplications nor cell death due to loss of FANCM. In contrast, expression of a BRCA1 "exon 11" mutant that is defective in HR due to a large deletion in the central region of BRCA1, does lead to tandem duplications and causes synthetic lethality with FANCM loss. The authors conclude from this that it is the role of BRCA1 in DNA end resection that suppresses tandem duplications rather than its role in loading RAD51 with PALB2-BRCA2.

The manuscript addresses an important question and presents interesting genetic observations. However, the mechanistic interpretation that DNA end resection specifically suppresses tandem duplication formation and determines FANCM synthetic lethality, is not sufficiently supported by the current data. The BRCA1 exon 11 mutant is not a separation of function mutant, it is defective in multiple functions of BRCA1 (including RAD51 recruitment and HR, as the authors themselves and others have shown) and is partly cytoplasmic due to loss of NLS. The BRCA1-BARD1 complex has many previously described roles in response to DNA damage. The authors therefore cannot conclude that a defect in DNA end resection has anything to do with tandem duplication formation. While they do formally acknowledge this in the Discussion, they definitively state that DNA resection defects are driving TD formation in the title, abstract and text of the manuscript, which they have not established. The manuscript therefore needs substantial revision to take this into account before it should be published. At a minimum, the authors should significantly moderate the mechanistic claim regarding resection, and reframe the study as demonstrating that BRCA1 suppression of TD formation and tolerance to FANCM loss does not require the BRCA1-PALB2 interaction or canonical HR.

Main points:

1. Without a BRCA1 mutant that exclusively abolishes DNA end resection, it remains possible that other unidentified functions of BRCA1, which are also affected by exon 11 deletion, could contribute to tandem duplications and synthetic lethality with FANCM. Further studies with more targeted mutations would be needed to conclusively determine the specific role of DNA end resection in this context. For example, the E3 ligase activity of BRCA1-BARD1 has been shown to ubiquitylate histones and promote resection by some groups (Densham et al., NSMB 2016; Wang et al., Mol. Cell 2023), but not others (Nakamura et al., NCB 2019). How does a RING mutation (that maintains BRCA1-BARD1 interaction but abolishes E3 ligase activity) behave in the authors' resection, FANCM synthetic lethality and TD formation assays? Similarly, BARD1 was recently shown to interact with SLX4-XPF (Tsukada et al., Mol. Cell 2024), which the authors themselves have previously linked to regulating Tus/Ter HR (Elango et al., NSMB 2022). How does mutating that motif in BARD1 affect the same assays? Examining these mutants may provide the necessary evidence to prove or disprove the authors hypothesis that BRCA1's role in resection is what is driving tandem duplications and FANCM synthetic lethality.

2. Fig. 4: the RPA ChIP experiment has only two replicates, two additional ones are needed. There may be a defect with some of the coiled-coiled mutants, but the difference between the current replicates is too great to know for sure.

3. What happens in the authors' resection assay (Fig. 4) when the exon 11 mutant cells are treated with siBRCA1? The BRCA1 BRCTs bind to the RAP80 complex, which suppresses resection. Could the mutant BRCA1 protein be acting in a dominant negative manner to suppress resection?

Other points:

1. The authors state in the abstract that TDs are drivers of tumorigenesis in BRCA1-deficient cancers, but how do they know this without a separation-of function allele? Surely it is more likely that loss of HR drives tumorigenesis, given that BRCA2, PALB2 and RAD51 paralogues are also breast and ovarian tumour suppressors but they don't suppress TDs?

2. There is significant disagreement in the literature about whether BRCA1 promotes resection at all DSBs (e.g. Densham et al., NSMB 2016), or promotes resection at IR-induced DSBs but not broken replication forks (Pavani et al., Science 2024), or actually inhibits resection (e.g. Dever et al., Aging 2011; Polato et al., J. Exp. Med. 2014), or if BRCA1 has only a minor or no direct role in the process at all (e.g. Escribano-Diaz et al., Mol. Cell 2012; Reczek et al, JCB 2013; Cruz-García et al., Cell Rep. 2014; Ochs et al., NSMB 2016). BRCA1 deficient cells are usually unhealthy. Could the apparent resection defects seen by some groups but not others, be down to more G1 cells and/or the general health of the cells, rather than any direct role of BRCA1 in the process? A more balanced discussion of the disagreements in the literature would be beneficial.

3. The exact boundaries of the exon-11 deletion and other mutants should be clearly defined (amino acid numbers), and the corresponding human residues should be provided in the manuscript.

Response to referees

We thank the referees for their helpful and constructive critiques. We have responded to the points raised by the referees in blue font (below). In addition to the requested revisions, we have included a new **Extended View Figure EV4**, in which we performed competitive growth assays for each *Brca* mutant cell line but on a *Fancm* WT background. The data serves as an additional control for **Figures 6C** and **6D** and demonstrates that, while *Fancm* deletion severely reduces growth of *Brca1* Δ11 cells, *Fancm* deletion modestly *improves* the growth of *Brca1* coiled coil domain mutant cells and of *Brca2* mutant cells, in comparison to *Brca* WT;*Fancm*$^{-/-}$ cells. In the resubmitted m/s, we reference a m/s from Dossin et al. (currently on BioRxiv) that documents similar patterns of *Fancm*-dependent alterations in growth properties of different *Brca* mutants.

Referee #1:

The authors investigate which function of BRCA1 is critical for suppressing tandem duplication (TD) formation and synthetic lethality associated with FANCM loss. They generated a series of BRCA1 separation-of-function mutants in mouse embryonic stem (mES) cells, allowing them to dissect the distinct roles of BRCA1. Their results suggest that the end resection function, rather than the homologous recombination (HR) function of BRCA1, is essential for suppressing TD formation and the lethality observed upon FANCM loss. This finding is informative to the field, although conceptual advance is somewhat limited by the group's previous work that showed BARD1 and CtIP are major suppressors of Tus-Ter-induced TD formation (PMID: 29168504). The manuscript could be better organized, and some data presentation could also be improved. Nonetheless, it may be suitable for publication in EMBO Journal if the following issues are addressed:

1. Figures 1 and 2, along with their corresponding text, primarily present technical validation and methodological details. These data would be more appropriate for the supplementary figures and Methods section.
We have moved the original **Figure 1** into Expanded View (EV) Figures in the revised m/s (**Fig. EV1**). The original **Figure 2** describes the *Brca1* mutants studied in the m/s and is referenced as a key to the different *Brca1* alleles studied in numerous later figures. We therefore feel that it is best to preserve it as a manuscript figure (now **Figure 1**).

2. The authors demonstrate defects in the interaction between BRCA1 mutants and PALB2 but do not discuss how this observation relates to the main conclusions of the study. This point should be clarified.
We have included a fuller discussion of the significance of the PALB2 binding and associated RAD51 loading defects in the text. The importance of the PALB2-binding BRCA1 coiled coil domain is explained in the Abstract, Introduction, Results and Discussion. For example, the Discussion begins: “Here we compare two classes of *Brca1* mutant alleles in stalled-fork HR and in Group 1 TD formation. BRCA1 CC mutant proteins are defective for interaction with PALB2, leading to a defect in RAD51-

mediated HR (Anantha *et al*, 2017; Dray *et al*, 2010; Sy *et al*, 2009; Xia *et al*, 2006; Zhang *et al*, 2009). However, unlike the majority of cancer-associated *BRCA1* mutants, BRCA1 CC mutants remain competent for DNA end resection because they retain the key BRCA1 domains (RING, tandem BRCT and exon 11-encoded region) required for this function (Ceppi *et al*, 2024; Salunkhe *et al*, 2024)." We have also included a new Table 1 that summarizes how different *Brca* alleles studied here affect: DNA end resection, DSB-induced HR; Group 1 TD suppression; and impact of *Fancm* deletion on cell growth/synthetic lethality.

3. In Figures 3 and S4, the authors should explain how the BRCA1Δ11 mutant shows a stronger defect in I-SceI-induced STGC but not in Tus-Ter-induced STGC.
Thanks for emphasizing this important observation. Although we did address this difference in the Discussion, we have now included an additional commentary in the results section.

4. In Figure 4, it is unnecessary to show all individual replicates. Panels 4B and 4C could be combined into a single panel for clarity. In addition, a third biological replicate should be included.
We have performed a third biological replicate of this experiment and now show only one of the replicates directly, together with statistical analysis of the three replicates.

5. In Figure 5, the GFP−RFP+ percentages differ among the siBRCA1 + siFANCM samples. If these differences are due to clonal variation, at least two independent clones per genotype should be analyzed.
We now include the original data (now **Figure 4**) and an additional analysis of two independent clones each of *Brca1* WT, ∆9bp and ∆11 (now **Appendix Figure S3C** and **S3D**).

6. In Figure 6C, defining one sample as an outlier based on only five samples is not statistically appropriate. Increasing the sample size would strengthen the analysis.
We originally provided statistical analysis of the five *Brca2* mutant tumor samples with (n=5) or without (n=4) the apparent 'outlier'. The statistical analysis was highly significant in each case. We now only show the analysis using all five *Brca2* mutant tumor samples (n=5). In addition, we have analyzed five additional *Brca1* L1363P mutant cancers to bring the total number of analyzed tumor genomes of this genotype to n=10. All 10 fail to score as TD phenotype, further emphasizing the competence of the *Brca1* L1363P allele to suppress Group 1 TD formation. The expanded sample analysis is now presented in **Figure 5** and **Appendix Figure S4**.

We would also like to draw the reviewer's attention to a previous publication in which we performed similar analyses on 3 *Brca1* null cancers that retained WT *Brca2* and a total of 5 *Brca2* mutant cancers that retained WT *Brca1* (Menghi et al, 2018). All three *Brca1* null tumors were TDP Group 1 and all five *Brca2* mutant tumors failed to score as TDP.

7. In Figure 7A, the FANCM knockout clones should be clearly labeled. Additionally, FANCMKO/BRCA1WT #2 appears similar to FANCMKO/BRCA2Δ27/Δ27#2, yet the

quantification in Figure 7B shows a large difference. This discrepancy should be explained.
We now show more representative images of the colony formation assays (now **Figure 6A**, quantified in **Figure 6B**). All clones are labeled using keys on the top and left side of the colony formation panel.

8. In Figure S5A, labeling two regions identically as "Brca1" is confusing. These should be renamed, for example, as "Brca1 region #1" and "Brca1 region #2."
This is now **Appendix Figure S4A**. The left panel shows sequence coverage of *Brca1* exons 5-13 (i.e., the ‘floxed’ region). The right panel shows sequence across L1363 codon, to show the L1363P mutation in the Br1L1363P tumor. These panels are now labeled precisely.

9. In Figure S5B, the figure does not clearly illustrate how the "DeltaFr" value is calculated. Additional explanation or schematic guidance is needed.
We have added the DeltaFr formula to this figure (now **Appendix Figure S4B**).

10. Line 296: This paragraph lacks a clear conclusion. The authors should explicitly state what the DeltaFr values indicate (e.g., efficient Cre-mediated recombination).
This conclusion is now provided.

11. Line 299: The criteria for defining TDP Group 1 (less than or equal to 10 kb) should be clearly described in the main text, even though briefly mentioned in the Introduction.
We now reference the TDP Group 1 criteria in the Results section by referencing the original 2016 paper in which the TDP score was first defined (Menghi *et al*, 2016). In the Methods, we state: “TDP Group 1 was assigned to genomes exhibiting a TD span distribution peak between 1.64 and 51 kb, as previously described (Menghi *et al.*, 2018; Menghi *et al.*, 2016).”

12. Lines 328-334: Technical details such as performing two rounds of transfection to obtain clean FANCM knockout clones should be moved to the Methods section.
We have moved these descriptions to the Methods section as suggested.

13. In Figure 6A, at least one additional mutation in the CC domain should be analyzed to strengthen the conclusions.
There is no second mouse model of a *Brca1* CC domain mutation used to drive conditional *Brca1*-linked mammary tumorigenesis available at this time. We have noted this in the text (lines 290-291).

14. Fig S7 points to a BRCA1-dependent SSA model to explain their observations, though this is not mechanistically tested. It would be valuable to refine this point by making sure other SSA mediators, e.g. RAD52, are dispensable, and that this is RAD51-independent. If RAD52 KO however does partially phenocopy BRCA1del11 for TD formation, this would also strengthen the SSA hypothesis (though the two do not necessarily have to overlap).

We showed previously that Tus/*Ter*-induced TD formation in Fancm-depleted *Brca1* Δ11 cells is unaffected by siRNA-mediated depletion of RAD51 (PMID: 29168504). We also showed previously that Tus/*Ter*-induced TD formation is unaffected by *Brca2* mutation (PMID: 29168504). Note that *RAD51* null vertebrate cells cannot be propagated (PMID: 9430650). This fact limits our ability to categorically verify that TD formation is *RAD51*-independent. However, this is our current provisional conclusion.

Our unpublished data shows that *Rad52* deletion does not abolish Tus/*Ter*-induced TD formation. *Rad52* deletion reduces, but does not abolish SSA in mouse ES cells (our unpublished data). We are preparing a m/s describing these findings. The original **Figure S7** is now presented as **Figure EV5**. In the text of the resubmitted m/s, we emphasize that the SSA hypothesis is consistent with but not proven by our data (Lines 531-532).

15. The final model in FigS7 is a helpful illustration but since there are multiple genotypic combinations testing multiple phenotypes across the main figures, adding a matrix summary (BRCA1 genotype vs. measured phenotype i.e. TD suppression, end-resection, DSB HR, viability) with a brief explanation of the effect under FANCM WT vs -/- may work better. This would provide a clearer conceptual understanding to the reader for the manuscript's conclusions and findings.
Thanks for this suggestion. We have included a summary table (**Table 1**) as suggested.

Referee #2:

The authors generated a haploid genetic system in mouse embryonic stem cells to analyze Brca1 function in homologous recombination (HR) and stalled replication fork repair. They show that BRCA1 coiled-coil (CC) domain mutants that disrupt PALB2 interaction are defective in HR but retain normal DNA end resection. Despite reduced HR and increased PARP inhibitor sensitivity, these mutants still suppress replication-fork-associated tandem duplications (TDs) and tolerate FANCM loss, unlike the resection-defective Brca1Δ11 mutant. Further, mouse tumors driven by Brca1 L1363P lack the BRCA1-deficiency TD signature. Overall, the study demonstrates that BRCA1-mediated TD suppression and FANCM tolerance depend on DNA end resection rather than PALB2-dependent HR, revealing a separation of BRCA1 functions in the maintenance of genome stability.
The study provides a significant advance in understanding related BRCA1-dependent processes of DNA damage repair and replication fork maintenance. The work, which has been performed very well, is certainly of general interest. A few relatively minor concerns need to be addressed though.

Specific Comments

1. The study assumes that Brca1+/- mES cells are not haplo-insufficient based on the HR reporter assays which may not the case with human cells. Comment on this at least.

We have added in the text that our data on haploinsufficiency relates only to mES cells and to the specific repair functions studied.

2. Please comment on why the del CC has increased BRCA1/HA foci. Please quantify the data in Fig. S3.
The field shown of *Brca1* ∆CC cells in the original Fig S3 was not representative. (It can be seen that the major cluster of cells imaged in this panel are smaller than their counterparts in other panels.) We now provide more representative images as **Appendix Figure S2**. We have quantified anti-HA stained focus formation as requested, using four independent biological replicates (n=4) and find no significant different in anti-HA foci between the different *Brca1* genotypes. This quantitation is provided as a new panel in **Appendix Figure S3A**.

3. How would you explain sensitivity to Olaparib between del9 del cc and L1363P based on Fig 2C and BRCA1 foci formation?
The *Brca1* CC mutants encode a gene product that localizes normally (hence, they form characteristic BRCA1 foci). They are HR-defective because of a defect in binding PALB2 and, hence, coordination with BRCA2/RAD51 is impaired, resulting in a RAD51 loading defect. This idea is well established in the literature (references included in the m/s text).

With regard to Olaparib sensitivity, we note that the Brca1 ∆CC mutants are more sensitive than the *Brca1* ∆9bp or L1363P mutants. Although all *Brca1* CC mutants reveal diminished PALB2 binding, the *Brca1* ∆CC mutant may have more severe impairment of PALB2 binding.

4. The analysis includes five tumors per genotype. Is this sample size sufficient to confidently conclude that Brca1L1363P tumors lack Group 1 TDs?
Thanks for raising this concern. In our expanded mouse cancer genome dataset, combining WGS data from Menghi et al., 2018 and this manuscript, B1P and non-B1P (i.e., P53 and B2P) genotypes show complete separation for TDP status (11/11 vs 0/14), indicating that the two groups represent strongly contrasting phenotypic classes.
With five Br1L1363P samples and no observed TDP-positive cases, we can therefore exclude high TDP prevalence (>~45%) linked to this genotype, although moderate prevalence cannot be ruled out.

To increase the robustness of our statement, we sequenced five additional Br1L1363P samples and did not observe any TDP case in any of these 5 new tumor genomes. Now, with a total of 0/10 TDP Br1L1363P samples, we can exclude with 95% confidence a true TDP prevalence greater than ~25%. This strongly argues against a B1P-like phenotype in Br1L1363P tumors.

A Mann-Whitney U test of TD size distributions revealed the following P values: B1P vs B2P: P=1.4E-22. B1P vs Br1L1363P: P=5.7E-32. B2P vs Br1L1363P: not significant. The data and statistical analysis is presented in the resubmission **Figure 5** of the resubmitted m/s.

5. The Ter-HR reporter is integrated at the Rosa26 locus. Could chromatin context at Rosa26 influence HR outcomes compared with endogenous loci? Please discuss this.
We have noted that the Tus/*Ter* data applies to only one euchromatic locus (line 282).

6. FANCM depletion is used to induce TDs. Do authors predict that other fork remodeling enzymes (SMARCAL1, ZRANB3, HLTF) would produce similar outcomes?
In the development of our original paper on BRCA1-linked Group 1 TDs (PMID: 29168504), we screened for a potential role of SMARCAL1, ZRANB3 or HLTF in Tus/*Ter*-induced TD formation using siRNA-mediated depletion. This was the same screen that identified FANCM and BLM as TD co-suppressors in the absence of BRCA1. We did not observe induction of Tus/*Ter*-induced TDs in *Brca1* Δ11 or Brca1 WT cells depleted of SMARCAL1, ZRANB3 or HLTF. We have not yet tested any of these enzymes genetically (e.g., by studying null alleles in the mES cell system). We have not published this preliminary screen, since the failure to elicit a phenotype following siRNA-mediated depletion is not a definitive finding. However, thus far, it appears that FANCM and BLM are distinctive in their behavior as TD co-suppressors. FANCM and BLM might function as TD co-suppressors through their ability to suppress break-induced replication (BIR—see current **Figure EV5**). Such a function might not involve SMARCAL1, ZRANB3 or HLTF. However, the BIR hypothesis has not been formally tested.

7. Are there enough data points in Fig. 4C to be confident in the statistics?
As noted above, we have performed a third replicate of this experiment and now report the data in **Figure 3**.

Referee #3:

The presence of 10 kb tandem duplications in the genome is a characteristic feature of BRCA1- but not BRCA2-deficient cancers. The authors have previously shown that FANCM deficiency also increases tandem duplications, and that loss of both BRCA1 and FANCM is synthetic lethal. In this manuscript, the authors investigate whether the interaction of BRCA1 with PALB2 is required for suppression of tandem duplications, using a set of BRCA1 coiled-coil domain mutants that abolish PALB2 binding. They find that these mutants are deficient in homologous recombination (HR), but their expression does not cause tandem duplications nor cell death due to loss of FANCM. In contrast, expression of a BRCA1 "exon 11" mutant that is defective in HR due to a large deletion in the central region of BRCA1, does lead to tandem duplications and causes synthetic lethality with FANCM loss. The authors conclude from this that it is the role of BRCA1 in DNA end resection that suppresses tandem duplications rather than its role in loading RAD51 with PALB2-BRCA2.

The manuscript addresses an important question and presents interesting genetic observations. However, the mechanistic interpretation that DNA end resection specifically suppresses tandem duplication formation and determines FANCM synthetic

lethality, is not sufficiently supported by the current data. The BRCA1 exon 11 mutant is not a separation of function mutant, it is defective in multiple functions of BRCA1 (including RAD51 recruitment and HR, as the authors themselves and others have shown) and is partly cytoplasmic due to loss of NLS. The BRCA1-BARD1 complex has many previously described roles in response to DNA damage. The authors therefore cannot conclude that a defect in DNA end resection has anything to do with tandem duplication formation. While they do formally acknowledge this in the Discussion, they definitively state that DNA resection defects are driving TD formation in the title, abstract and text of the manuscript, which they have not established. The manuscript therefore needs substantial revision to take this into account before it should be published. At a minimum, the authors should significantly moderate the mechanistic claim regarding resection, and reframe the study as demonstrating that BRCA1 suppression of TD formation and tolerance to FANCM loss does not require the BRCA1-PALB2 interaction or canonical HR.
Thanks for these well-reasoned points. The referee is correct that any claim regarding the specific DNA end resection function of BRCA1 is provisional. We have therefore modified the title to reflect the interpretation suggested by the referee ('BRCA1 suppression of TD formation and tolerance to FANCM loss does not require the BRCA1-PALB2 interaction or canonical HR.'). However, in light of the inability to exclude a possible role for RAD51 (see discussion of Referee #1 point 17), we cannot strictly claim that TD formation does not involve a RAD51-dependent step, only that BRCA1's RAD51 loading function is not required. We have therefore changed the title to 'The BRCA1 coiled coil domain is dispensable for tandem duplication suppression and tolerance of FANCM loss.'

Main points:

1. Without a BRCA1 mutant that exclusively abolishes DNA end resection, it remains possible that other unidentified functions of BRCA1, which are also affected by exon 11 deletion, could contribute to tandem duplications and synthetic lethality with FANCM. Further studies with more targeted mutations would be needed to conclusively determine the specific role of DNA end resection in this context. For example, the E3 ligase activity of BRCA1-BARD1 has been shown to ubiquitylate histones and promote resection by some groups (Densham et al., NSMB 2016; Wang et al., Mol. Cell 2023), but not others (Nakamura et al., NCB 2019). How does a RING mutation (that maintains BRCA1-BARD1 interaction but abolishes E3 ligase activity) behave in the authors' resection, FANCM synthetic lethality and TD formation assays? Similarly, BARD1 was recently shown to interact with SLX4-XPF (Tsukada et al., Mol. Cell 2024), which the authors themselves have previously linked to regulating Tus/Ter HR (Elango et al., NSMB 2022). How does mutating that motif in BARD1 affect the same assays? Examining these mutants may provide the necessary evidence to prove or disprove the authors hypothesis that BRCA1's role in resection is what is driving tandem duplications and FANCM synthetic lethality.
These are all interesting suggestions. We have begun to generate derivatives of the *Brca1* fl11-HA/– cells that contain pathogenic missense mutations in the RING and tandem BRCT domains of *Brca1*. However, the completion of this work is not feasible

within the time frame of this resubmission. We do not yet have a system for engineering mutant alleles of *Bard1*, and such work is again beyond the scope of this resubmission.

We have added this qualified interpretation at the end of the Results section: "Our data, although limited to a small number of *BRCA1* alleles, suggest that synthetic lethality/sickness following *Fancm* loss in *Brca1* mutants is correlated with defective DNA end resection function but not with overall HR capacity (**Table 1**). Further testing of this hypothesis will require analysis of additional *Brca1* mutant alleles."

2. Fig. 4: the RPA ChIP experiment has only two replicates, two additional ones are needed. There may be a defect with some of the coiled-coiled mutants, but the difference between the current replicates is too great to know for sure.
As noted above (Referee #1 point 4), we have performed a third biological replicate of this experiment and now show only one of these experiments in its raw form, together with statistical analysis of the three replicates.

3. What happens in the authors' resection assay (Fig. 4) when the exon 11 mutant cells are treated with siBRCA1? The BRCA1 BRCTs bind to the RAP80 complex, which suppresses resection. Could the mutant BRCA1 protein be acting in a dominant negative manner to suppress resection?
The referee makes the accurate point (noted in their summary comments above) that we do not yet fully understand how deletion of *Brca1* exon 11 causes a defect in DNA end resection. A dominant negative mechanism is one of several interesting hypotheses that could be explored in future studies—is its action direct (e.g., PMID: 39261728) or indirect, etc. However, it is not the goal of this m/s to determine the mechanism by which *Brca1* exon 11 deletion disables DNA end resection. In this m/s, we are using the *Brca1* Δ11 allele as a well-established (and experimentally tractable) example of a DNA end resection-defective *Brca1* allele (as shown in our experiments in **Figure 3**). Future experiments, using additional *BRCA1* mutant alleles will be needed to further test the hypothesis that the DNA end resection function of BRCA1 suppresses Group 1 TD formation.

Other points:

1. The authors state in the abstract that TDs are drivers of tumorigenesis in BRCA1-deficient cancers, but how do they know this without a separation-of function allele? Surely it is more likely that loss of HR drives tumorigenesis, given that BRCA2, PALB2 and RAD51 paralogues are also breast and ovarian tumour suppressors but they don't suppress TDs?
We apologize for having described *BRCA1*'s contribution to tumorigenesis in insufficient detail in the abstract. (We have removed this statement in the abstract of the revised m/s.) *BRCA1* loss drives tumorigenesis though its HR defect (a phenotype shared with *BRCA2* mutants) and *also* through its Group 1 TD phenotype (a phenotype not shared with *BRCA2* mutants). The evidence in favor of a tumorigenic role for *BRCA1*-linked TDs is that *BRCA1*-linked Group 1 TDs in the human cancer genome are enriched within tumor suppressor genes (TSG), which they disable (for example, by exon

duplication). The concentration of Group 1 TDs within TSGs in the genome of the established cancer likely reflects their positive selection during tumorigenesis. Hence, it is reasonable to consider these TSG-disrupting Group 1 TDs as cancer-promoting alterations.

2. There is significant disagreement in the literature about whether BRCA1 promotes resection at all DSBs (e.g. Densham et al., NSMB 2016), or promotes resection at IR-induced DSBs but not broken replication forks (Pavani et al., Science 2024), or actually inhibits resection (e.g. Dever et al., Aging 2011; Polato et al., J. Exp. Med. 2014), or if BRCA1 has only a minor or no direct role in the process at all (e.g. Escribano-Diaz et al., Mol. Cell 2012; Reczek et al, JCB 2013; Cruz-García et al., Cell Rep. 2014; Ochs et al., NSMB 2016). BRCA1 deficient cells are usually unhealthy. Could the apparent resection defects seen by some groups but not others, be down to more G1 cells and/or the general health of the cells, rather than any direct role of BRCA1 in the process? A more balanced discussion of the disagreements in the literature would be beneficial.
We restrict our study to mouse ES cells where deletion of *Brca1* exon 11 is well-tolerated. We opted not to rely on the complex literature regarding BRCA1-mediated DNA end resection, but to measure it directly in **Figure 3** of the resubmitted m/s.

3. The exact boundaries of the exon-11 deletion and other mutants should be clearly defined (amino acid numbers), and the corresponding human residues should be provided in the manuscript.
We have now included a key in **Figure 1A** that gives the exact amino acid changes in each m*Brca1* mutant allele we studied, and also provides the equivalent amino acid positions of these mutations in human BRCA1.

References

Anantha RW, Simhadri S, Foo TK, Miao S, Liu J, Shen Z, Ganesan S, Xia B (2017) Functional and mutational landscapes of BRCA1 for homology-directed repair and therapy resistance. *eLife* 6

Ceppi I, Dello Stritto MR, Mütze M, Braunshier S, Mengoli V, Reginato G, Võ HMP, Jimeno S, Acharya A, Roy M *et al* (2024) Mechanism of BRCA1-BARD1 function in DNA end resection and DNA protection. *Nature*

Dray E, Etchin J, Wiese C, Saro D, Williams GJ, Hammel M, Yu X, Galkin VE, Liu D, Tsai MS *et al* (2010) Enhancement of RAD51 recombinase activity by the tumor suppressor PALB2. *Nature structural & molecular biology* 17: 1255–1259

Menghi F, Barthel FP, Yadav V, Tang M, Ji B, Tang Z, Carter GW, Ruan Y, Scully R, Verhaak RGW *et al* (2018) The Tandem Duplicator Phenotype Is a Prevalent Genome-Wide Cancer Configuration Driven by Distinct Gene Mutations. *Cancer Cell* 34: 197–210 e195

Menghi F, Inaki K, Woo X, Kumar PA, Grzeda KR, Malhotra A, Yadav V, Kim H, Marquez EJ, Ucar D *et al* (2016) The tandem duplicator phenotype as a distinct genomic configuration in cancer. *Proc Natl Acad Sci U S A* 113: E2373–2382

Salunkhe S, Daley JM, Kaur H, Tomimatsu N, Xue C, Raina VB, Jasper AM, Rogers CM, Li W, Zhou S *et al* (2024) Promotion of DNA end resection by BRCA1-BARD1 in homologous recombination. *Nature*

Sy SM, Huen MS, Chen J (2009) PALB2 is an integral component of the BRCA complex required for homologous recombination repair. *Proc Natl Acad Sci U S A* 106: 7155–7160

Xia B, Sheng Q, Nakanishi K, Ohashi A, Wu J, Christ N, Liu X, Jasin M, Couch FJ, Livingston DM (2006) Control of BRCA2 cellular and clinical functions by a nuclear partner, PALB2. *Mol Cell* 22: 719–729

Zhang F, Ma J, Wu J, Ye L, Cai H, Xia B, Yu X (2009) PALB2 links BRCA1 and BRCA2 in the DNA-damage response. *Curr Biol* 19: 524–529

Dr. Ralph Scully
Beth Israel Deaconess Medical Cntr
Medicine, Division of Hematology
330 Brookline Avenue, CLS-438
Boston, MA 02215

25th Aug 2026

Re: EMBOJ-2026-123909R
The BRCA1 coiled coil domain is dispensable for tandem duplication suppression and tolerance of FANCM loss

Dear Ralph,

Thank you for submitting your revised manuscript on BRCA1 coiled-coil mutations to The EMBO Journal. It has now been again seen by two of the original referees (see comments below), who were overall satisfied with the revisions and raised no further objections. We shall therefore be happy to accept the manuscript for publication, following incorporation of a few remaining editorial points as follows:

- Please adjust the order of the manuscript sections, and also make sure to use the correct section headers:
Single title page with complete author information, Abstract, Introduction, Results, Discussion, Methods, Data Availability, Acknowledgements, Disclosure and Competing Interests Statement, References, Main Figure Legends, Tables, Expanded Figure Legends.

- As we have switched from a free-text author contribution statement towards a more formal statement based on Contributor Role Taxonomy (CRediT) terms, please remove the present Author Contribution section and instead specify each author's contribution(s) directly in the Author Information page of our submission system during upload of the final manuscript. See https://casrai.org/credit/ for more information.

- Please carefully go through the reference list and make sure that all references have their complete citation information - including year, journal name & volume, and page/elocator numbers - such information is currently missing for several of them.

- For preprint citations: Please change in-text reference format according to "preprint: XXX et al, 2025"; and in the reference list, name of the platform plus DOI + preprint label - e.g. "bioRxiv doi: 1234/002.dfj123 [PREPRINT]"

- For the Appendix PDF, please preface it with a title page stating "Appendix for [manuscript title]", and include page numbers for the items listed in the ToC.

- Please note that the Data Availability section should only refer to any newly generated datasets deposited in public repositories; previously published datasets should instead be cited where relevant using our 'data citaiton' format (see our Reference Guidelines for detail: https://link.springer.com/journal/44318/submission-guidelines#cms-Reference-guidelines)

- Please move the Reagents and Tools table from the main article file, and upload it as a separate text file. Also, please make sure to adhere to the template table downloadable from our author guidelines:
https://www.embopress.org/page/journal/14693178/authorguide#structuredmethods

- Please provide suggestions for a short 'blurb' text prefacing and summing up the conceptual aspect of the study in two sentences (max. 250 characters), followed by 3-5 one-sentence 'bullet points' with brief factual statements of key results of the paper; they will form the basis of an editor-written 'Synopsis' accompanying the online version of the article. Please also upload a synopsis image, which can be used as a "visual title" for the synopsis section of your paper. The image should be in JPG format and exactly 550 pixels wide and 300 pixels high, and please make sure that it remains clear enough even in these modest dimensions.

- Finally, during routine pre-acceptance checks, our data editors have raised the following queries regarding figures, data, and legends; I would appreciate if you briefly answered to them in the cover letter of your final submission, and made the requested text modifications with changes/additions highlighted via the "Track changes" option, to facilitate our final checking.
1. Please define the exact p values in the legends of figures 2A-C; 3C, EV3 A-C
2. Please define the box plots in terms of minima, maxima, centre, bounds of box and whiskers, and percentile in the legends of figures 5A, C
3. Please provide information related to 'n' in the legends of figures 5C, EV2 C
4. Please define the error bars in the legends of figures 3C, 4A, B; 6B, EV2 C.

I am returning the manuscript to you for a final round of minor revision, solely to allow you to make these modifications and upload the revised files. Once we will have received them, we should be ready to swiftly proceed with formal acceptance and production of the manuscript.

With kind regards,

Hartmut

Hartmut Vodermaier, PhD
Senior Editor, The EMBO Journal
h.vodermaier@embojournal.org

*** PLEASE NOTE: All revised manuscript are subject to initial checks for completeness and adherence to our formatting guidelines. Revisions may be returned to the authors and delayed in their editorial re-evaluation if they fail to comply to the following requirements. As a first step please read our guidelines for revised submissions: https://link.springer.com/journal/44318/submission-guidelines#cms-Revised-submissions

1) Every manuscript requires a Data Availability section (even if only stating that no deposited datasets are included). Primary datasets or computer code produced in the current study have to be deposited in appropriate public repositories prior to resubmission, and reviewer access details provided in case that public access is not yet allowed.

2) Each figure legend must specify
- size of the scale bars that are mandatory for all micrograph panels
- the statistical test used to generate error bars and P-values
- the type error bars (e.g., S.E.M., S.D.)
- the number (n) and nature (biological or technical replicate) of independent experiments underlying each data point
- Figures may not include error bars for experiments with n<3; scatter plots showing individual data points should be used instead.

3) Revised manuscript text (including main tables, and figure legends for main and EV figures) has to be submitted as editable text file (e.g., .docx format). We encourage highlighting of changes (e.g., via text color) for the referees' reference.

4) Each main and each Expanded View (EV) figure should be uploaded as individual production-quality files (preferably in .eps, .tif, .jpg formats). For suggestions on figure preparation/layout, please refer to our Figure Preparation Guidelines: https://media.springernature.com/original/springer-cms/rest/v1/content/27825798/data/v1

5) Point-by-point response letters should include the original referee comments in full together with your detailed responses to them (and to specific editor requests if applicable), and also be uploaded as editable (e.g., .docx) text files.

6) Please complete our Author Checklist, and make sure that information entered into the checklist is also reflected in the manuscript; the checklist will be available to readers as part of the Review Process File.

7) All authors listed as (co-)corresponding need to deposit, in their respective author profiles in our submission system, a unique ORCiD identifier linked to their name. Please see our Guide to Authors for detailed instructions.

8) Please note that supplementary information at EMBO Press has been superseded by the 'Expanded View' for inclusion of additional figures, tables, movies or datasets; with up to five EV Figures being typeset and directly accessible in the HTML version of the article.

9) To facilitate reproducibilty and cross-laboratory adoption of methodologies, please structure the Materials & Methods section as outlined in our guide to authors, including a completed Reagents and Tools Table.

10) Digital image enhancement is acceptable practice, as long as it accurately represents the original data and conforms to community standards. If a figure has been subjected to significant electronic manipulation, this must be clearly noted in the figure legend and/or the 'Materials and Methods' section. The editors reserve the right to request original versions of figures and the original images that were used to assemble the figure. Finally, we generally encourage uploading of numerical as well as gel/blot image source data.

At EMBO Press we ask authors to provide source data for the main manuscript figures. You will receive a separate email with instructions for providing source data with your revised manuscript, including how to upload and organize the files.

In the interest of ensuring the conceptual advance provided by the work, we recommend submitting a revision within 3 months (23rd Nov 2026). Please discuss the revision progress ahead of this time with the editor if you require more time to complete the revisions. Use the link below to submit your revision:

Link Not Available

Referee #1:

The authors were responsive to all major concerns present in the prior critiques. I am in favor of acceptance given the timely nature of the work, and its clarification of mechanism of action for tandem duplications and their relevance to synthetic lethal relationships between some BRCA1 mutants and FANCM loss.

Referee #3:

The authors have addressed my points, mostly with textual changes that are welcome and have improved the manuscript. I do think the authors are still too attached to their hypothesis of BRCA1 role in DNA end resection being responsible for suppressing tandem duplications, and rather resistant to considering other mechanisms. However, that is a matter for future debate and I think the study is fine to be published as it is.

EMBO Press Author Checklist

Corresponding Author Name: Ralph Scully	
Journal Submitted to: The EMBO Journal	
Manuscript Number: EMBOJ-2026-123909R	

USEFUL LINKS FOR COMPLETING THIS FORM
The EMBO Journal - Author Guidelines
EMBO Reports - Author Guidelines
Molecular Systems Biology - Author Guidelines
EMBO Molecular Medicine - Author Guidelines

Reporting Checklist for Life Science Articles (updated January
This checklist is adapted from Materials Design Analysis Reporting (MDAR) Checklist for Authors. MDAR establishes a minimum set of requirements in transparent reporting in the life sciences (see Statement of Task: 10.31222/osf.io/9sm4x). Please follow the journal's guidelines in preparing your
Please note that a copy of this checklist will be published alongside your article.

Abridged guidelines for figures
1. Data
The data shown in figures should satisfy the following conditions:
- the data were obtained and processed according to the field's best practice and are presented to reflect the results of the experiments in an accurate and unbiased manner.
- ideally, figure panels should include only measurements that are directly comparable to each other and obtained with the same assay.
- plots include clearly labeled error bars for independent experiments and sample sizes. Unless justified, error bars should not be shown for technical
- if n<5, the individual data points from each experiment should be plotted. Any statistical test employed should be justified.
- Source Data should be included to report the data underlying figures according to the guidelines set out in the authorship guidelines on Data

2. Captions
Each figure caption should contain the following information, for each panel where they are relevant:
- a specification of the experimental system investigated (eg cell line, species name).
- the assay(s) and method(s) used to carry out the reported observations and measurements.
- an explicit mention of the biological and chemical entity(ies) that are being measured.
- an explicit mention of the biological and chemical entity(ies) that are altered/varied/perturbed in a controlled manner.
- the exact sample size (n) for each experimental group/condition, given as a number, not a range;
- a description of the sample collection allowing the reader to understand whether the samples represent technical or biological replicates (including how many animals, litters, cultures, etc.).
- a statement of how many times the experiment shown was independently replicated in the laboratory.
- definitions of statistical methods and measures:
  - common tests, such as t-test (please specify whether paired vs. unpaired), simple χ2 tests, Wilcoxon and Mann-Whitney tests, can be unambiguously identified by name only, but more complex techniques should be described in the methods section;
  - are tests one-sided or two-sided?
  - are there adjustments for multiple comparisons?
  - exact statistical test results, e.g., P values = x but not P values < x;
  - definition of 'center values' as median or average;
  - definition of error bars as s.d. or s.e.m.

Please complete ALL of the questions below.
Select "Not Applicable" only when the requested information is not relevant for your study.

Materials

Newly Created Materials	Information included in the manuscript?	In which section is the information available? (Reagents and Tools Table, Materials and Methods, Figures, Data Availability Section)
New materials and reagents need to be available; do any restrictions apply?	Not Applicable	

Antibodies	Information included in the manuscript?	In which section is the information available? (Reagents and Tools Table, Materials and Methods, Figures, Data Availability Section)
For **antibodies** provide the following information:<br>- Commercial antibodies: RRID (if possible) or supplier name, catalogue number and or/clone number<br>- Non-commercial: RRID or citation	Yes	

DNA and RNA sequences	Information included in the manuscript?	In which section is the information available? (Reagents and Tools Table, Materials and Methods, Figures, Data Availability Section)
**Short novel DNA or RNA including primers, probes**: provide the sequences.	Yes	

Cell materials	Information included in the manuscript?	In which section is the information available? (Reagents and Tools Table, Materials and Methods, Figures, Data Availability Section)
**Cell lines:** Provide species information, strain. Provide accession number in repository **OR** supplier name, catalog number, clone number, and/**OR** RRID.	Yes	
**Primary cultures:** Provide species, strain, sex of origin, genetic modification status.	Yes	
Report if the cell lines were recently **authenticated** (e.g., by STR profiling) and tested for mycoplasma contamination.	Yes	

Experimental animals	Information included in the manuscript?	In which section is the information available? (Reagents and Tools Table, Materials and Methods, Figures, Data Availability Section)
**Laboratory animals or Model organisms:** Provide species, strain, sex, age, genetic modification status. Provide accession number in repository **OR** supplier name, catalog number, clone number, **OR** RRID.	Yes	
**Animal observed in or captured from the field:** Provide species, sex, and age where possible.	Not Applicable	
Please detail **housing and husbandry conditions**.	Not Applicable	

Plants and microbes	Information included in the manuscript?	In which section is the information available? (Reagents and Tools Table, Materials and Methods, Figures, Data Availability Section)
**Plants:** provide species and strain, ecotype and cultivar where relevant, unique accession number if available, and source (including location for collected wild specimens).	Not Applicable	
**Microbes:** provide species and strain, unique accession number if available, and source.	Not Applicable	

Human research participants	Information included in the manuscript?	In which section is the information available? (Reagents and Tools Table, Materials and Methods, Figures, Data Availability Section)
If collected and within the bounds of privacy constraints report on age, sex and gender or ethnicity for all study participants.	Not Applicable	

Core facilities	Information included in the manuscript?	In which section is the information available? (Reagents and Tools Table, Materials and Methods, Figures, Data Availability Section)
If your work benefited from core facilities, was their service mentioned in the acknowledgments section?	Yes	

Design

Study protocol	Information included in the manuscript?	In which section is the information available? (Reagents and Tools Table, Materials and Methods, Figures, Data Availability Section)
If study protocol has been **pre-registered, provide DOI in the manuscript**. For clinical trials, provide the trial registration number **OR** cite DOI.	Not Applicable	
Report the **clinical trial registration number** (at ClinicalTrials.gov or equivalent), where applicable.	Not Applicable	

Laboratory protocol	Information included in the manuscript?	In which section is the information available? (Reagents and Tools Table, Materials and Methods, Figures, Data Availability Section)
Provide DOI OR other citation details if **external detailed step-by-step protocols** are available.	Not Applicable	

Experimental study design and statistics	Information included in the manuscript?	In which section is the information available? (Reagents and Tools Table, Materials and Methods, Figures, Data Availability Section)
Include a statement about **sample size** estimate even if no statistical methods were used.	Yes	
Were any steps taken to minimize the effects of subjective bias when allocating animals/samples to treatment (e.g. **randomization procedure**)? If yes, have they been described?	Not Applicable	
Include a statement about **blinding** even if no blinding was done.	Not Applicable	
Describe **inclusion/exclusion criteria** if samples or animals were excluded from the analysis. Were the criteria pre-established? If sample or data points were omitted from analysis, report if this was due to attrition or intentional exclusion and provide justification.	Not Applicable	
For every figure, are **statistical tests** justified as appropriate? Do the data meet the assumptions of the tests (e.g., normal distribution)? Describe any methods used to assess it. Is there an estimate of variation within each group of data? Is the variance similar between the groups that are being statistically compared?	Yes	

Sample definition and in-laboratory replication	Information included in the manuscript?	In which section is the information available? (Reagents and Tools Table, Materials and Methods, Figures, Data Availability Section)
In the figure legends: state number of times the experiment was **replicated** in laboratory.	Yes	
In the figure legends: define whether data describe **technical or biological replicates**.	Yes	

Ethics

Ethics	Information included in the manuscript?	In which section is the information available? (Reagents and Tools Table, Materials and Methods, Figures, Data Availability Section)
Studies involving **human participants**: State details of **authority granting ethics approval** (IRB or equivalent committee(s), provide reference number for approval.	Not Applicable	
Studies involving **human participants**: Include a statement confirming that **informed consent** was obtained from all subjects and that the experiments conformed to the principles set out in the WMA Declaration of Helsinki and the Department of Health and Human Services Belmont Report.	Not Applicable	
Studies involving **human participants:** For publication of **patient photos**, include a statement confirming that consent to publish was obtained.	Not Applicable	
Studies involving experimental **animals**: State details of **authority granting ethics approval** (IRB or equivalent committee(s), provide reference number for approval. Include a statement of compliance with ethical regulations.	Not Applicable	
Studies involving **specimen and field samples:** State if relevant **permits** obtained, provide details of authority approving study; if none were required, explain why.	Not Applicable	

Dual Use Research of Concern (DURC)	Information included in the manuscript?	In which section is the information available? (Reagents and Tools Table, Materials and Methods, Figures, Data Availability Section)
Could your study fall under dual use research restrictions? Please check biosecurity documents and list of **select agents and toxins** (CDC): https://www.selectagents.gov/sat/list.htm	Not Applicable	
If you used a select agent, is the security level of the lab appropriate and reported in the manuscript?	Not Applicable	
If a study is subject to dual use research of concern regulations, is the name of the **authority granting approval and reference number** for the regulatory approval provided in the manuscript?	Not Applicable	

Reporting

The MDAR framework recommends adoption of discipline-specific guidelines, established and endorsed through community initiatives. Journals have their own policy about requiring specific guidelines and recommendations to complement MDAR.

Adherence to community standards	Information included in the manuscript?	In which section is the information available? (Reagents and Tools Table, Materials and Methods, Figures, Data Availability Section)
State if relevant guidelines or checklists (e.g., **ICMJE, MIBBI, ARRIVE, PRISMA**) have been followed or provided.	Not Applicable	
For **tumor marker prognostic studies,** we recommend that you follow the **REMARK** reporting guidelines (see link list at top right). See author guidelines, under 'Reporting Guidelines'. Please confirm you have followed these guidelines.	Not Applicable	
For **phase II and III randomized controlled trials**, please refer to the **CONSORT** flow diagram (see link list at top right) and submit the CONSORT checklist (see link list at top right) with your submission. See author guidelines, under 'Reporting Guidelines'. Please confirm you have submitted this list.	Not Applicable	

Data Availability

Data availability	Information included in the manuscript?	In which section is the information available? (Reagents and Tools Table, Materials and Methods, Figures, Data Availability Section)
Have **primary datasets** been deposited according to the journal's guidelines (see 'Data Deposition' section) and the respective accession numbers provided in the Data Availability Section?	Yes	
Were **human clinical and genomic datasets** deposited in a public access-controlled repository in accordance to ethical obligations to the patients and to the applicable consent agreement?	Not Applicable	
Are **computational models** that are central and integral to a study available without restrictions in a machine-readable form? Were the relevant accession numbers or links provided?	Yes	
If publicly available data were reused, provide the respective **data citations in the reference list**.	Yes	
