# Supplementary material for: The BRCA1 coiled-coil domain is dispensable for suppression of tandem duplications and tolerance of FANCM loss
Source: EMBO J. 2026 Sep 18;45(20):7136–62. doi: 10.1038/s44318-026-00913-x (PMC13672311; doi:10.1038/s44318-026-00913-x)
Supplement: Supplementary file 3 [file 44318_2026_913_MOESM8_ESM.pdf]

Expanded View Figures

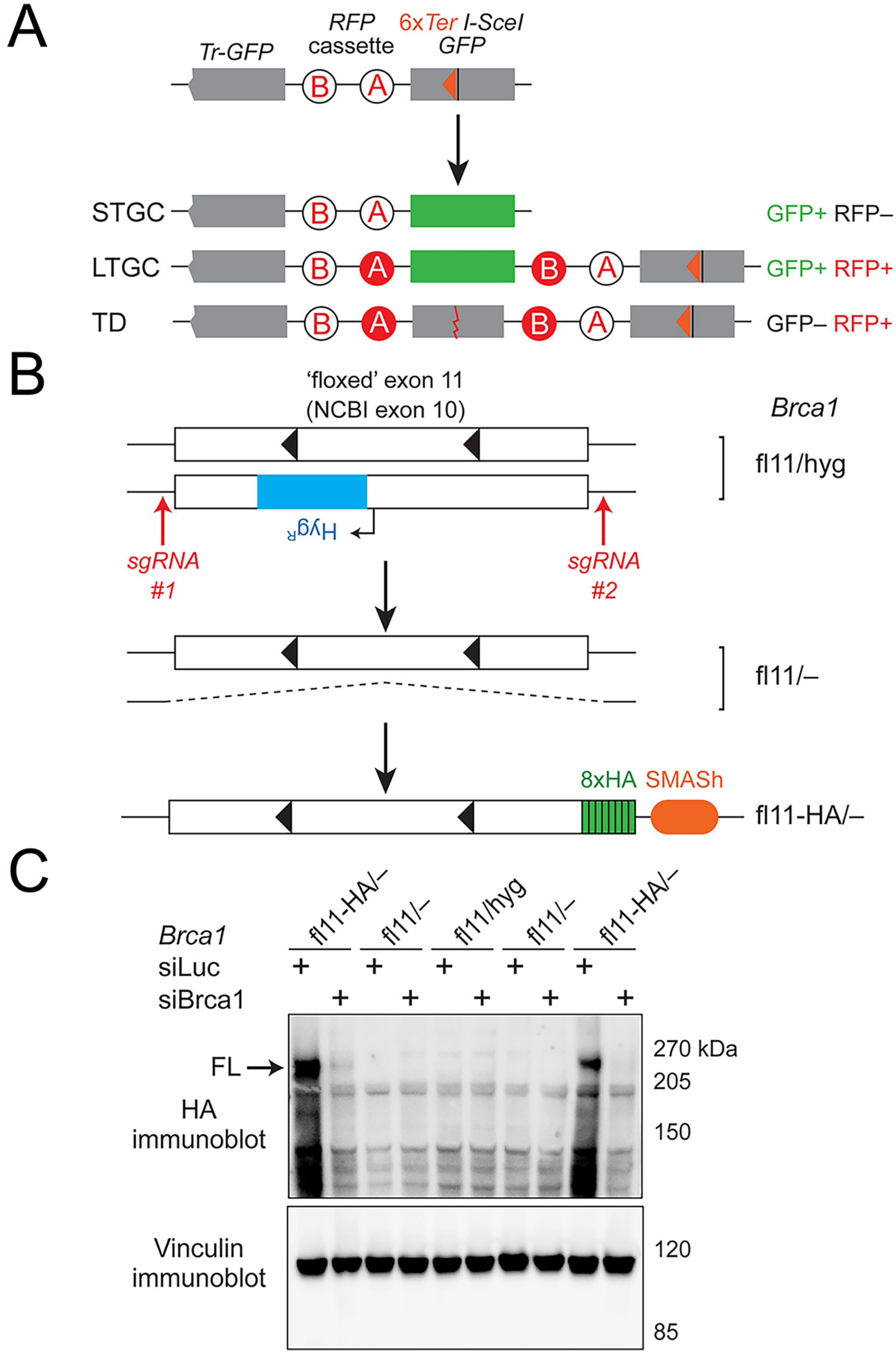

Figure EV1. A haploid system for genetic analysis of *Brca1* in stalled fork repair.

(**A**) Schematic of *Ter*-HR reporter for quantitation of Tus/*Ter*-induced and DSB-induced HR. *Tr-GFP*: 5′ truncated *GFP* allele acts as a donor during HR. *RFP* cassette elements A and B: artificial exons encoding WT *RFP*, placed in unproductive orientation. Promoters and polyadenylation signals not shown. STGC short tract gene conversion, LTGC long tract gene conversion, TD Group 1 tandem duplication. Each repair product is associated with fluorescent products, as shown. Red line in TD product: non-homologous TD breakpoint junction. (**B**) Outline of method for generating haploid mES cells expressing *Brca1*-HA. Black triangles: *LoxP* sites flanking *Brca1* exon 11 (NCBI exon 10). The deleted *Brca1* allele is not shown in the cartoon of $Brca1^{fl11\text{-}HA/-}$ allele. (**C**) Identification of HA-tagged gene product in $Brca1^{fl11\text{-}HA/-}$ cells. Upper panel: anti-HA immunoblotting of whole cell extracts. Note the abolition of the BRCA1-HA signal by *Brca1* siRNA but not by control *Luciferase* siRNA (siLuc). FL full-length *Brca1* gene product. Lower panel: Vinculin loading control.

© The Author(s)

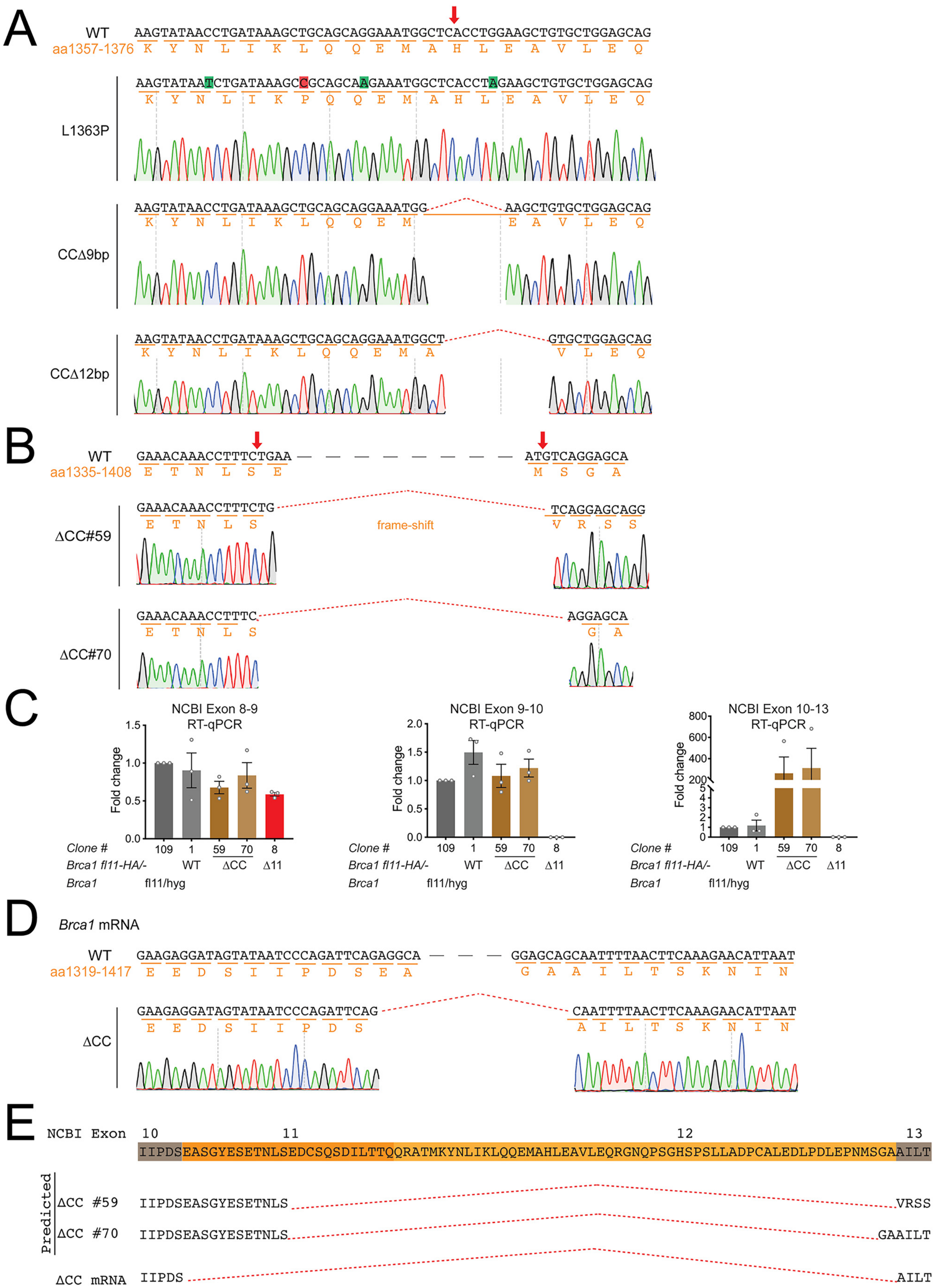
A
WT
aa1357-1376
AAGTATAACCTGATAAAGCTGCAGCAGGAAATGGCTCACCTGGAAGCTGTGCTGGAGCAG
K Y N L I K L Q Q E M A H L E A V L E Q
L1363P
AAGTATAATCTGATAAAGCCGCAGCAAGAAATGGCTCACCTAGAAGCTGTGCTGGAGCAG
K Y N L I K P Q Q E M A H L E A V L E Q
CCΔ9bp
AAGTATAACCTGATAAAGCTGCAGCAGGAAATGG AAGCTGTGCTGGAGCAG
K Y N L I K L Q Q E M E A V L E Q
CCΔ12bp
AAGTATAACCTGATAAAGCTGCAGCAGGAAATGGCT GTGCTGGAGCAG
K Y N L I K L Q Q E M A V L E Q
B
WT
aa1335-1408
GAAACAAACCTTTCTGAA ATGTCAGGAGCA
E T N L S E M S G A
ΔCC#59
GAAACAAACCTTTCTG TCAGGAGCAGG
E T N L S V R S S
frame-shift
ΔCC#70
GAAACAAACCTTTC AGGAGCA
E T N L S G A
C
NCBI Exon 8-9
RT-qPCR
NCBI Exon 9-10
RT-qPCR
NCBI Exon 10-13
RT-qPCR
Fold change
Clone # 109 1 59 70 8
Brca1 fl11-HA/- WT ΔCC Δ11
Brca1 fl11/hyg
D
Brca1 mRNA
WT
aa1319-1417
GAAGAGGATAGTATAATCCCAGATTCAGAGGCA GGAGCAGCAATTTTAACTTCAAAGAACATTAAT
E E D S I I P D S E A G A A I L T S K N I N
ΔCC
GAAGAGGATAGTATAATCCCAGATTCAG CAATTTTAACTTCAAAGAACATTAAT
E E D S I I P D S A I L T S K N I N
E
NCBI Exon 10 11 12 13
IIPDSEASGYESETNLSEDCSQSDILTTQQRATMKYNLIKLQQEMAHLEAVLEQRGNQPSGHSPSLLADPCALEDLPDLEPNMSGAAILT
Predicted
ΔCC #59 IIPDSEASGYESETNLS VRSS
ΔCC #70 IIPDSEASGYESETNLS GAAILT
ΔCC mRNA IIPDS AILT

◀ **Figure EV2. Sequence analysis of *Brca1* CC mutant clones and exon skipping in *Brca1*$^{\Delta CC\text{-}HA/-}$ cells.**

Brca1 allele abbreviations as in Fig. 1A. Nucleotide sequence is shown in black and amino acids in orange. (**A**) Nucleotide sequence of mouse *Brca1* NCBI exon 12 encoding amino acids 1357-1376 (equivalent residues in human BRCA1: 1401-1420). The red arrow shows the predicted site of Cas9-induced DSB for the generation of CC mutants. The red dotted line denotes deletion of nucleotides. Sequences of *Brca1* p.L1363P, *Brca1* CCΔ9 bp, and *Brca1* CCΔ12 bp alleles are shown. L to P mutation in *Brca1* p.L1363P mutants is highlighted in red. Engineered silent mutations in potential sgRNA PAM sites are highlighted in green. (**B**) *Brca1* NCBI exon 11 and 12 nucleotide sequence encoding amino acids 1335 to 1408. ΔCC clone #59 has a frame-shift mutation, whereas clone #70 is in-frame. (**C**) RT-qPCR results showing amplification across three different exon-exon junctions (normalized to parental *Brca1*$^{f11/hyg}$ clone #109). Note the absence of NCBI exons 10–11 amplification in Δ11 mutant clones and over-representation of NCBI exon 10–13 RT-qPCR product in ΔCC clones, reflecting exon skipping. Data show mean and s.e.m. of three independent biological repeats ($n = 3$). (**D**) Sequence of WT *Brca1* cDNA and *Brca1* ΔCC cDNA encompassing NCBI exons 10–13. The black dashed line represents the continuation of the sequence. The red dotted line represents the deletion of nucleotides. ΔCC clones #59 and #70 revealed identical cDNA sequences, consistent with skipping of NCBI exons 11 and 12 in the mRNA and cDNA. (**E**) BRCA1 amino acid sequence region encoded by the 3′ end of *Brca1* NCBI exon 10 to the 5′ end of NCBI exon 13. Amino acids encoded by NCBI exons 10 and 13 are highlighted in gray. Amino acids encoded by NCBI exons 11 and 12 are highlighted in orange and yellow, respectively. Predicted amino acid sequence based on sequencing of *Brca1* gDNA is shown for each ΔCC clone. Actual amino acid sequence based on *Brca1* ΔCC cDNA sequencing shows in-frame deletion resulting from skipping of NCBI exons 11–12.

© The Author(s)

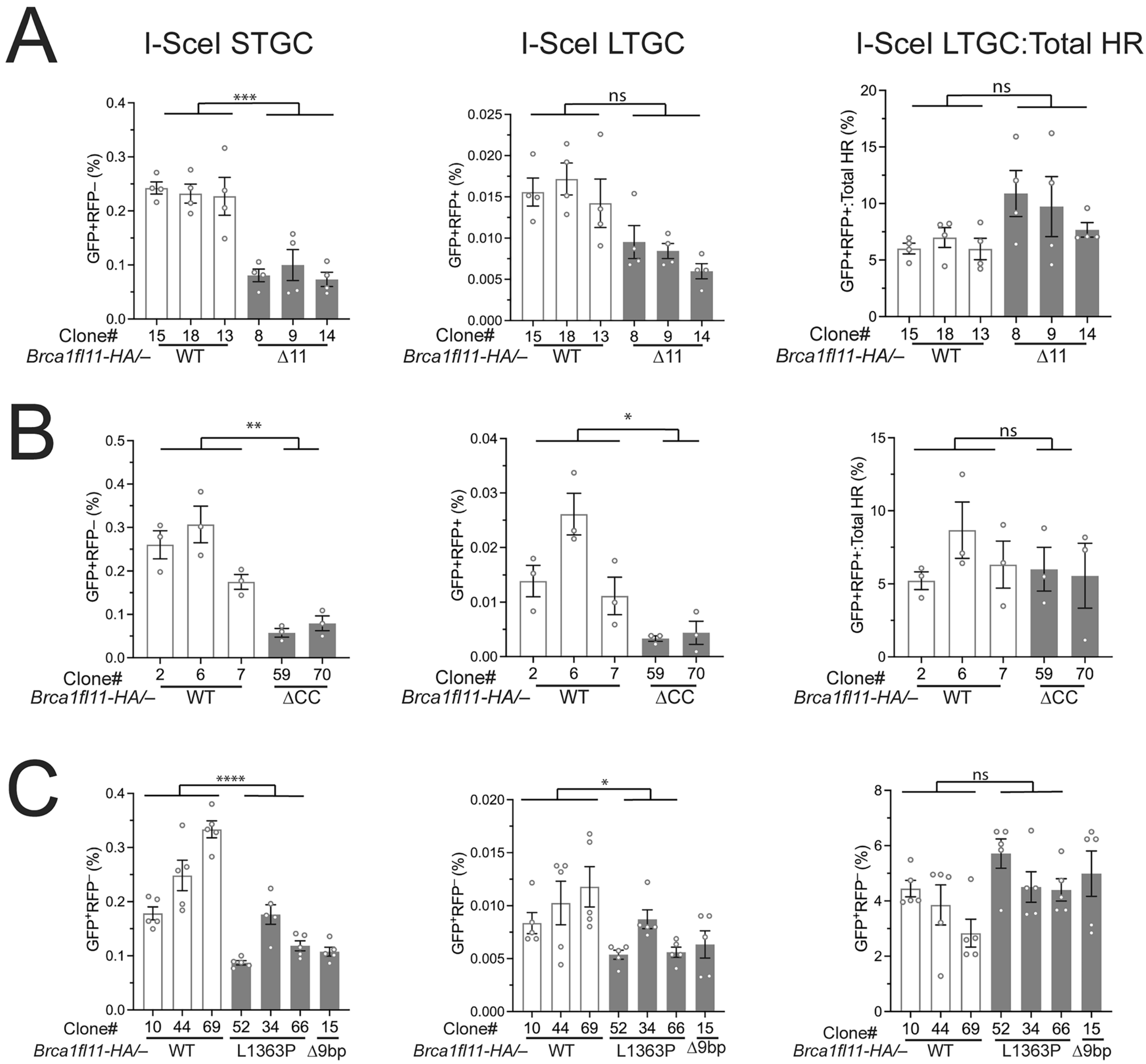

Figure EV3. DSB-induced HR defects in *Brca1* mutants.

(**A**, **C**) DSB-induced HR was quantified in *Brca1* mutants *vs.* isogenic *Brca1*$^{fl11\text{-}HA/-}$ clones. *Brca1* allele abbreviations as in Fig. 1A. Data show mean and s.e.m. of *n* independent experiments. (**A**) *Brca1*$^{\Delta 11\text{-}HA/-}$ cells ($n = 4$). (*P* values: STGC 0.0004; LTGC 0.0635; LTGC:Total HR 0.1513). (**B**) *Brca1*$^{\Delta CC\text{-}HA/-}$ cells ($n = 3$) (*P* values: STGC 0.0081; LTGC 0.0378; LTGC:Total HR 0.5448). (**C**) *Brca1*$^{L1363P\text{-}HA/-}$ and *Brca1*$^{CC\Delta 9bp\text{-}HA/-}$ cells ($n = 5$). (*P* values: STGC < 0.0001; LTGC 0.0352; LTGC:Total HR 0.0802). Statistical analysis: RM-one way ANOVA with Geisser–Greenhouse correction: ns: not significant.

 © The Author(s)

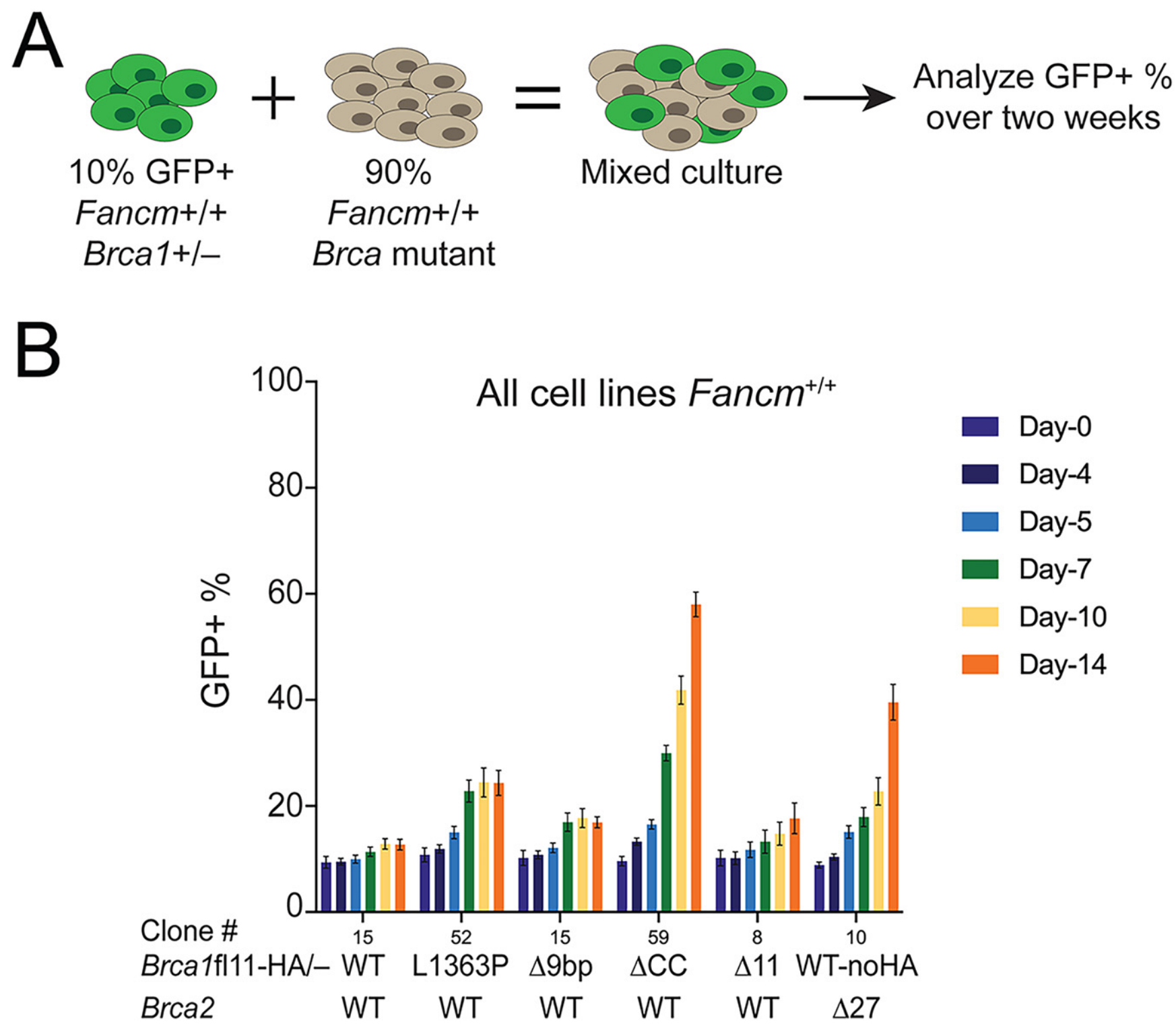

Figure EV4. Controls for Fig. 6: competitive growth of *Brca* mutant cell lines on *Fancm* WT background.

(**A**) Schematic outline of competitive growth assay; all cells are *Fancm*$^{+/+}$. (**B**) Competitive growth assays of different *Fancm*$^{+/+}$;*Brca* genotypes. Bars show mean and s.e.m. of GFP^{+} % at the timepoints shown across 6 independent experiments ($n = 6$). Note degree of enrichment of GFP^{+}-marked *Fancm*$^{+/+}$;*Brca1*$^{fl11\text{-}HA/-}$ cells when mixed with different *Fancm*$^{+/+}$;*Brca* mutant cells. Comparison with Fig. 6 shows that deletion of *Fancm* alters competitive growth properties, disadvantaging *Brca1*$^{\Delta 11\text{-}HA/-}$ cells but improving the relative growth of *Brca1* CC mutants and *Brca2*$^{\Delta 27/\Delta 27}$ cells.

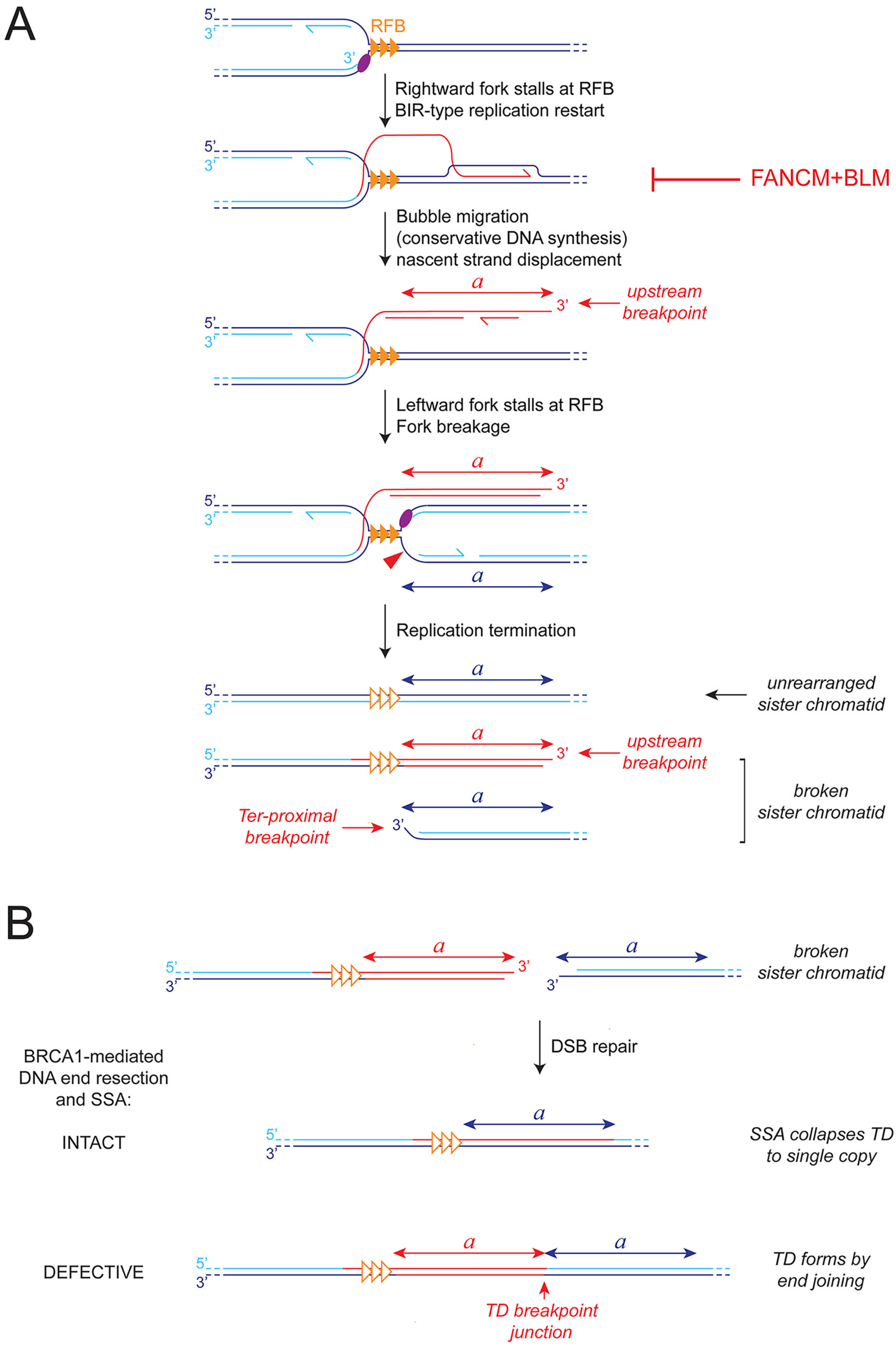
A
5'
3'
RFB
3'
Rightward fork stalls at RFB
BIR-type replication restart
FANCM+BLM
Bubble migration
(conservative DNA synthesis)
nascent strand displacement
a
3'
upstream
breakpoint
Leftward fork stalls at RFB
Fork breakage
Replication termination
unrearranged
sister chromatid
broken
sister chromatid
Ter-proximal
breakpoint
B
DSB repair
BRCA1-mediated
DNA end resection
and SSA:
INTACT
SSA collapses TD
to single copy
DEFECTIVE
TD forms by
end joining
TD breakpoint
junction

 © The Author(s)

Figure EV5. Proposed mechanisms of action of FANCM and BRCA1 in suppression of Tus/*Ter*-induced Group 1 TDs.

(**A**) Interaction of converging replication forks with Tus/*Ter* replication fork barrier (RFB) sets up the Group 1 TD intermediate. The Tus/*Ter*-induced TD breakpoint junction is formed from one breakpoint in close proximity to the *Ter* array (the '*Ter*-proximal' breakpoint) and one 'upstream' breakpoint several kilobases from the *Ter* array (Willis et al, 2017). We infer that for one fork (the rightward fork shown here), replication extends beyond the Tus/*Ter* RFB by an aberrant replication restart mechanism, while the second fork (here, the leftward fork) stalls and is processed at the Tus/*Ter* RFB site. We propose that the rightward fork stalls at the RFB and is restarted as a migrating bubble (i.e., BIR-type conservative DNA synthesis; red). This process could be *RAD51*-dependent or -independent (Panday et al, 2021; Scully et al, 2021). FANCM, in partnership with BLM, suppresses this form of replication restart by disassembling migrating bubble BIR intermediates. Eventual termination of BIR generates a displaced nascent strand that spans the potentially duplicated segment (*a*). The 3′ end of the displaced nascent strand forms the potential upstream breakpoint of the TD. The leftward fork stalls at the RFB and either undergoes fork breakage as shown or fork reversal (not shown). The resulting solitary DNA end forms the potential *Ter*-proximal breakpoint. Completion of DNA replication through the RFB generates one intact sister chromatid and one broken sister chromatid that contains two copies of the TD segment *a*, flanking the break site. (**B**) BRCA1 acts on the broken sister chromatid to suppress Group 1 TD formation. If BRCA1-mediated DNA end resection and SSA functions are intact, the two copies of the duplicated segment *a* flanking the break site are collapsed to a single copy by BRCA1-mediated SSA. If BRCA1-mediated SSA is disabled, end joining opportunistically repairs the break, generating a Group 1 TD. The breakpoint junction is formed by end joining between the upstream breakpoint and the *Ter*-proximal breakpoint.
